# Supplementary material for: Dietary diversity modification through school-based nutrition education among Bangladeshi adolescent girls: A cluster randomized controlled trial
Source: PLoS One. 2023 Mar 8;18(3):e0282407. doi: 10.1371/journal.pone.0282407 (PMC9994752; doi:10.1371/journal.pone.0282407)
Supplement: S1 File — (PDF) [file pone.0282407.s002.pdf]

	**RRC APPLICATION FORM**

RESEARCH PROTOCOL
Number: PR-19089
Version No. 0.02
Version date: 05-08-2019

FOR OFFICE USE ONLY

RRC Approval:	☒ Yes	☐ No	Date: 22/8/2019
ERC Approval:	☐ Yes	☐ No	Date:
AEEC Approval:	☐ Yes	☐ No	Date:
External IRB Approval	☐ Yes	☐ No	Date:

Name of External IRB: ____

Protocol Title:* (maximum 250 characters including space**)** **A Cluster Randomized Controlled Trial to Measure the Efficacy of School-Based Nutrition Education in Improving Dietary Diversity among Bangladeshi Adolescent Girls**

Short Title: (maximum **100** characters including space) **School-Based Nutrition Education in Improving Dietary Diversity among Adolescent Girls**

Key Words:* Nutrition education, dietary diversity, adolescent girls

Name of the Research Division Hosting the Protocol:*

☐ Health Systems and Population Studies Division (HSPSD)
☒ Nutrition and Clinical Services Division (NCSD)
☐ Infectious Diseases Division (IDD)
☐ Maternal and Child Health Division (MCHD)
☐ Laboratory Sciences and Services Division (LSSD)
☐ Other (specify) ______________________________

Has the Protocol been Derived from an Activity:* ☐ No ☒ Yes (please provide following information):

Activity No. : ACT-01031
Activity Title: Improving Dietary Diversity of Adolescent Girls
PI: Mr. Mahfuzur Rahman
Grant No.: GR-00000 Budget Code: 21225108 Start Date: 01/7/2019 End Date: 31/12/2019

icddr,b Strategic Priority/ Initiative (SP 2015-8):* (check all that apply)

☐ Reducing maternal and neonatal mortality
☐ Controlling enteric and respiratory infections
☒ Preventing and treating maternal and childhood malnutrition
☐ Detecting and controlling emerging and re-emerging infections
☐ Achieving universal health coverage
☐ Examining the health consequences of climate change
☐ Preventing and treating non-communicable diseases
☒ Others (specify) Improving Adolescent Nutrition

Research Phase (4 Ds):* (check all that apply**)**

☒ Discovery
☐ Development
☒ Delivery
☒ Evaluation of Delivery

Anticipated Impact of Research:* (check all that apply and please provide details below**)**

☒ Knowledge Production
☒ Capacity Building
☒ Informing Policy
☐ Health and Health Sector Benefits
☐ Economic Benefits

Please provie details here:

Which of the Sustainable Development Goal This Protocol Relates to?:* (check all that apply**)**

- ☐ 1. End poverty in all its forms everywhere
- ☒ 2. End hunger, achieve food security and improved nutrition and promote sustainable agriculture
- ☒ 3. Ensure healthy lives and promote well-being for all at all ages
- ☒ 4. Ensure inclusive and equitable quality education and promote lifelong learning opportunities for all
- ☐ 5. Achieve gender equality and empower all women and girls
- ☐ 6. Ensure availability and sustainable management of water and sanitation for all
- ☐ 7. Ensure access to affordable, reliable, sustainable and modern energy for all
- ☐ 8. Promote sustained, inclusive and sustainable economic growth, full and productive employment and decent work for all
- ☐ 9. Build resilient infrastructure, promote inclusive and sustainable industrialization and foster innovation
- ☐ 10. Reduce inequality within and among countries
- ☐ 11. Make cities and human settlements inclusive, safe, resilient and sustainable
- ☐ 12. Ensure sustainable consumption and production patterns
- ☐ 13. Take urgent action to combat climate change and its impacts
- ☐ 14. Conserve and sustainably use the oceans, seas and marine resources for sustainable development
- ☐ 15. Protect, restore and promote sustainable use of terrestrial ecosystems, sustainably manage forests, combat desertification, and halt and reverse land degradation and halt biodiversity loss
- ☐ 16. Promote peaceful and inclusive societies for sustainable development, provide access to justice for all and build effective, accountable and inclusive institutions at all levels
- ☐ 17. Strengthen the means of implementation and revitalize the global partnership for sustainable development

**Does this Protocol Use the Gender Framework:*** (Please visit: http://shetu.icddrb.org/index.php?option=com_content&view=article&id=265&Itemid=677 for Gender Alanysis Tool with instructions)	☒ Yes (please complete Gender Analysis Tool)<br>☐ No
If 'no' is the response, its reason(s) in brief:	
**Will this Research Specifically Benefit the Disadvantaged (**economically, socially and/or otherwise**):**	☒ Yes<br>☐ No
**Does this Protocol use Behaviour Change Communication:**	☒ Yes<br>☐ No
**Principal Investigator (Should be icddr,b staff):*** Sex ☐ Female ☒ Male<br>**Mr. Mahfuzur Rahman**<br>(Position, phone no, extension no, cell, and email address**):**<br>Research Investigator, Extension: 2594, Cell: 01816543711, email: mahfuzur.rahman@icddrb.org<br>Do you have ethics certification? ☐ No ☒ Yes (please attach in your CV below)<br>Do you have RBM training certification? ☒ No ☐ Yes (please attach the certificate with CV below)	Primary Scientific Division of the PI NCSD
**Co-Principal Investigator(s) Internal**: Sex ☒ Female ☐ Male<br>**Dr. Zannatun Nyma**<br>(Position, phone no, extension no, cell, and email address**):**<br>Research Fellow, email: zannatun.nyma @icddrb.org<br><br>Signature or written consent of Co-PI: ___________________<br>(electronic signature or email or any sort of written consent)<br>[if more than one, please copy and paste this row for additional Co-PIs]<br><br>Do you have ethics certification? ☐ No ☒ Yes (please attach in your CV below)<br>Do you have RBM training certification? ☐ No ☐ Yes (please attach the certificate with CV below)	Primary Scientific Division/ Programme of the Co-PI NCSD<br><br>_____________________________<br>Approval of the Respective Senior Director/ Programme Head<br><br>(Signature)

**Co-Principal Investigator(s) - External:** Sex ☐ Female ☐ Male<br><br>Address (provide full official address, including land phone no(s), extension no. (if any), cell phone number, and email address).<br><br>Signature or written consent of Co-PI: ___________________<br>(electronic signature or email or any sort of written consent)<br>[if more than one, please copy and paste this row for additional Co-PIs]	
**Co-Investigator(s) - Internal:** Sex ☐ Female ☒ Male<br>**Dr. Tahmeed Ahmed**<br>(Position, phone no, extension no, cell, and email address**):**<br>Senior Director, 880 2 9827001-10, Ext. 2300, email: tahmeed@icddrb.org<br><br>Signature or written consent of Co-I: (Email Confirmation)<br>(electronic signature or email or any sort of written consent)<br>[if more than one, please copy and paste this row for additional Co-Is<br><br>Do you have ethics certification? ☐ No ☒ Yes (please attach in your CV below)<br>Do you have RBM training certification? ☐ No ☐ Yes (please attach the certificate with CV below)	Primary Scientific Division of the Co-I NCSD<br><br>_____________________________<br>Approval of the Respective Senior Director/ Programme Head<br><br>(Signature)
**Co-Investigator(s) - Internal:** Sex ☐ Female ☒ Male<br>**Dr. Mustafa Mahfuz**<br>(Position, phone no, extension no, cell, and email address**):**<br>Associate Scientist, 880 2 9827001-10, Ext. 2304, email: mustafa@icddrb.org<br><br>Signature or written consent of Co-I: ___________________<br>(electronic signature or email or any sort of written consent)<br>[if more than one, please copy and paste this row for additional Co-Is<br><br>Do you have ethics certification? ☐ No ☒ Yes (please attach in your CV below)<br>Do you have RBM training certification? ☐ No ☐ Yes (please attach the certificate with CV below)	Primary Scientific Division of the Co-I NCSD<br><br>_____________________________<br>Approval of the Respective Senior Director/ Programme Head<br><br>(Signature)
**Co-Investigator(s) - Internal:** Sex ☐ Female ☒ Male<br>**Dr. Subhasish Das**<br>(Position, phone no, extension no, cell, and email address **):**<br>Assistant Scientist, 880 2 9827001-10, Ext. 2277, email:subhasish.das@icddrb.org<br><br>Signature or written consent of Co-I: ___________________<br>(electronic signature or email or any sort of written consent)<br>[if more than one, please copy and paste this row for additional Co-Is<br><br>Do you have ethics certification? ☐ No ☒ Yes (please attach in your CV below)<br>Do you have RBM training certification? ☐ No ☐ Yes (please attach the certificate	Primary Scientific Division of the Co-I NCSD<br><br>_____________________________<br>Approval of the Respective Senior Director/ Programme Head<br><br>(Signature)
**Co-Investigator(s) – External:** Sex ☐ Female ☐ Male<br><br>**Address** (provide full official address, including land phone no(s), extension no. (if any), cell phone number, and email address):<br><br>Signature or written consent of Co-I: ___________________<br>(electronic signature or email or any sort of written consent)<br>[if more than one, please copy and paste this row for additional Co-Is]	

<table>
<tr><td>Student Investigator(s) - Internal: Sex ☐ Female ☐ Male

(Position, phone no, extension no, cell, and email address):

Signature or written consent of Student Investitor: ___________________
(electronic signature or email or any sort of written consent)

Have ethics certificate? ☐ No ☐ Yes (If Yes, please attach to your CV below)</td><td>Students Affiliation

____
Approval of the Respective Senior Director/ Programme Head

(Signature)</td></tr>
<tr><td colspan="2">Student Investigator(s) - External: Sex ☐ Female ☐ Male
Address (provide full official address, including land phone no(s), extension no. (if any), cell phone number, and email address):

Signature or written consent of Student Investitor: ___________________
(electronic signature or email or any sort of written consent)</td></tr>
<tr><td colspan="2">Student Investigator(s) - External: Sex ☐ Female ☐ Male
Address (provide full official address, including land phone no(s), extension no. (if any), cell phone number, and email address):

Signature or written consent of Student Investitor: ___________________
(electronic signature or email or any sort of written consent)</td></tr>
<tr><td colspan="2">Collaborating Institute(s): Please provide full official address

Institution # 1

<table>
<tr><td>Country</td><td></td></tr>
<tr><td>Contact person</td><td></td></tr>
<tr><td>Department
(including Division, Centre, Unit)</td><td></td></tr>
<tr><td>Institution
(with official address)</td><td></td></tr>
<tr><td>Directorate
(in case of GoB i.e. DGHS)</td><td></td></tr>
<tr><td>Ministry (in case of GoB)</td><td></td></tr>
</table></td></tr>
<tr><td colspan="2">Institution # 2

<table>
<tr><td>Country</td><td></td></tr>
<tr><td>Contact person</td><td></td></tr>
<tr><td>Department
(including Division, Centre, Unit)</td><td></td></tr>
<tr><td>Institution
(with official address)</td><td></td></tr>
<tr><td>Directorate
(in case of GoB i.e. DGHS)</td><td></td></tr>
<tr><td>Ministry (in case of GoB)</td><td></td></tr>
</table></td></tr>
</table>

Institution # 3

Country	
Contact person	
Department<br>(including Division, Centre, Unit)	
Institution<br>(with official address)	
Directorate<br>(in case of GoB i.e. DGHS)	
Ministry (in case of GoB)	

Note: If less than or more than three collaborating institutions, please delete or insert blocks as needed.

Contribution by the Members of the Scientific Team:

Members' Name	Contribution								
	Research idea/ concept	Study design	Protocol writing	Respond to external reviewers' comments	Defending at IRB	Developing data collection Tool(s)	Data Collection	Data analysis/ interpretation of results	Manuscript writing
Mr. Mahfuzur Rahman	☒	☒	☒	☒	☒	☒	☒	☒	☒
Dr. Zannatun Nyma	☒	☒	☒	☒	☒	☒	☒	☒	☒
Dr. Tahmeed Ahmed	☒	☒	☐	☒	☐	☐	☐	☒	☒
Dr. Mustafa Mahfuz	☒	☒	☐	☒	☒	☐	☐	☒	☒
Dr. Subhashish Das	☒	☒	☒	☒	☒	☒	☐	☒	☒
	☐	☐	☐	☐	☐	☐	☐	☐	☐
	☐	☐	☐	☐	☐	☐	☐	☐	☐
	☐	☐	☐	☐	☐	☐	☐	☐	☐
	☐	☐	☐	☐	☐	☐	☐	☐	☐
	☐	☐	☐	☐	☐	☐	☐	☐	☐

Study Population: Sex, Age, Special Group and Ethnicity

Research Subject:
- ☒ Human
- ☐ Animal
- ☐ Microorganism
- ☐ Other (specify): ______________

Sex:
- ☐ Male
- ☒ Female
- ☐ Transgender

Age:
- ☐ 0 – 4 years
- ☐ 5 – 10 years
- ☒ 11 – 17 years
- ☐ 18 – 64 years
- ☐ 65 +

Special Group:
- ☐ Pregnant Women
- ☐ Fetuses
- ☐ Prisoners
- ☐ Destitutes
- ☐ Service Providers
- ☐ Cognitively Impaired
- ☐ CSW
- ☐ Expatriates
- ☐ Immigrants
- ☐ Refugee
- ☐ Others (specify): _________________

Ethnicity:
- ☒ No ethnic selection (Bangladeshi)
- ☐ Bangalee
- ☐ Tribal group
- ☐ Other (specify): _________________

NOTE: It is icddr.b's policy to include men, women, children and transgender in its research projects involving participation of humans, unless there is strong justification(s) for their exclusion.

Consent Process: (Check all that apply)
- ☒ Written
- ☐ Oral
- ☐ Audio
- ☐ Video
- ☐ None

Language:
- ☒ Bangla
- ☐ English
- ☐ Other (specify: ________________

Project/Study Site: (Check all that apply)

- ☐ Chakaria
- ☐ Bandarban
- ☐ Dhaka Hospital
- ☐ Kamalapur Field Site/HDSS
- ☐ Mirpur (Dhaka)
- ☐ Matlab DSS Area
- ☐ Matlab non-DSS Area
- ☐ Matlab Hospital
- ☐ Mirzapur
- ☐ Bianibazar (Sylhet)
- ☐ Kanaighat (Sylhet)
- ☐ Jakigonj (Sylhet)
- ☐ Other community in Dhaka
  Name: ___________________________________
- ☒ Other sites in Bangladesh
  Name: Rangpur
- ☐ Multi-national Study
  Name of the country_______________________

Project/Study Type: (Check all that apply)

- ☐ Case Control Study
- ☐ Clinical Trial (Hospital/Clinic/Field)*
- ☒ Community-based Trial/Intervention
- ☐ Cross Sectional Survey
- ☐ Family Follow-up Study
- ☐ Longitudinal Study (cohort or follow-up)
- ☐ Meta-analysis
- ☐ Programme Evaluation
- ☐ Programme (Umbrella Project)
- ☐ Prophylactic Trial
- ☐ Record Review
- ☐ Secondary Data Analysis
  Protocol No. of Data Source: _____________
- ☐ Surveillance/Monitoring
- ☐ Systematic Review
- ☐ Other (specify): __________________________

***Note:** International Committee of Medical Journal Editors (ICMJE) defines Clinical Trial as "*Any research project that prospectively assigns human participants to intervention and comparison groups to study the cause-and-effect relationship between a medical intervention and a health outcome*".

PI of the RRC- and ERC-approved Clinical Trials should provide necessary information to IRB Secretariat (Research Administration) for registration and uploading into relevant websites (usually at the https://register.clinicaltrials.gov/). They should also provide relevant information to the IRB Secretariat in the event of amendment/modification after their approval by RRC and ERC.
In case of a multi-country study and if a study is registered elsewhere by the prime recipient or others; it does not need to be re-registered under icddr,b's account; provided evidence of NCT registration number is submitted to the IRB.

Biological Specimen:

a) Will the biological specimen be stored for future use?	☐ Yes ☒ No ☐ Not applicable
b) If the response is 'yes', how long the specimens will be preserved?	__________ years
c) What types of tests will be carried out with the preserved specimens?	N/A
d) Will the consent be obtained from the study participants for use of the preserved specimen for other initiative(s) unrelated to this study, without their re-consent?	☐ Yes ☐ No ☒ Not applicable
e) Will the specimens be shipped to other country/ countries? If yes, name of institution(s) and country/countries.	☐ Yes ☐ No ☒ Not applicable<br>Name ____________________________
f) If shipped to another country, will the surplus/unused specimen be returned to icddr,b? If the response is 'no', then the surplus/unused specimen must be destroyed.	☐ Yes ☐ No ☒ Not applicable
g) Who will be the custodian of the specimen at icddr,b?	N/A
h) Who will be the custodian of the specimen when shipped outside Bangladesh?	N/A
i) Who will be the owner(s) of the specimens?	N/A
j) Has a MoU been signed with regards to collection, storage, use and ownership of specimen? If the response is 'yes', please attach a copy of the MoU. If the response is 'no', appropriate justification should be provided for not signing a MoU.	☐ Yes ☐ No ☒ Not applicable

Proposed Sample Size:
Sub-group (Name of subgroup e.g. Men, Women) and Number

Name	Number	Name	Number
(1) Adolescent girls	252	(3)	
(2)		(4)	
		**Total sample size**	252

Determination of Risk: Does the Research Involve (Check all that apply**)**

☐ Human exposure to radioactive agents?
☐ Foetal tissue or abortus?
☐ Investigational new device?
Specify: ___________________
☐ Existing data available from Co-investigator?
☐ Human exposure to infectious agents?
☐ Investigational new drug?
☐ Existing data available via public archives/sources?
☐ Pathological or diagnostic clinical specimen only?
☒ Observation of public behaviour?
☐ New treatment regime?

	Yes	No
Will the information be recorded in such a manner that study participants can be identified from the information directly or through identifiers linked to the study participants?	☐	☒
Does the research deal with sensitive aspects of the study participants' sexual behaviour, alcohol use or illegal conduct such as drug use?	☐	☒

Could information on study participants, if available to people outside of the research team:

	Yes	No
a) Place them at risk of criminal or civil liability?	☐	☒
b) Damage their financial standing, reputation or employability, or social rejection, or lead to stigma, divorce etc.?	☐	☒

Do you consider this research: (check one)

☐ Greater than minimal risk	☒ No more than minimal risk	☐ Only part of the diagnostic test

Note: Minimal Risk: The probability and the magnitude of the anticipated harm or discomfort to participants is not greater than those ordinarily encountered in daily life or during the performance of routine physical, psychological examinations or tests, e.g. the risk of drawing a small amount of blood from a healthy individual for research purposes is no greater than when the same is performed for routine management of patients.

**Risk Group of Infectious Agent and Use of Recombinant DNA**				
a) Will specimens containing infectious agent be collected?	☐ Yes	☒ No	☐ Not applicable	
b) Will the study involve amplification by culture of infectious agents?	☐ Yes	☒ No	☐ Not applicable	
c) If response to questions (a) and/or (b) is 'yes', to which Risk Group (RG) does the agent(s) belong? (Please visit http://shetu.icddrb.org/index.php?option=com_content&view=article&id=265&Itemid=677 to review list of microorganism by Risk Group)	☐ RG1	☐ RG2	☐ RG3	☐ RG4
d) Does the study involve experiments with recombinant DNA?	☐ Yes	☒ No	☐ Not applicable	

Does the study involve any biohazards materials/agents or microorganisms of risk group 2, 3, or 4 (GR2, GR-3 or GR4)?

☐ Yes ☒ No

[If the response is 'yes'] I, (print name of the PI) affirm that we will use the standard icddr,b laboratory procedures for biosafety of the hazardous materials/agents or microorganisms in the conduction of the study.

______________________________ ______________

Signature of the Principal Investigator **Date**

Dissemination Plan: [please explicitly describe the plans for dissemination, including how the research findings would be shared with stakeholders, identifying them if known, and the mechanism to be used; anticipated type of publication (working papers, internal (institutional) publication, international publications, international conferences/seminars/workshops/ agencies. [Check all that are applicable]

Dissemination type	Response		Description (if the response is a yes)
Seminar for icddr,b scientists/ staff	☐ No	☒ Yes	
Internal publication	☐ No	☐ Yes	
Working paper	☐ No	☐ Yes	
Sharing with GoB (e.g. DGHS/ Ministry, others)	☐ No	☐ Yes	
Sharing with national NGOs	☐ No	☐ Yes	
Presentation at national workshop/ seminar	☐ No	☒ Yes	
Presentation at international workshop/ conference	☐ No	☒ Yes	
Peer-reviewed publication	☐ No	☒ Yes	
Sharing with international agencies	☐ No	☐ Yes	
Sharing with donors	☐ No	☐ Yes	
Policy brief	☐ No	☐ Yes	
Other			
Other			

Funding:

Is the protocol fully funded?	☒ Yes	☐ No
If the answer is yes, please provide sponsor(s)'s name	1. BMGF	
	2.	
Is the protocol partially funded?	☐ Yes	☐ No
If the answer is yes, please provide sponsor(s)'s name	1.	
	2.	

If fund has not been identified:

Is the proposal being submitted for funding?	☐ Yes	☐ No
If yes, name of the funding agency	1.	
	2.	

Conflict of interest:
Do any of the participating investigators and/or member(s) of their immediate families have an equity relationship (e.g. stockholder) with the sponsor of the project or manufacturer and/or owner of the test product or device to be studied or serve as a consultant to any of the above?

☒ No ☐ Yes (please submit a written statement of disclosure to the Executive Director, icddr,b)

Proposed Budget:

Dates of Proposed Period of Support

(Day, Month, Year - DD/MM/YY)

Beginning Date : 01/07/2019

End Date : 30/09/2020

Cost Required for the Budget Period ($)

Years	Direct Cost	Indirect Cost	Total Cost
**Year-1**	43,386	0	43, 386
**Year-2**	56,527	0	56,527
**Year-3**			
**Year-4**			
**Year-5**			
**Total**	99,913	0	99, 913

Certification by the Principal Investigator:

I certify that the statements herein are true, complete and accurate to the best of my knowledge. I am aware that any false, fictitious, or fraudulent statements or claims may subject me to criminal, civil, or administrative penalties. I agree to accept the responsibility for the scientific conduct of the project and to provide the required progress reports including updating protocol information in the NAVISION if a grant is awarded as a result of this application.

I also certify that I have read icddr,b Data Policies and understand the PIs' responsibilities related to archival and sharing of research data, and will remain fully compliant to the Policies. (Note: The Data Policies can be found here: http://shetu.icddrb.org/index.php?option=com_content&view=article&id=273&Itemid=685)

Signature of PI **Date**

Approval of the Project by the Division Director of the Applicant:

The above-mentioned project has been discussed and reviewed at the Division level.

Dr. Tahmeed Ahmed
Name of the Division Director Signature Date of Approval

List of abbreviations:

BCC	Behavior Change Communication
BIRTAN	Bangladesh Institute of Research and Training in Applied Nutrition
BMI	Body Mass Index
DD	Dietary Diversity
FRA	Field Research Assistant
HDDS	Household Dietary Diversity Score
HW	Health Worker
IDDS	Individual Dietary Diversity Score
IDI	In-depth Interview
IFA	Iron-Folic acid
MUAC	Mid-Upper Arm Circumference
RF	Research Fellow
WASH	Water Sanitation and Hygiene

Table of Contents

RRC APPLICATION FORM 1
Project Summary 12
- Hypothesis to be tested: 14
- Specific Objectives: 14
- Background of the Project including Preliminary Observations: 14
- Research Design and Methods 15
- Sample Size Calculation and Outcome (Primary and Secondary) Variable(s) 20
- Data Analysis 20
- Data Safety Monitoring Plan (DSMP) 21
- Ethical Assurance for Protection of Human rights 22
- Use of Animals 23
- Collaborative Arrangements 23
- Facilities Available 23
- Literature Cited 23

Budget 25
Other Support 27
Biography of the Investigators 29
Consent Form .... **Error! Bookmark not defined.**
Check-List .... **Error! Bookmark not defined.**

☒ Check here if appendix is included

Project Summary

Principal Investigator:	Mr. Mahfuzur Rahman
Research Protocol Title:	**A Cluster Randomized Controlled Trial to Measure the Efficacy of School-Based Nutrition Education in Improving Dietary Diversity among Bangladeshi Adolescent Girls**
Proposed start date: July 1, 2019	Estimated end date: September 30, 2020

Background:

a. Burden:

After first year, adolescence (10-19 years) is the second most critical period for physical growth [1]. During this period adolescents gain up to 50% of their final adult weight and 15% of their final adult height [2]; 80% of which is achieved during the early adolescence (10-15 years) [3]. This sudden growth spurt and its close association with cognitive, emotional, behavioral and hormonal changes makes adolescence, specially the early adolescence a nutritionally vulnerable period [1,2]. Data collected from multiple countries revealed that nutrient inadequacy and inadequate dietary diversity to be the major causes of adolescent malnutrition [4,5]. Currently, two in every three women and adolescent girls of Bangladesh (aged between 10-49 years) consume inadequately diversified diet [6]. In addition, most of the adolescent girls in Bangladesh enter into the motherhood in near future. More than 67% of the adolescent girls are married [7] and more than 30% of the adolescent girls are already mother or pregnant with their first child [8]. Undernutrition in adolescents leads to poor reproductive health outcomes leading to increased preterm births, infant mortality and maternal mortality in near future [9].

b. Knowledge gap:

A recent multi-country study revealed that maternal height is a key determinant of childhood nutritional status [10]. Since maternal height cannot be increased, we have to go down the life cycle and consider increasing height of adolescent girls at the population level. Despite adolescence is being declared as the last opportunity to intervene and break the inter-generational vicious cycle of malnutrition [11,12], in low and middle-income countries the prevalence of adolescent malnutrition has remained high and Bangladesh is not an exception [13]. Inadequate dietary diversity is a factor responsible for this. Improvement of dietary diversity among adolescent girls can be accomplished by several means – providing nutritional food supplements, food fortification, behaviour change communication etc. But, to our knowledge, no studies have been conducted to measure the role of behaviour change communication in improving the dietary diversity of adolescent girls.

c. Relevance:

To improve adolescent nutrition, there have been some efforts made by the agricultural sector (Department of Agricultural Extension, Bangladesh Agricultural Research Council) and its nutrition training institutions notably the Bangladesh Institute of Research and Training in Applied Nutrition (BIRTAN) in linking school nutrition with agriculture, school gardening through the agriculture/horticulture and practical nutrition modules in the curriculum [14]. However, nutrition module in the curriculum is not compulsory for all the students and 'Krishi Shikhya' that covers agriculture or horticulture is the elective subject for the adolescent boys. Although 'Home Economics' module includes a brief discussion of balanced diet it does not cover dietary diversity focusing on 16 food groups. Moreoevr, 'Home Economics' is also an elective subject; therefore, the likelihood of receiving the messages even on the balanced diet by all the adolescent girls in the schools is uncertain. Therefore, it is very likely that a large number of adolescents girls are not receving the messages on dietary diversity from schools. Except for an intervention based on behaviour change communication, the other interventions aimed at improving the nutritional status of the adolescent girls are expensive and may not be scalable or sustainable at the program level. Keeping the above mentioned context in mind, we aim to deliver school-based nutrition education and assess its impact on dietary diversity of girls who are in their early adolescence period.

Along with that, using a qualitative approach we also aim to identify the barriers that may influence the intake of diversified food among the adolescent girls.

Hypothesis:

We hypothesise that school-based nutrition education will increase dietary diversity among the adolescent girls.

Objectives:

The objective of this study is to measure the efficacy of school-based nutrition education on dietary diversity of the adolescent girls in Bangladesh. The study will also explore the barriers and facilitators that may influence the intake of diversified food.

Methods:

We propose a matched, pair-cluster randomized controlled trial to measure the efficacy of school-based nutrition education on dietary diversity of the adolescent girls in Bangladesh. Our study will have two arms (one intervention and one control arm). After screening, based on exclusion criteria, we will prepare two separate lists (one for urban and one for rural) of schools in Rangpur district. From each list, clusters (schools) will be paired based on monthly tution fees provided by the students (as a proxy indicator of socio-economic status of the students) and infrastructure of the schools. We will randomly select one pair from each list and within each pair one school will be assigned to intervention arm and another one will be assigned to control arm through randomization. Targeting an effect size of 20 percentage point reduction of inadequate dietary diversity, a minimum of 148 adolescent girls will be required for each arm. Considering this study as a superiority trial and an effect size of 10 percentage point we also estimated sample size using the formula for sample size estimation in superiority trial. It yields the equal sample size, 148 in each arm. Therefore, it will also represent the sample size calculated based on the effect size of 20 percentage point using the formula of sample size estimation for conventional cluster randomized control trial. Eleven to fifteen years old adolescent girls studying in grade six, seven and eight will be recruited from each school in Rangpur as this division has the highest prevalence of inadequate dietary diversity, which is 72% [6]. Regions prone to natural calamities and schools already receiving any kind of intervention from other programs or projects (nutritional education, health messages, mid-day meal etc) will be excluded. To ensure household level participation and support, caregivers will be invited to the school for a discussion at the beginning of the intervention. Nutritional education will be delivered using audio-visual techniques (audio-visual presentation) once in a week for each class, for 3 months. Individual (IDDS) and household dietary diversity scores (HDDS) will be used for measuring dietary diversity at individual and household level, respectively [15]. IDDS and HDDS data will be collected at recruitment, at the end of education intervention and again after 3 months of the completion of intervention. Following national guidelines, weekly iron-folic acid (IFA) supplementation will be provided to both intervention and control arm for 3 months. For identifying the barriers to and facilitators of intake of diversified food, a qualitative research will be conducted after the intervention. Adolescent girls having improved and girls showing no improvement in individual dietary diversity score will be recruited purposively for the qualitative assessment.

Outcome measures/variables:

Primary outcome of the intervention trial will be individual level dietary diversity among the adolescent girls. As the secondary outcome of interest we will measure household dietary diversity of the adolescent girls, changes inanthropometry, and anaemia status.

Description of the Research Project

Hypothesis to be tested:

In a hypothesis testing research proposal, briefly mention the hypothesis to be tested and provide the scientific basis of the hypothesis, critically examining the observations leading to the formulation of the hypothesis.

Does this research proposal involve testing of hypothesis: ☐ No ☒ Yes (describe below)

We hypothesise that school-based nutrition education will increase the dietary diversity among the adolescent girls.

Specific Objectives:

Describe the specific objectives of the proposed study. State the specific parameters, gender aspects, biological functions, rates, and processes that will be assessed by specific methods.

- To measure the efficacy of school-based nutrition education in improving dietary diversity among Bangladeshi adolescent girls
- To explore the barriers and facilitators that may influence the intake of diversified food

Background of the Project including Preliminary Observations:

Provide scientific validity of the hypothesis based on background information of the proposed study and discuss previous works on the research topic, including information on sex, gender and diversity (ethnicity, SES) by citing specific references. Critically analyze available knowledge and discuss the questions and gaps in the knowledge that need to be filled to achieve the proposed aims. If there is no sufficient information on the subject, indicate the need to develop new knowledge.

The world's adolescent population (age of 10-19 years) is about 1200 million [16] and more than three quarters of this age group are living in developing countries [17,18]. Adolescent population of Bangladesh is about 27.7 million and among them 13.7 million are girls and 14 million are boys [19]. According to World Health Organization, children aged between 10-19 years are considered as adolescents [20]. This second decade of life has two parts: early adolescence (10-14 years) and late adolescence (15-19 years) [21]. After first year, adolescence (10-19 years) is the second most critical period for physical growth [1]. During this period, adolescents gain up to 50% of their final adult weight, 15% of their final adult height [2] and 45% of the maximal skeletal mass [22]; of this entire growth, 80% is achieved during the early adolescence (10-15 years) [3].

There are some specific reasons for which adolescence is a unique intervention period in the life cycle [23]. Firstly, after adult height is achieved, the accumulation of significant amounts of additional bone mass is implausible [24]. Secondly, peak velocity of linear growth of adolescent girls take place approximately six to twelve months prior to menarche [24]. The current mean age at menarche is 12.8 years in Bangladesh [25]. Hence, any kind of health intervention will be very worthwhile if it is given during the early adolescence. This early adolescence is recognised as a nutritionally vulnerable period because of the sudden growth spurt during adolescence and its close association with cognitive, emotional and hormonal changes [1,2]. Hence, it is essential to ensure proper and adequate nutritional support during adolescence. In Bangladesh, nutritional status of adolescent girls is not satisfactory. Stunting among adolescence is 32% in India, 36% in Bangladesh, and 47% in Nepal and low BMI is 53% in India, 50% in Bangladesh, 36% in Nepal [26]. Study conducted among 12-19 years old post-menarcheal adolescent girls in rural Bangladesh found undernutrition is widespread in this population, with nearly half of the adolescents being stunted and more than 40% underweight [27]. Another study found that, a large number of adolescent girls suffer from various degrees of nutritional disorders [28,29].

Most hazardous impacts of adolescent malnutrition are reduced productivity, poor school performance and adverse reproductive outcomes [30]. A significant association was found between height of the mother and stunting status of the adolescent son or daughter [31]. Results from a multi-country study showed maternal height are a key determinant of childhood nutritional status [10]. Since maternal height cannot be increased,

we have to go down the life-cycle and consider increasing height of adolescent girls at the population level. It would be the last opportunity to intervene and break the inter-generational vicious cycle of malnutrition [11, 12].

Many studies conducted in both developed and developing countries revealed that nutrient adequacy is strongly associated with dietary diversity [32,33]. Diet containing diverse food items provides wide range of macro and micronutrients and enhances nutritional quality of diet [34]. In contrary, monotonous diets based on starchy staples lack essential nutrients and contribute to the burden of malnutrition [4,35]. Dietary diversity among women of Bangladesh is very low. At the national level, two-thirds (66%) of women consumed inadequately diversified diet [6]. One in every three women and adolescent girls of Bangladesh aged 10-49 years consumes diets with inadequate diversity which was found to be highest in Sylhet and Rangpur division (72%) [6]. Previous study showed a large proportion of the participants of that study consumed meat (62.5%), fish (53.8%) and eggs (58.4%) 3 to 4 times or less in the week preceding the interview. A substantial proportion of the girls did not take milk (46.1%) and liver (64.6%) at all in the week. About 27.7% did not take leafy vegetables; while substantial proportions of the participants had other vegetables (72.2%) 3 to 4 times or more in the week [36].

Lack of knowledge of selecting proper food items increases the burden of malnutrition among adolescent girls [36]. Health and nutrition knowledge and healthy habits of female adolescents have crucial roles in maintaining future family health and nutrition [13]. However, except for an intervention based on behaviour change communication (BCC), the other interventions are expensive and may not be scalable or sustainable at program level. Moreover, there is limited data regarding nutritional status of Bangladeshi adolescent girls who are at their early adolescence. There is no study which has ever assessed the role of nutritional education in improving dietary diversity of adolescent girls of Bangladesh. Keeping this context in mind, we aim to deliver school-based nutrition education and assess its impact on dietary diversity of girls who are in their early adolescent period.

Study conducted among participants more than or equal to eighteen years of age (55% women) in Switzerland revealed that among the factors of healthy eating, evidence points to food price (e.g., healthy foods are too expensive) [37,38], food taste (e.g., healthy foods lack taste) [38, 39], time constrains (e.g., lack of time to prepare and cook heathy foods) [38,39] and lack of willingness [40] – these factors are impediments to maintain healthy eating behaviour [41]. Another study conducted among the fifth-grade children and adult caregivers identified overarching facilitators of consuming several food groups included emphasizing health benefits increasing availability of and accessibility to foods, affordability and guidance on food preparation [42]. A study conducted in rural Bangladesh among 15-49 years old women identified numerous food security determinants including land tenure, use of vegetable gardens, income generation, women's empowerment, access of market for women, access to media and literacy [43]. However, in case of dietary diversity, they found only literacy to be associated with improved dietary diversity [43]. To our knowledge, there is no study which has ever identified these barriers and facilitators which might influence dietary diversity. Considering mentioned perspective, along with the aim to deliver nutrition education and assess its impact on dietary diversity, we also aim to identify the barriers and facilitators that may influence the intake of diversified food among adolescent girls.

Research Design and Methods

Describe the research design and methods and procedures to be used in achieving the specific aims of the research project. If applicable, mention the type of personal protective equipment (PPE), use of aerosol confinement, and the need for the use BSL2 or BSL3 laboratory for different part of the intended research in the methods.. Define the study population with inclusion and exclusion criteria, the sampling design, list the important outcome and exposure variables, describe the data collection methods/tools, and include any follow-up plans if applicable. Justify the scientific validity of the methodological approach (biomedical, social, gender, or environmental).

Also, discuss the limitations and difficulties of the proposed procedures and sufficiently justify the use of them.

We propose a matched, pair-cluster randomized controlled trial to measure the efficacy of school-based nutrition education in improving dietary diversity among Bangladeshi adolescent girls. After screeing, based on exclusion criteria (schools having any programme such as nutrition education, health message, mid-day meal), we will prepare two separate lists (one for urban and one for rural) of schools in Rangpur district.

From each list, clusters (schools) will be paired based on monthly tution fees provided by the students (as a proxy indicator of socio-economic status of the students) and infrastructure of the schools. We will randomly select one pair from each list and within each pair one school will be assigned to intervention arm and another one will be assigned to control arm through randomization. Randomization will be done by computer-generated random numbers using STATA. Eleven to fifteen years old adolescent girls studying in grade six, seven and eight will be recruited from four selected schools (2 urban and 2 rural schools). Nutrition education will be given in intervention arm and information on water, sanitation and hygiene (WASH) and iron-folic acid will be provided in both the arms. Although we will provide intervention to all the students in each grade, we will select an equal number of gilrs from each grade to assess the outcomes of intervention. We will also conduct surveys at the households of those selected girls to capture data on household level dietary diversity, water, sanitation and hygiene, and socio-economic status. Three surveys will be conducted among the same adolescent girls and at their household level at three different time points-before rolling out of the intervention, after accompolishment of the intervention and after three months of completion of intervention. The required sample size for survey has been mentioned under the section of sample size calculation. At the end of intervention, in-depth interviewes with some purposively selected adolescent girls will be conducted to explore the barriers and facilitators which may influence the intake of diversified food among the adolescent girls.

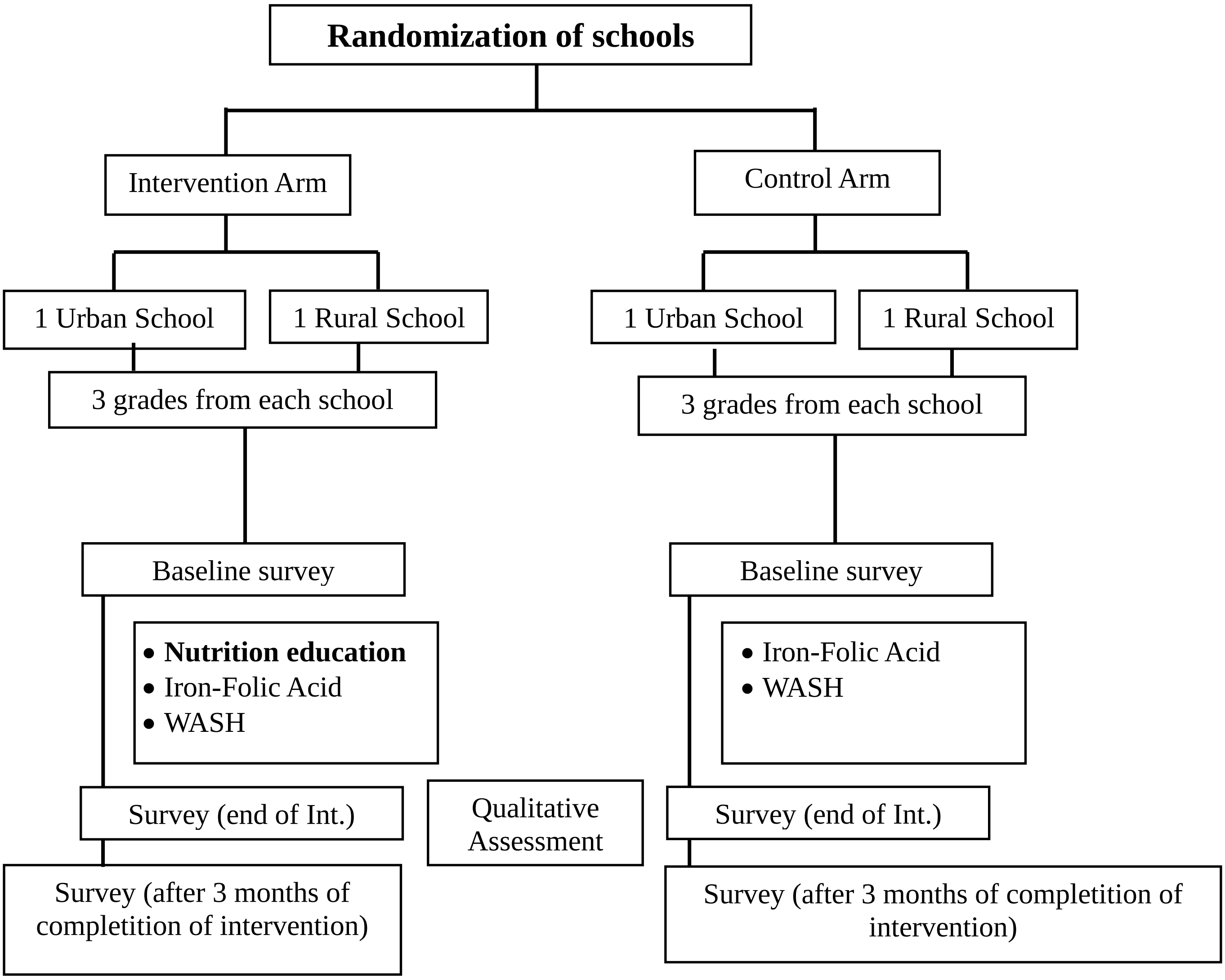

Figure 1: Study Design

Intervention component:

At the beginning of the intervention, we will arrange a parents' meeting at the schools. In parents' meeting, we will discuss about the objective of the study, overview of dietary diversity, health benefits of dietary diversity (DD) on adolescent health and parent's role in ensuring intake of diversified food of adolescent girls. An hour-long nutrition education session will be conducted to each class/grade of the schools under intervention arm. Eight such educational discussion sessions will be held in first 2 months and during month three the previous eight education sessions will be repeated. Nutrition education session will be delivered using audio-visual techniques (audio-visual presentation). In addition, we will arrange quizes to inspire them and to reduce monotony of the sessions. We will provide pamphlets for their remembrance. Components of eight educational sessions will include overview of 16 food groups of individual dietary diversity score (IDDS) chart, detail description of 16 food groups presenting dietary diversity, overview of basic food components, importance of consuming diversified foods, strategies to select a diversified meal and remove monotony in food, proper timing and frequency of taking meals during 24 hours, selecting diversified meal in low expense, consequences of absence or scarcity of dietary diversity in meal (Different food items under 16 food groups are given in Appendix-4).

Following national guideline, weekly iron-folic acid (IFA) supplementation will be provided to both intervention and control arm for 3 months. We will provide capsule Ferocit Z (provided by government of Bangladesh in different government medical college and healthcare facilities) as Iron-folic acid (IFA) supplementation. It contains ferrous sulphate - 150Mg, folic acid - 0.5Mg and zinc sulphate monohydrate - 61.8Mg. Iron-folic acid (IFA) supplementation will be provided among the girls of each class/grade (six, seven and eight) of all selected schools regardless of intervention and control arm of the study. In the intervention arm, apart from nutrition education the adolescent girls will also receive messages of water, sanitation and hygiene (WASH). But in control arm, the girls will receive messages on WASH only (along with IFA). The meassage on WASH will include safe source of drinking water, improved toilet faciltity and how to maintain good sanitation and five critical times of handwashing.

Description of field site:

The study will be carried out in 2 urban and 2 rural schools of Rangpur district. Rangpur is situated in the northern part of Bangladesh. Rangpur has been selected as the study site because prevalence of inadequate dietary diversity among adolescent girls is higher (72%) in Rangpur [6]. In addition, Rangpur is one of the areas where adolescent girls with low wealth are food insecure and are more likely to have inadequate dietary diversity, particularly during the post-aus season [44]. Regions which are prone to natural calamities and schools already receiving any kind of interventions (nutritional education, health messages etc.) from other programs or projects will be excluded.

Selection of study participants:

Since the intervention will be given all adolescent girls, the following individual level inclusion and exclusion criteria are for evaluation of process and outcomes only.

a. Inclusion criteria: All of the following criteria will be met for a participant to be enrolled in the study-
    - Never married adolescent school girls studying in grade six, seven and eight (age range 11-15) at the selected schools
    - Household(s) belong to selected study participants
    - Girl(s) willing to give assent
b. Exclusion criteria: Adolescent girls will be excluded if they have the following criteria-
    - Adolescent girls who are < 11 years and > 15 years
    - Presence of any kind of chronic disease among study participants
    - Presence of any major psychiatric illness

Enrolment, Screening and Consenting:

Screening and enrollment of the study participants will be done at the study site (selected urban and rural schools of Rangpur). Trained research staff of icddr,b will explain the study in detail, answer any question from the parents, school teachers and adolescent girls.

Since our intervention will be school-based nutrition education, we will obtain initial approval from the school authority and headmaster of the school. If the legal guardians (school headmasters) and adolescent girls are interested to volunteer in the study, the designated staffs will proceed to screening and consenting. Screening will be done based on the inclusion and exclusion criteria. If the adolescent girl is eligible to participate, the next step will be taking consent. As our study participants are <18 years, we will take assent from the adolescent girls and we will also take consent from the mother or caregiver of the adolescent girls before including them in this study. If the mother or caregiver of an adolescent girl refuses to give consent, we will not include that adolescent girl in our study. Prior to signing the consent form, they will have an opportunity to ask any question about the study.

Data collection:

Anthropometry data:

Weight of the study participants will be measured by portable electronic weighting scale (TANITA Corporation Japan) and height will be measured by Seca stadiometer. Weight and height will be used for computing BMI and height-for-age z-score. BMI below 18.5 is referred to as underweight, 18.5-24.9 as normal range, 25.0-29.9 as overweight and 30.0 and above as obese [45]. Height-for-age z-score <-2SD will be considered as stunted [13].

Mid upper-arm circumference (MUAC) will be measured at the midpoint of the left upper arm (extended with the palm facing inwards) between the acromion process and the tip of the olecranon, using a plastic non-stretchable tape to the nearest millimetre [46].

Under supervision of research fellow (RF), trained field research assistants (FRA) will take all the measurements and trained health workers (HW) will help in keeping records. All the anthropometry will be measured at recruitment, at the end of the intervention and again after 3 months of the completion of intervention.

Socio-economic and morbidity data:

Socio-economic and morbidity data of adolescent school girls will be collected at recruitment, at the end of education intervention and again after 3 months of the completion of intervention by using pretested, semi-structured questionnaire. General morbidity symptoms they have experienced in the previous one week will be recorded. The morbidity data will include fever, cough/cold, diarrhoea/dysentery, stomach ache, respiratory problems, ear and eye problems, skin problems and others. A complete menstrual history will be taken during morbidity related data collection.

Dietary diversity data:

Dietary diversity data will be collected at individual as well as at household level. Data will be collected using 24-hour recall dietary diversity questionnaire. Individual level data will be collected from the adolescent girls and household level data will be collected from the mothers or caregivers of the selected adolescent girls. Individual dietary diversity score (IDDS) and household dietary diversity score (HDDS) will be used for measuring dietary diversity at individual and household level respectively [15]. Our questionnaire regarding dietary diversity will include 16 food groups: cereals; white roots and tubers; vitamin A rich vegetables and tubers; dark green leafy vegetables; other vegetables; vitamin A rich fruits; other fruits; organ meat; flesh meats; eggs; fish and seafood; legumes; nuts and seeds; milk and milk products; oils and fats; sweets; spices, condiments and beverages [15]. The 16 food groups included in dietary diversity questionnaire will be aggregated into 12 food groups and 9 food groups to create household dietary diversity score (HDDS) and individual dietary diversity score (IDDS) respectively [15]. IDDS and HDDS data will be collected at recruitment, at the end of education intervention and again after 3 months of the completion of intervention.

Estimation of haemoglobin level:

Haemoglobin status will be measured from a finger prick blood sample using a HemoCue machine (Hb 301, HemoCue AB, Angelholm, Sweden). We will take the second drop of blood from the study participants as sample. Haemoglobin level will be estimated at recruitment, at the end of educational intervention and again after 3 months of the completion of intervention.

Qualitative data:

To explore the barriers to and facilitators of intake of diversified food among the adolescent girls, in-depth interviews will be conducted among the purposively selected girls. Twelve IDIs will be conducted (3 adolescent girls having improved and 3 adolescent girls showing no improvement in dietary diversity score; from each intervention school). Although we have assumed the estimated number (12) of IDIs will provide us with sufficient information, number of IDIs will depend on data saturation. Keeping the research objective in mind we will develop a flexible guideline (appendix-3) to conduct the interview. It will take about one hour to conduct an in-depth interview.

Sample Size Calculation and Outcome (Primary and Secondary) Variable(s)

Clearly mention your assumptions. List the power and precision desired. Describe the optimal conditions to attain the sample size. Justify the sample size that is deemed sufficient to achieve the specific aims.

The "State of Food Security and Nutrition in Bangladesh, 2015" reports the nationwide prevalence of inadequate dietary diversity among 10-49 years old women to be 66% [6]. Considering this prevalence, we assume that our intervention would reduce 20 percentage points. Based on the assumption and following conditions below, our estimated sample size will be as follows:

P_1= Test value of the population proportion (inadequate dietary diversity) which is 66

P_2 = Anticipated proportion in the intervention group which is 46

$z_{\alpha/2}$ = 1.96 (from z table for type 1 error of 5%)

z_{β} = 0.842 (from z table for 80% power)

n = required sample size

$$= \frac{P_1 (1 - P_1) + P_2 (1 - P_2)}{(P_1 - P_2)^2} \times (Z_{\alpha/2} + Z_{\beta})^2$$

= 93 in each arm

However, considering this study as a superiority trial and an effect size of 10 percentage point we also estimated sample size using the formula for sample size estimation in superiority trial. It yields the equal sample size, 93 in each arm.

π_1= Test value of the population proportion (inadequate dietary diversity) which is 66%

π_2 = Anticipated proportion in the intervention group which is 56%

n = required sample size

$$= \frac{(Z_{\alpha/2} + Z_{\beta})^2 \left[\pi_1 (1 - \pi_1) + \pi_2 (1 - \pi_2)\right]}{(\pi_1 - \pi_2 - \delta)^2}$$

It will also represent the sample size calculated based on the effect size of 20 percentage point using the formula of sample size estimation for conventional cluster randomized control trial. After considering design effect 1.5 and 5% attrition rate, desired sample size will be 148 in each arm. Since we will have two schools in each arm, we will collect data of 74 girls from each school. Thus, to reach the desired sample size we will collect data of 25 girls from each grade in a school.

Data Analysis

Describe plans for data analysis, including stratification by sex, gender and diversity. Indicate whether data will be analysed by the investigators themselves or by other professionals. Specify what statistical software packages will be used and if the study is blinded, when the code will be opened. For clinical trials, indicate if interim data analysis will be required to determine further course of the study.

Data entry will be done by using Microsoft access and Data analysis will be done by STATA (version 14) statistical software program.

Quantitative data analysis:

Dietary diversity scores will be calculated by summing up the number of food groups consumed by the individual participant over the last 24 hours. There is no established cut off point in terms of food groups to indicate adequate or inadequate dietary diversity for the IDDS and HDDS. So, it is recommended to use the mean score for analytical purpose [15]. Dietary diversity scores have been validated for several age/sex groups including non-breast-fed children [47,48], adolescents [5] and adults [34,49]. Categorical variables

will be presented as frequency and percentage. Continuous variables will be presented as mean with standard deviation. To see the relationship with study group, t-test and Mann-Whitney test (skewed data) will be used. Linear mixed effect model- a longitudinal data analysis technique will be used to measure the role of intervention on individual dietary diversity and household level dietary diversity. Intention-to-treat and per protocol analysis will be done to assess the effect of nutritional education on changes in the mean dietary diversity scores among the participants.

We will perform pairwise comparison tests to see whether the baseline survey data are comparable between intervention and control groups. If the baseline survey reports are found to be not comparable, we will adjust the variables using difference-in-differences analysis.

Qualitative data:

The transcripts of the interviews will be read thoroughly for familiarity with the data. Furthermore, the field impression notes or memos will be sent to the investigator by the interviewers in order to get feedback on what issues they need to investigate more in-depth. The investigator will provide feedback on those immediately so that the interviewers can investigate more in-depth on those issues from the next interviews. In this iterative way, initial analysis will begin during data collection. After data collection, recorded in-depth interviews will be transcribed and read thoroughly for familiarity with the data. Team members will code these data from transcripts based on apriori and inductive code. Apriori code will be based on topic guide considering existing literature. Coded transcripts will be exchanged to check consistency. Common patterns will be identified from coding and data will be analyzed thematically.

Data Safety Monitoring Plan (DSMP)

All clinical investigations (research protocols testing biomedical and/or behavioural intervention(s)) should include the Data and Safety Monitoring Plan (DSMP). The purpose of DSMP is to provide a framework for appropriate oversight and monitoring of the conduct of clinical trials to ensure the safety of participants and the validity and integrity of the data. It involves involvement of all investigators in periodic assessments of data quality and timeliness, participant recruitment, accrual and retention, participant risk versus benefit, performance of trial sites, and other factors that can affect study outcome.

1. Study data collection and administrative forms will be identified by coded number to maintain participants' confidentiality and to enable tracking throughout the study.

2. All information regarding study subjects will be kept in password-protected computer files or in locked file cabinets that can be accessed only by authorized study personnel. Chart information and information from study records will not be released without written permission from the participant's legal guardian (school headmaster). These records will be kept in locked file cabinets. However, records may be reviewed by representatives from the Research Review Committee and Ethical Review Committee of icddr,b.

3. All study related documents will be kept in locked cabinets in locked rooms with limited access. Information in the electronic database established at icddr,b will be password protected and access will be available only to authorized research team members, any information printed from the database will be stored in locked files until its use is complete and then shredded.

4. The study investigators will be responsible for ensuring complete and accurate documentation for the study and for each subject including records detailing each participant's progress through the study, signed informed consent forms, correspondence with IRB(s), adverse event reports and information regarding participant's discontinuation and completion of the study.

5. Data entry and cleaning will be done at icddr,b.

Ethical Assurance for Protection of Human rights

Describe the justifications for conducting this research in human participants. If the study needs observations on sick individuals, provide sufficient reasons for using them. Indicate how participants' rights will be protected, and if there would be benefit or risk to each participants of the study. Discuss the ethical issues related to biomedical and social research for employing special procedures, such as invasive procedures in sick children, use of isotopes or any other hazardous materials, or social questionnaires relating to individual privacy. Discuss procedures safeguarding participants from injuries resulting from study procedures and/or interventions, whether physical, financial or social in nature. [Please see Guidelines]

Justification for conducting this research in human subject:

We will provide nutritional education as intervention to improve dietary diversity among Bangladeshi adolescent school girls aged 11-15 years. To do so, recruitment of human subject (adolescent girls) is a must. Therefore, we propose this study involving human participants (adolescent school girls).

Steps to ensure participant's right:

Each participant will be treated according to what is morally right and proper. Fair subject selection will be done to ensure validity and reliability. Participants will be recruited after proper screening and taking consent as well as assent. Autonomy and justice will be ensured by-

- Taking well-informed consent from the legal guardian of the participant (We will take approval from the headmaster of the schools. Moreover, we will organize a parents' meeting at the school before rolling out the intervention. We will take consent from them on that day.)
- Taking assent from participant (our study participants will be 11-15 years old adolescent girls. As they are below 18 years, so they are not eligible for providing consent. This is why, we will take assent from them)
- Ensuring privacy and confidentiality
- Ensuring right to withdrawal at any stage
- Delivering adequate information in an understandable, clear, unambiguous, effective manner and in non-technical language
- Negating coercion, undue induction, unduly influence or intimidation

Study benefits:

We will provide intervention (nutritional education) to all the students of intervention arm. Additionally, all adolescent girls (both in intervention and in control arm) will receive weekly Iron-folic acid (IFA) supplementation for 3 months following national guideline and information on water, sanitation and hygine (WASH).

Significant risks / adverse events:

Our intervention (nutrition education) is not related to any health hazard of study participants. Haemoglobin will be measured from a finger prick sample using a HemoCue machine (Hb 301, HemoCue AB, Angelholm, Sweden) and trained staff will be recruited to take blood samples from the adolescent girls. So, we do not envision any significant risks related to participation in this study.

Ethical issues related to social or biomedical research:

Our research project does not include any steps which may result stigmatization or social exclusion of any of the participant.

Procedures safeguarding participants from risks resulting from study procedures and/or interventions:

The principle investigator and the team are obliged to secure the wellbeing and beneficence of the study participants.

Use of Animals

Describe if and the type and species of animals to be used in the study. Justify with reasons the use of particular animal species in the research and the compliance of the animal ethical guidelines for conducting the proposed procedures.

N/A

Collaborative Arrangements

Describe if this study involves any scientific, administrative, fiscal, or programmatic arrangements with other national or international organizations or individuals. Indicate the nature and extent of collaboration and include a letter of agreement between the applicant or his/her organization and the collaborating organization.

N/A

Facilities Available

Describe the availability of physical facilities at site of conduction of the study. If applicable, describe the use of Biosafety Level 2 and/or 3 laboratory facilities. For clinical and laboratory-based studies, indicate the provision of hospital and other types of adequate patient care and laboratory support services. Identify the laboratory facilities and major equipment that will be required for the study. For field studies, describe the field area including its size, population, and means of communications plus field management plans specifying gender considerations for community and for research team members.

We will set up a field office in an urban area of Rangpur where logistics will be temporarily stored. Projectors, laptops, sound systems, iron-folic acid supplementation will be carried from field office to schools by health workers. Staff members of the study will use rickshaw or autorickshaw to go from filed office to schools. Iron-folic acid (IFA) supplements will also be carried by health workers. As our study participants are adolescent school girls, we will recruit female staffs as field research assistants (FRA) and health workers (HW) as they will be involved in taking face-to-face interview of adolescent girls, measuring anthropometry, provision of iron-folic acid (IFA) to the adolescent girls.

Literature Cited

Identify all cited references to published literature in the text by number in parentheses. List all cited references sequentially as they appear in the text. For unpublished references, provide complete information in the text and do not include them in the list of Literature Cited. There is no page limit for this section, however, exercise judgment in assessing the "standard" length.

1. World Health Organization. Adolescent nutrition: a review of the situation in selected South-East Asian countries. 2006.
2. Spear BA. Adolescent growth and development. Journal of the Academy of Nutrition and Dietetics. 2002:S23.
3. Srikantia SG. Pattern of growth and development of Indian girls and body size of adult Indian women. Women and Nutrition in India. 1989;5:108-52.
4. Vakili M, Abedi P, Sharifi M, Hosseini M. Dietary diversity and its related factors among adolescents: a survey in Ahvaz-Iran. Global Journal of Health Science. 2013 Mar;5(2):181.
5. Mirmiran P, Azadbakht L, Esmaillzadeh A, Azizi F. Dietary diversity score in adolescents-a good indicator of the nutritional adequacy of diets: Tehran lipid and glucose study. Asia Pacific Journal of Clinical Nutrition. 2004 Jan 1;13(1):56-60.
6. James P Grant School of Public Health and National Nutrition Services. (2016). State of food security and nutrition in Bangladesh 2015. Dhaka, Bangladesh: James P Grant School of Public Health and National Nutrition Services.
7. Akhter N, Sondhya FY. Nutritional status of adolescents in Bangladesh: Comparison of severe thinness status of a low–income family's adolescents between urban and rural Bangladesh. Journal of education and health promotion. 2013;2.
8. Bangladesh Demographic Health Survey, 2014
9. Nithya DJ, Bhavani RV. Dietary diversity and its relationship with nutritional status among adolescents and adults in rural India. Journal of biosocial science. 2018 May;50(3):397-413.

10. MAL-ED Network Investigators. Childhood stunting in relation to the pre-and postnatal environment during the first 2 years of life: The MAL-ED longitudinal birth cohort study. PLoS medicine. 2017 Oct 25;14(10):e1002408.
11. Golden MH. Is complete catch-up possible for stunted malnourished children? European Journal of Clinical Nutrition. 1994 Feb 1;48(1):58-71.
12. Martorell R, Khan LK, Schroeder DG. Reversibility of stunting: epidemiological findings in children from developing countries. European Journal of Clinical Nutrition. 1994 Feb; 48: S45-57.
13. Alam N, Roy SK, Ahmed T, Ahmed AS. Nutritional status, dietary intake, and relevant knowledge of adolescent girls in rural Bangladesh. Journal of Health, Population, and Nutrition. 2010 Feb;28(1):86.
14. File///C/Users/Lalita/AppData/Local/Microsoft/Windows/Netcache/IE/AQCX0CDR/Nutrition Thematics%20Study.pdf
15. Kennedy G, Ballard T, Dop MC. Guidelines for measuring household and individual dietary diversity. Food and Agriculture Organization of the United Nations; 2011.
16. Kotecha PV, Patel S, Baxi RK, Mazumdar VS, Misra S, Modi E, Diwanji M. Reproductive health awareness among rural school going adolescents of Vadodara district. Indian journal of sexually transmitted diseases. 2009 Jul;30(2):94.
17. Huebler F: International Education Statistics: Global Population of Primary School Age, 2000–2015. 2008. http://huebler. blogspot.com/2008/03/global-populationof-primary-school-age.html (accessed June 5, 2014).
18. Ochola S, Masibo PK. Dietary intake of schoolchildren and adolescents in developing countries. Annals of Nutrition and Metabolism. 2014;64(Suppl. 2):24-40.
19. https://www.unicef.org/bangladesh/children_356.htm
20. http://apps.who.int/adolescent/second-decade/section2/page1/recognizing-adolescence.html
21. https://www.unicef.org/sowc2011/pdfs/Early-and-late-adolescence.pdf
22. Spear BA. Adolescent growth and development. Journal of the Academy of Nutrition and Dietetics. 2002 Mar 1:S23.
23. Public health at a glance—adolescent nutrition 2003. (http://web.worldbank.org/WBSITE/EXTERNAL/ TOPICS/EXTHEALTHNUTRITIONANDPOPULATION, accessed on 22 June 2008)
24. Stang J, Story M. Adolescent growth and development. Guidelines for adolescent nutrition services. 2005;1(6).
25. Rah JH, Shamim AA, Arju UT, Labrique AB, Rashid M, Christian P. Age of onset, nutritional determinants, and seasonal variations in menarche in rural Bangladesh. Journal of health, population, and nutrition. 2009 Dec;27(6):802.
26. Kurz KM. Adolescent nutritional status in developing countries. Proceedings of the Nutrition Society. 1996 Mar;55(1B):319-31.
27. Rah JH, Christian P, Shamim AA, Arju UT, Labrique AB, Rashid M. Predictors of stunting and thinness in post-menarcheal adolescent girls in rural Bangladesh. Public health nutrition. 2009 Dec;12(12):2400-9.
28. Ahmed F, Khan MR, Islam M, Kabir I, Fuchs GJ. Anaemia and iron deficiency among adolescent schoolgirls in peri-urban Bangladesh. European journal of clinical nutrition. 2000 Sep;54(9):678.
29. Ahmed F, Khan MR, Banu CP, Qazi MR, Akhtaruzzaman M. The coexistence of other micronutrient deficiencies in anaemic adolescent schoolgirls in rural Bangladesh. European journal of clinical nutrition. 2008 Mar;62(3):365.
30. Partnership for Child Development (1998) The anthropometric status of schoolchildren in five countries in the Partnership for Child Development. Proc Nutr Soc 57, 149–158.
31. Bosch AM, Baqui AH, van Ginneken JK. Early-life determinants of stunted adolescent girls and boys in Matlab, Bangladesh. Journal of health, population, and nutrition. 2008 Jun;26(2):189.
32. Torheim LE, Ouattara F, Diarra MM, Thiam FD, Barikmo I, Hatloy A, Oshaug A. Nutrient adequacy and dietary diversity in rural Mali: association and determinants. Eur J Clin Nutr. 2004;58:594–604.

33. Roche ML, Creed-Kanashiro HM, Tuesta I, Kuhnlein HV. Traditional food diversity predicts dietary quality for the Awajun in the Peruvian Amazon. Public Health Nutr. 2008;11:457–65.
34. Foote JA, Murphy SP, Wilkens LR, Basiotis PP, Carlson A. Dietary variety increases the probability of nutrient adequacy among adults. The Journal of nutrition. 2004 Jul 1;134(7):1779-85.
35. Allen LH. To what extent can food-based approaches improve micronutrient status?. Asia Pacific Journal of Clinical Nutrition. 2008 Jan 1;17(S1):103-5.
36. Kabir Y, Shahjalal HM, Saleh F, Obaid W. Dietary pattern, nutritional status, anaemia and anaemia-related knowledge in urban adolescent college girls of Bangladesh. JPMA. The Journal of the Pakistan Medical Association. 2010 Aug 1;60(8):633.
37. Inglis V, Ball K, Crawford D. Why do women of low socioeconomic status have poorer dietary behaviours than women of higher socioeconomic status? A qualitative exploration. Appetite. 2005 Dec 1;45(3):334-43.
38. Nicklas TA, Jahns L, Bogle ML, Chester DN, Giovanni M, Klurfeld DM, Laugero K, Liu Y, Lopez S, Tucker KL. Barriers and facilitators for consumer adherence to the dietary guidelines for Americans: the HEALTH study. Journal of the Academy of Nutrition and Dietetics. 2013 Oct 1;113(10):1317-31.
39. Dijkstra SC, Neter JE, van Stralen MM, Knol DL, Brouwer IA, Huisman M, Visser M. The role of perceived barriers in explaining socio-economic status differences in adherence to the fruit, vegetable and fish guidelines in older adults: a mediation study. Public health nutrition. 2015 Apr;18(5):797-808.
40. Kearney JM, McElhone S. Perceived barriers in trying to eat healthier–results of a pan-EU consumer attitudinal survey. British Journal of Nutrition. 1999 Jun;81(S1):S133-7.
41. de Mestral C, Khalatbari-Soltani S, Stringhini S, Marques-Vidal P. Fifteen-year trends in the prevalence of barriers to healthy eating in a high-income country–3. The American journal of clinical nutrition. 2017 Jan 25;105(3):660-8.
42. Nicklas TA, Jahns L, Bogle ML, Chester DN, Giovanni M, Klurfeld DM, Laugero K, Liu Y, Lopez S, Tucker KL. Barriers and facilitators for consumer adherence to the dietary guidelines for Americans: the HEALTH study. Journal of the Academy of Nutrition and Dietetics. 2013 Oct 1;113(10):1317-31
43. Harris-Fry H, Azad K, Kuddus A, Shaha S, Nahar B, Hossen M, Younes L, Costello A, Fottrell E. Socio-economic determinants of household food security and women's dietary diversity in rural Bangladesh: a cross-sectional study. Journal of Health, Population and Nutrition. 2015 Dec;33(1):2.
44. Adolescent Nutrition in Bangladesh. Global Alliance for Improved Nutrition. June 2018. https://www.gainhealth.org/wp-content/uploads/2018/09/Adolescent-nutrition-in-Bangladesh-June-2018.pdf. Accessed on 10 July 2019.
45. Akhter N, Sondhya FY. Nutritional status of adolescents in Bangladesh: Comparison of severe thinness status of a low–income family's adolescents between urban and rural Bangladesh. Journal of education and health promotion. 2013;2.
46. Gibson RS. Principles of nutritional assessment. Oxford university press, USA; 2005
47. Steyn NP, Nel JH, Nantel G, Kennedy G, Labadarios D. Food variety and dietary diversity scores in children: are they good indicators of dietary adequacy? Public Health Nutrition. 2006 Aug;9(5):644-50.
48. Kennedy GL, Pedro MR, Seghieri C, Nantel G, Brouwer I. Dietary diversity score is a useful indicator of micronutrient intake in non-breast-feeding Filipino children. The Journal of Nutrition. 2007 Feb 1;137(2):472-7.
49. Arimond M, Wiesmann D, Becquey E, Carriquiry A, Daniels MC, Deitchler M, Fanou Fogny N, Joseph ML, Kennedy G, Martin-Prevel Y, Torheim LE. Simple Food Group Diversity Indicators Predict Micronutrient Adequacy of Women's Diets in 5 Diverse, Resource-Poor Settings–. The Journal of Nutrition. 2010 Sep 29;140(11):2059S-69S.

Review of External Reviewer

Reviewer 1
Reviewer's Name: Lalita Iyer Bhattachjee
Reviewer's details:
Meeting the Undernutrition Challenge –MUCH
Food and Agriculture Organization of the United Nations
Bangladesh
skype: lalita.bhattacharjee
Tel: (8801) 720 189 498
Email: lalita.bhattacharjee@fao.org

Reviewer comment: Page # 11, paragraph 1, item a, Burden
It would be relevant to include a point on pregnancy in adolescents and in young mothers. Given that the adolescent fertility rate is the highest in South Asia and Bangladesh is no exception, approximately around a third or so of adolescents become mothers and must bear the burden of pregnancy at a young age that impacts birth outcomes and child nutrition.

Response: Thank you for your valuable comments. We have incorporated the burden of pregnancy at young age that impacts birth outcomes.

Reviewer comment: Paragraph 2, line # 3: Despite adolescence….It should read as 'being' rather than 'be"
Response: Thank you for your comment. We have made the changes accordingly in the protocol.

Reviewer comment: Paragraph 3, item c, Relevance: There is need to review some of the efforts made by the agricultural sector (DAE, BARC) and its nutrition training institutions notably the Bangladesh Institute of Research and Training in Applied Nutrition (BIRTAN) in linking school nutrition with agriculture, school gardening through the agriculture/horticulture and practical nutrition modules in the curriculum. There have also been some initiatives under the purview of the MDGf (2009-2012) and MoFLS and MoHFW through an FAO/UNICEF/USAID supported three-year programme in Southern Bangladesh (2013-2016).
Response: Thanks. As you suggested we have incorporated some other efforts of improving adolescent nutrition under the item-Relevance.

Reviewer comment: Hypothesis: Rather than use IDDS, it is advisable to use minimum dietary diversity for women (MDDW) than also inform us about the micronutrient adequacy of diets among adoelscents and women (14-49 years). HDDS mainly provides an assessment of the access to a diversity of foods in the household it would terefore provide more of the context in terms of household access, which is a measure of affordability and purchasing power of food rather food consumption and utilization.

Response: Thank you for your comments. One of the major pitfall of using minimum dietary diversity for women (MDDW) is that it is a population-level indicator based on a recall period of a single day and night, so although data are collected from individual women, the indicator cannot be used to describe diet quality for an individual woman. This is because of normal day-to-day variability in individual intakes. We agree with you that household dietary diversity score(HDDS) provides an assessment of the access to a diversity of foods in the households and our one of the secondary objectives is to assess the access to a diversity of foods in the household by using HDDS.

Reviewer comment: Page # 15, item Study design: Under nutriton education, please include practical science – based activities such as healthy cooking demonstrations and food preparations to enhance nutrition knowledge and sensitize change.

Response: Thank you for your valuable comments. Healthy cooking demonstration and food prepatation are already included in the text curriculum of home economics. If we include those as part of our intervention it will be a repetition. Therefore, we have selected the contents of nutrition education which are absent in their text curriculum.

Reviewer comment: Page # 16, paragraph 1: Revise the methodology to include MDD-W that uses 10 food groups rather than derive 9 out of 16 food groups as is currently proposed by the researcher.

Response: Thanks for your comments. Since we are using Individual Dietary Diversity Score (IDDS) instead of MDD-W, we are using 9 out of 16 food groups.

Reviewer comment: Page # 19, item Quantitative analysis: A score of 1 is assigned to each food group and the sum is then used as the score for M-DDW.

Response: We will not use MDDW to measure dietary diversity at individual level as we mentioned above.

Reviewer comment: Page # 63, Appendix 3, question 3: Please include a question (s) on use of sour/ citrus fruits, sour food adjuncts, local traditional sauce/ chutneys or addition of small amounts of meat or soya (if relevant) with plant sources of iron and folic acid (leafy vegetables, whole grain cereals, parboiled grains, legumes); preparation/processing methods such as germination, fermentation, if applicable or practiced. Such foods and practices act as iron absorption enhancers and improve the bioavailability of plant sources of iron and folic acid.

Response: Thank you. We have included the questions about the use of aforementioned foods and practices under Appendix-3.

Reviewer 2

Reviewer's Name: Dr Mohammad Mushtuq Husain
Reviewer's details:
Former PSO
Institute of Epidemiology, Disease Control and Research (IEDCR)
Email: mushtuq@dr.com
Mobile: 01552410445

Reviewer comment: This protocol is of high quality and original. It deserves financial support

Response: Thank you very much for your comments. We have received funding from Bill and Malinda Gates Foundation.
Reviewer comment: It will generate new knowledge which may be utilized for nutritional intervention among adolescent girls.
Response: Thank you for your valuable comment. We also expect that this study will generate new knowledge which may be utilized for nutritional intervention among adolescent girls.

Reviewer comment: In page 15 the flowchart shows that both intervention and control group will receive IFA and WASH, but in page 16 it is stated that in the control groups girls will only receive WASH only. Please address the inconsistency.

Response: Thanks for your observation. We have made correction at page 16, according to your suggestion.

Reviewer comment: Consent from headmaster, considering him as a legal guardian, may not be accepted. Please check with lawyer, whether headmaster are eligible to become legal guardian. (page 17)

Response: Thanks for your comments. Since our intervention will be school-based nutrition education, we will obtain initial approval from the school authority and headmaster of the school. We will take assent from the adolescent girls and we will also take consent from the mother or caregiver of the adolescent girls before including them in this study. As per your suggestion, we have mentioned it at page 17.

Other Support

Describe sources, amount, duration, and grant number of all other research funding currently granted to PI or under consideration.

Gender Analysis Tool:

In Relation to inadequate dietary diversity among the adolescent girls:	Are there sex differences in	How do biological differences between women and men influence their	How do the different roles and activities of men and women affect their	How do gender norms / values affect men and women's	How do access to, and control over resources affect men and women's
**Vulnerability:**<br><br>**Incidence ****<br>**Prevalence ****<br>**(male/female)**	*Although inadequate dietary diversity has been reported as 72% among the adolescent girls [1],there is a scarcity of data on this about adolescent boys.*	*There are no relevant data regarding biological differences between adolescent girls and boys that can influence their dietary diversity.*	*There is no available data regarding this issue.*	*In LMICs including Bangladesh, it is commonly practiced that male child get more preference than female child. Such kind of norms and values increase nutritional vulnerability of female child and adolescent girls.*	*There are no available data regarding this issue.*
**Health seeking behaviour**	*Not applicable*	*Not applicable*	*Not applicable*	*Not applicable*	*Not applicable*
**Ability to access health services**	*Not applicable*	*Not applicable*	*Not applicable*	*Not applicable*	*Not applicable*
**Experience with health services and health providers**	*We do not have any references to assume any difference*	*May not be applicable*	*May not be applicable*	*May not be applicable*	*Not applicable*

[1] James P Grant School of Public Health and National Nutrition Services. (2016). State of food security and nutrition in Bangladesh 2015. Dhaka, Bangladesh: James P Grant School of Public Health and National Nutrition Services.

In Relation to inadequate dietary diversity among the adolescent girls:	Are there sex differences in	How do biological differences between women and men influence their	How do the different roles and activities of men and women affect their	How do gender norms / values affect men and women's	How do access to, and control over resources affect men and women's
**Preventive and Treatment options, responses to treatment or rehabilitation**	*Not applicable*	*Not applicable*	*Not applicable*	*Not applicable*	*Not applicable*
**Outcome of health problem**	*We do not have any reference to assume any difference*	*Adolescent girls are nutritionally more vulnerable because peak velocity of their linear growth takes place approximately 6-12 months prior to menarche [2].*	*We do not have any reference to assume any difference*	*Adolescent girls face socio-cultural food taboos during their menstrual period*	*Not applicable*
**Consequences (economic & social, including attitudinal)**	*We do not have any reference to assume any difference*	*We do not have any reference to assume any difference*	*May not be applicable*	No information available	*We do not have any reference to assume any difference*

* Of different classes, ethnic groups, ages or other relevant differences between women and between men: Evidence shows that higher income groups are more likely to take preventive action; though also more likely to show resistance because of incorrect use of drugs.

Biography of the Investigators

Provide biographical data in the following format for all key personnel including the Principal Investigator. Copy the same format for each of them.
Note: Biography of the External Investigators may, however, be submitted in the format as convenient to them..

Biography of Mr. Mahfuzur Rahman

1. **Name:** Mahfuzur Rahman
2. **Present Position**: Research Investigator
3. **Educational background**: (last degree and diploma & training relevant to the present research proposal)

	Institution	Year
MPH	James P Grant School of Public Health, BRAC University	2015
MSS (in Anthropology)	Shahjalal University of Science and Technology, Sylhet, Bangladesh	2004
BSS (Honors) in Anthropology	Shahjalal University of Science and Technology, Sylhet, Bangladesh	2003
Training on	James P Grant School of Public Health, BRAC	2017

[2] Stang J, Story M. Adolescent growth and development. Guidelines for adolescent nutrition services. 2005;1(6).

Thinking Qualitatively	University	

4. Ethics Certification:

		If Yes		
		Issuing Authority	Registration No	Valid Until
**No** ☐	Yes ☒	NIH	1804690	Issued on July 27, 2015

5. List of ongoing research protocols/ activities

Protocol/ Activity Number	Role in the protocol/ activity (PI, Co-PI, Co-I)	Starting date	End date	Percentage of time
PR-17024	PI	May 2017	September, 2018	20%
PR-15090	Co-I	September, 2015	February, 2017	50%
PR-16060	Co-I	September, 2016	August, 2017	50%
PR-16056	Co-I	July, 2016	December, 2018	0%

6. Publications

Types of publications	Numbers
Original scientific papers in peer-review journals	02
Peer reviewed articles and book chapters	0
Papers in conference proceedings	03
Letters, editorials, annotations, and abstracts in peer-reviewed journals	0
Working papers	0
Monographs	0

7. Five recent publications including publications relevant to the present research protocol

7.1. Epstein A, Moucheraud C, Sarma H, **Rahman M**, Tariqujjaman M, Ahmed T, Glenn J, Bossert T, Kruk ME. Does health worker performance affect clients' health behaviors? A multilevel analysis from Bangladesh. BMC Health Services Research. (In Press). doi: 10.1186/s12913-019-4205-z.

7.2 Jannat K, **Rahman M**, Unicomb L, Levin D. The Disgust Box: A Novel Approach to Illustrate Water Contamination with Feces. Global Health Promotion. Published on August 1, 2016 as doi:10.1177/1757975916658638

Certificate of Completion

The National Institutes of Health (NIH) Office of Extramural Research certifies that **Mahfuzur Rahman** successfully completed the NIH Web-based training course "Protecting Human Research Participants".

Date of completion: 07/27/2015

Certification Number: 1804690

Biography of Dr. Zannatun Nyma

1. **Name:** Dr. Zannatun Nyma

2. **Present Position:** Research Fellow

3. **Educational background**: (last degree and diploma & training relevant to the present research proposal)

	Institution	Year
MPH	American International University Bangladesh	2017
MBBS	Dhaka Medical College	2015
Clinical and Epidemiological Research Study Design	icddr,b	2019
Case Based Scientific Writing	James P Grant School of Public Health, BRAC University	2017
Result Based Management	icddr,b	2017

Grant Proposal Writing	icddr,b	2017
Training Program on STATA	icddr,b	2017
Basic Course of Epidemiology, Biostatistics and SPSS	icddr,b	2015

4. Ethics Certification:

		If Yes		
		Issuing Authority	Registration No	Valid Until
**No** ☐	Yes ☒	**NIH**	**2802117**	

Note: If the response is "no", please get certification from CITI or NIH before study initiation and submit a copy to the Committee Coordination Secretariat

5. List of ongoing research protocols/ activities

Protocol/ Activity Number	Role in the protocol/ activity (PI, Co-PI, Co-I)	Starting date	End date	Percentage of time

6. Publications

Types of publications	Numbers
a. Original scientific papers in peer-review journals	0
b. Peer reviewed articles and book chapters	0
c. Papers in conference proceedings	0
d. Letters, editorials, annotations, and abstracts in peer-reviewed journals	0
e. Working papers	0
f. Monographs	0

7. Five recent publications including publications relevant to the present research protocol

7.1. N/A

7.2.

7.3.

7.4.

7.5.

CERTIFICATE
OF COMPLETION

PHRP Online Training, LLC certifies that

Zannatun Nyma

has successfully completed the web-based course
"Protecting Human Research Participants Online Training".

Date Completed: **06/23/2019**
Certification Number: **2802117**

PHRP
Protecting Human
Research Participants
Online Training

Biography of Dr. Tahmeed Ahmed

1. **Name:** Dr Tahmeed Ahmed

2. **Present Positions:** SeniorDirector, Nutrition &Clinical Services Division, icddr,b and Professor of Public Health Nutrition, James P. Grant School of Public Health, BRAC University

3. **Educational background**:

Degree	Institution	Year
PhD	University of Tsukuba, Japan	1996
MBBS	University of Dhaka	1983
Training	Clinical training in Pediatrics, University of Tsukuba Hospital	1990-1992
Training	Residential training in Pediatrics, Dhaka Shishu Hospital	1989-1990

4. **Ethics Certification:**

		Issuing Authority	Registration No	Valid Until
No☐	Yes ☒	NIH	1933611	Issued on 12 August 2015

5. **List of ongoing research protocols/ activities**

Protocol/ Activity Number	Role in protocol/ activity	Starting date	End date	% of time
Childhood acute illness and nutrition (CHAIN) study	Co-I	Nov 016	Aug 2019	10
Antibiotics for childhood diarrhea (ABCD) trial	Co-PI	Feb 2017	April 2020	20
Bangladesh Environmental Enteric Dysfunction (BEED) Study	PI	Nov 2015	April 2020	15
Evaluation of the largest stunting control program - Suchana	Co-PI	Jun 2016	Dec 2020	20
Microbiota directed complementary food clinical trials (Primary MAM and Post SAM-MAM)	PI	Nov 2018	Feb 2021	20

6. Publications

Types of publications	Numbers
Original scientific papers in peer-review journals	286
Book chapters	18
Papers in conference proceedings	25
Letters, editorials, annotations, and abstracts in peer-reviewed journals	5
Working papers	14
Monographs	1

7. Five recent publications including publications relevant to the present research protocol

1. Nahar B, Hossain M, Ickes SB, Naila NN, Mahfuz M, Hossain D, Denno DM, Walson J, Ahmed T. Development and validation of a tool to assess appetite of children in low income settings. Appetite 2018 Dec 21. pii: S0195-6663(18)30468-9.

2. Ahmed T, Choudhury N, Hossain I, TangsuphoomN, Islam MM, de Pee S, Steiger G, Fuli R, Sarker SA, Parveen M, West KP, Christian P. Development and acceptability testing of ready-to-use supplementary food made from locally available food ingredients in Bangladesh. BMC Pediatr 2014 Jun 27;14:164.

3. Subramanian S, Huq S, Yatsunenko T, Haque R, Mahfuz M, Alam MA, Benezra A, DeStefano J, Meier MF, Muegge BD, Barratt MJ, VanArendonk LG, Zhang Q, Province MA, Petri WA Jr, Ahmed T, Gordon JI. Persistent gut microbiota immaturity in malnourished Bangladeshi children. Nature 2014 doi:10.1038/nature13421.

4. Khatun H, Comins CA, Shah R, Islam MM, Choudhury N, Ahmed T. Uncovering the barriers to exclusive breastfeeding for mothers living in Dhaka's slums: a mixed method study. Int Breastfeed J 2018 Sep 26;13:44
5. Ahmed T, Auble D, Berkley JA, Black R, Ahern PP, Hossain M, Hsieh A, Ireen S, Arabi M, Gordon JI. An evolving perspective about the origins of childhood undernutrition and nutritional interventions that includes the gut microbiome. Ann N Y AcadSci 2014 Aug 12. [Epub ahead of print]
6. Chisti MJ, Salam MA, Smith JH, Ahmed T,Pietroni MAC, Shahunja KM,Shahid ASMSB,Faruque ASG, Ashraf H, Bardhan PK,Sharifuzzaman, Graham SM, Duke T.

Bubble continuous positive airway pressure for children with severe pneumonia and hypoxaemia in Bangladesh: an open, randomized controlled trial. Lancet 2015 Aug 19

Biography of Dr. Mustafa Mahfuz

1. **Name:** Dr. Mustafa Mahfuz
2. **Present Position:** Associate Scientist, Nutrition and Clinical Services Division

3. **Educational background**:

	Institution	Year
MPH	University of Dhaka	2006
MBBS	University of Chittagong	2001

4. **Ethics Certification:**

		If Yes		
		Issuing Authority	Registration No	Valid Until
**No** ☐	Yes ☒	NIH	1973495	Issued 2016

5. **List of ongoing research protocols/ activities**

Protocol/ Activity Number	Role in the protocol/ activity (PI, Co-PI, Co-I)	Starting date	End date	Percentage of time
PR-16007	Co-PI	15.12.2015	31.11.2019	60
PR-12096	PI	28.01.2013	30.06.2017	20
2008-20	Co-I	01.10.2008	31.08.2017	10
PR- 11005	Co-I	19.08.2011		10

6. **Publications**

Types of publications	Numbers
Original scientific papers in peer-review journals	66
Peer reviewed articles and book chapters	7
Papers in conference proceedings	4
Letters, editorials, annotations, and abstracts in peer-reviewed journals	7
Working papers	0
Monographs	0

7. **Five recent publications including publications relevant to the present research protocol**

1. **Mahfuz M**, Alam MA, Fahim SM, Gazi MA, Raihan MJ, Hossain M, Egner PA, et al. Aflatoxin exposure in children living in Mirpur, Dhaka: data from MAL-ED companion study. J Expo Sci Environ Epidemiol. 2018 Sep 5. doi: 10.1038/s41370-018-0066-5.
2. **Mahfuz M**, Alam MA, Islam SB, Naila N, Chisti MJ, Alam NH, Sarker SA, Ahmed T. Treatment outcome of children with Persistent Diarrhoea admitted to an Urban Hospital, Dhaka during 2012-2013. BMC Pediatrics 2017; 17:142.

3. **Mahfuz M**, Das S, Mazumder RN, Rahman M, Haque R, Gordon JI, Ahmed T et al. Bangladesh Environmental Enteric Dysfunction (BEED) study: Protocol for a community-based intervention study to validate non-invasive biomarkers of Environmental Enteric Dysfunction. BMJ Open 2017-017768 (accepted).
4. **Mahfuz M**, Alam MA, Islam MM, Mondal D, Hossain MI, Ahmed AMS, Choudhury N, Raihan MJ, Haque R, and Ahmed T. Effect of micronutrient powder supplementation for two and four months on hemoglobin level of children 6–23 months old in a slum in Dhaka: a community based observational study. BMC Nutrition 2016, DOI: 10.1186/s40795-016-0061-y. URL: http://www.biomedcentral.com/2055-0928/2/21
5. Subramanian S, Huq S, Yatsunenko T, Haque R, **Mahfuz M**, Alam MA, Benezra A, DeStefano J, Meier MF, Muegge BD, Barratt MJ, VanArendonk LG, Zhang Q, Province MA,. Petri WA, Ahmed T, Gordon JI. Persistent gut microbiota immaturity in malnourished Bangladeshi children (research letter). Nature 2014 Jun 19;510(7505):417-21.

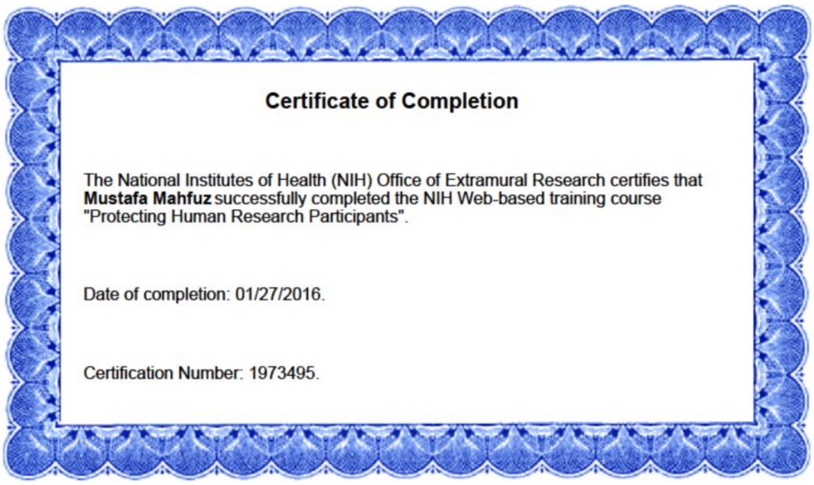
Certificate of Completion

The National Institutes of Health (NIH) Office of Extramural Research certifies that **Mustafa Mahfuz** successfully completed the NIH Web-based training course "Protecting Human Research Participants".

Date of completion: 01/27/2016.

Certification Number: 1973495.

Biography of Dr. Subhasish Das

1. **Name:** Dr. Subhasish Das

2. **Present Position:** Research Investigator, Nutrition and Clinical Services Division, icddr,b

3. **Educational background**:

	Institution	Year
MPH	James P Grant School of Public Health	2015
MBBS	University of Dhaka	2012

4. **Ethics Certification:**

		If Yes		
		Issuing Authority	Registration No	Valid Until
**No**	Yes	NIH	1932738	2019

5. List of ongoing research protocols/ activities

Protocol/ Activity Number	Role in the protocol/ activity (PI, Co-PI, Co-I)	Starting date	End date	Percentage of time
PR- 16007	Co-I			60
PR- 17065	PI			40

6. Publications

Types of publications	Numbers
Original scientific papers in peer-review journals	4
Peer reviewed articles and book chapters	0
Papers in conference proceedings	3
Letters, editorials, annotations, and abstracts in peer-reviewed journals	0
Working papers	0
Monographs	0

7. Five recent publications including publications relevant to the present research protocol

1. Mahfuz M, **Das S**, Mazumder RN, Rahman MM, Haque R, Bhuiyan MM, Akhter H, Sarker MS, Mondal D, Muaz SS, Karim AB. Bangladesh Environmental Enteric Dysfunction (BEED) study: protocol for a community-based intervention study to validate non-invasive biomarkers of environmental enteric dysfunction. BMJ open. 2017 Aug 1;7(8):e017768.
2. Fahim SM, **Das S**, Sanin KI, Gazi MA, Mahfuz M, Islam MM, Ahmed T. Association of Fecal Markers of Environmental Enteric Dysfunction with Zinc and Iron Status among Children at First Two Years of Life in Bangladesh. The American journal of tropical medicine and hygiene. 2018 Aug;99(2):489.
3. Fahim SM, **Das S**, Gazi MA, Mahfuz M, Ahmed T. Association of intestinal pathogens with fecal markers of environmental enteric dysfunction among slum dwelling children in the first two years of life in Bangladesh. Tropical Medicine & International Health. 2018 Aug 22.
4. Md Amran Gazi, Sultan Mahmud, Shah Mohammad Fahim, Mohammad Golam Kibria, Parag Palit, Md. Rezaul Islam, Humaira Rashid, **Subhasish Das**, Mustafa mahfuz, Tahmeed Ahmed. Functional prediction of hypothetical proteins from Shigella flexneri and validation of the predicted models by using ROC curve analysis. Interdisciplinary Sciences: Computational Life Sciences. INSC-D-18-00026.

Certificate of Completion

The National Institutes of Health (NIH) Office of Extramural Research certifies that **Subhasish Das** successfully completed the NIH Web-based training course “Protecting Human Research Participants”.

Date of completion: 12/07/2015

Certification Number: 1932738

গবেষণায় অংশগ্রহনের সম্মতিপত্র

(কিশোরীর সম্মতি পত্র)

গবেষণার শিরোনাম: **A Cluster Randomized Controlled Trial to Measure the Efficacy of School-Based Nutrition Education in Improving Dietary Diversity among Bangladeshi Adolescent Girls**

Protocol No.PR-19089	Version No.0.2	Version date: 05.08.2019

প্রধান গবেষকের নাম: মাহফুজুর রহমান

গবেষণার উদ্দেশ্যঃ

শৈশবের পর কৈশর (১০-১৯ বছর) একজন মানুষের শারীরিক বৃদ্ধির জন্য দ্বিতীয় প্রধান গুরুত্বপূর্ণ সময়। এই সময়ই একজন মানুষের মোট শারীরিক বৃদ্ধির শতকরা ৮০ ভাগই সম্পূর্ণ হয়ে যায়। পূর্ববর্তী গবেষণায় দেখা গিয়েছে যে, কৈশোরে হঠাৎ শারীরিক বৃদ্ধির জন্য তাদের পুষ্টি চাহিদাও অত্যন্ত বেড়ে যায় এবং এই সময়ে অপর্যাপ্ত খাদ্য বৈচিত্র্যের কারণে তাদের মধ্যে পুষ্টিহীণতা দেখা দেয়। যেহেতু একজন কিশোরী অদূর ভবিষ্যতে একজন মা হবেন এবং একজন মায়ের সঠিক দৈহিক বৃদ্ধি সরাসরি তার গর্ভে থাকা বাচ্চার দৈহিক বৃদ্ধির সাথে জড়িত, তাই একজন মায়ের সঠিক পুষ্টির মাধ্যমে সঠিক দৈহিক বৃদ্ধি অত্যন্ত দরকার। যেহেতু কৈশোরের (১০-১৪ বছর) পরে একজন মানুষের উল্লেখযোগ্য তেমন কোনো দৈহিক বৃদ্ধি হয় না, তাই মা থেকে শিশু পর্যন্ত সকলের অপুষ্টি দূর করার উপযুক্ত সময় হলো কৈশোর। এসব বিষয় বিবেচনা করে, আমরা কলেরা হাসপাতাল থেকে রংপুর বিভাগে একটি গবেষণা কার্যক্রম পরিচালনা করতে যাচ্ছি। আমরা রংপুর শহর থেকে দুটি উচ্চ বিদ্যালয় ও গ্রাম থেকে দুটি উচ্চ বিদ্যালয় নিয়ে সেখানকার ষষ্ঠ, সপ্তম ও অষ্টম শ্রেণীর কিশোরীদের উপর আমাদের গবেষণা কার্যক্রম পরিচালনা করবো। গবেষণা কার্যক্রমের অংশ হিসেবে আমরা সপ্তাহে একবার একটি শ্রেণীর সকল শিক্ষার্থীকে খাদ্য বৈচিত্র্যতা সম্পর্কে শিক্ষা দান করব। আমাদের এরকম মোট আটটি সেশন থাকবে এবং শিক্ষাদানের মোট সময় হবে তিন মাস। প্রত্যেক শিক্ষণীয় সেশনের সাথে সকল কিশোরীদের আয়রণ-ফলিক এসিড খাওয়ানো হবে। এই গবেষণার ফলাফল ভবিষ্যতে কিশোরীদের বৈচিত্রময় খাদ্য গ্রহণে সহায়তা করবে এবং সঠিক দৈহিক বৃদ্ধিতে ভূমিকা পালন করবে।

আপনাকে এই গবেষণায় অংশগ্রহনে আমন্ত্রণ জানানোর কারনঃ

আমরা পুষ্টি বিষয়ক শিক্ষার মাধ্যমে কিশোরী মেয়েদের খাদ্য-বৈচিত্র্যতার উন্নতি ঘটাতে চাই। আপনি যেহেতু আমাদের গবেষণার আওতাধীন স্কুলে অধ্যনরত একজন কিশোরী, সে কারণে আমরা আপনাকে আমাদের গবেষণায় অংশগ্রহণ করার জন্য আমন্ত্রণ জানাচ্ছি।

গবেষনায় আপনার কাছে প্রত্যাশাঃ

এই গবেষণা ১৪ মাস ধরে চলবে এবং আমরা আমাদের নির্ধারিত বালিকা উচ্চ বিদ্যালয়গুলোতে মোট তিনমাস খাদ্য বৈচিত্র্যতা সম্পর্কে শিক্ষাদান করব। আমাদের গবেষণা কর্মীরা আপনার নিকট থেকে মোট তিনবার তথ্য সংগ্রহ করবে : সেশন শুরুর আগে একবার, সেশন শেষ হওয়ার পরে এবং সেশন শেষ হওয়ার ৩ মাস পরে একবার। । তথ্য সংগ্রহের বিষয়সমূহ হলোঃ আপনার সামাজিক অবস্থা, খাদ্য বৈচিত্র্যতা, মাসিকের বিবরণ, খাদ্য ও পুষ্টির অবস্থা এবং রক্ত হতে হিমোগ্লোবিনের পরিমাণ। প্রতিবার তথ্য সংগ্রহের সময় আপনাকে প্রায় এক ঘন্টা সময় দিতে হতে পারে।

গবেষনায় অংশগ্রহনের ঝুঁকি এবং সুবিধাঃ

কিছু মুল্যবান সময় ব্যয় করা ছাড়া এই গবেষণায় আপনার তেমন কোন ক্ষতি হওয়ার ঝুঁকি নেই। আমরা খাদ্য বৈচিত্র্যতা নিয়ে যে শিক্ষাদান সেশনগুলো দিব সেগুলো আমাদের দক্ষ গবেষণা প্রতিনিধিগণ প্রজেক্টর এবং ল্যাপটপের মাধ্যমে বর্ণনা করবেন। ষষ্ঠ, সপ্তম ও অষ্টম শ্রেণীর সকল কিশোরীদের আমরা এই শিক্ষণীয় সেশনগুলো দিব। তার সাথে সাথে এই তিন শ্রেণীর সকলকে বাংলাদেশ সরকারের নির্ধারিত নীতিমালা অনুযায়ী সপ্তাহে একদিন আমাদের মহিলা কর্মীরা আয়রন-ফলিক এসিড ট্যাবলেট খাওয়াবেন। আপনার নিকট হতে আমাদের মহিলা গবেষণা কর্মীরা যথাযথ উপায়ে তথ্য নিবেন এবং গোপনীয়তা বজায় রাখবেন, তাই আপনাদের বিব্রত হওয়ার কোনো অবকাশ নেই। পরীক্ষা

নিরীক্ষার সময় আপনার যদি অতিমাত্রায় রক্তশূন্যতা ধরা পড়ে, তবে আপনাকে নিকটস্থ সরকারি স্বাস্থ্য কেন্দ্রে উপযুক্ত চিকিৎসার জন্য প্রেরণ করা হবে।

এই গবেষণায় আপনার অংশগ্রহন আমাদের দেশের কিশোরীদের এবং ভবিষ্যৎ মায়েদের সঠিক দৈহিক বৃদ্ধিতে বিশেষ ভুমিকা রাখবে বলে আশা করা যাচ্ছে এবং আপনাদের এই অবদান গবেষণা প্রতিবেদনে উল্লেখ করা হবে।

ক্ষতিপূরণ ঃ

গবেষণায় অংশগ্রহণ করার ফলে আপনার যে সময় ব্যয় হবে তার জন্য আপনাকে আর্থিক বা অন্য কোন ক্ষতিপূরণ দেয়া হবে না।

গোপনীয়তা ঃ

এই দেশের আইন অনুযায়ী আমরা আপনার দেওয়া সমস্ত তথ্যের গোপনীয়তা বজায় রাখার জন্য সর্বোচ্চ চেষ্টা করব। রেকর্ডকৃত সকল তথ্য গোপনীয়তার সাথে রক্ষণাবেক্ষণ করা হবে। আমরা শুধুমাত্র গবেষণার কাজে এই তথ্য ব্যবহার করব এবং আপনার নাম অথবা পরিচয়ের কোন সূত্র অর্থাৎ আপনাকে চেনা যাবে এমন কোন তথ্য কোথাও ব্যবহার করা হবে না ।

তথ্য সংরক্ষনঃ

গবেষনা শেষ হওয়ার পরও আপনার কাছ থেকে প্রাপ্ত তথ্য সমূহ তিন বছর পর্যন্ত সংরক্ষণ করা হবে যা গবেষনার রিপোর্ট বা প্রকাশনার কাজে ব্যবহার করা হবে।

তথ্যের ভবিষ্যৎ ব্যাবহারঃ

এই গবেষণায় সংগৃহীত তথ্য প্রয়োজনে অন্যান্য গবেষকের সাথে বিনিময় হতে পারে, কিন্তু আপনার ঠিকানা, পরিচয় ইত্যাদি গোপন রাখা হবে। এছাড়াও ভবিষ্যতে ভিন্ন গবেষনায় আপনার অংশগ্রহনের প্রয়োজনে যোগাযোগ করার জন্য আমরা আপনার নাম এবং বাড়ীর/খানার ঠিকানা ব্যবহার করতে পারি যদি আপনি আমাদের সে অনুমতি দেন। তবে অন্য কোন গবেষককে আপনার পরিচয় দেওয়া হবে না।

গবেষনায় অংশগ্রহণ না করা বা অংশগ্রহন প্রত্যাহার করার অধিকারঃ

এই গবেষণায় অংশগ্রহণ সম্পূর্ণভাবে আপনার ইচ্ছার ওপর নির্ভর করে। আপনি চাইলে যে কোন প্রশ্নের উত্তর নাও দিতে পারেন। আপনার লিখিত সম্মতি দেয়ার পরবর্তীতেও আপনি ইচ্ছা করলে যে কোন সময়ে এমনকি সাক্ষাৎকার প্রদানের মাঝখানেও আপনার অনুমতি ফিরিয়ে নিতে পারেন। এই গবেষণায় অংশগ্রহণ করা বা না করার অধিকার সম্পুর্ন আপনার এবং এ জন্য ভবিষ্যতে আপনার পরিবারের সদস্যদের কলেরা হাসপাতালে স্বাস্থ্যসেবা গ্রহণের উপর কোন নেতিবাচক প্রভাব পড়বে না। এমনকি আপনি আমাদের সেবা থেকেও (পুষ্টিবিষয়ক শিক্ষাদান, আয়রন-ফলিক এসিড ট্যাবলেট, নিরাপদ পানি ও পরিষ্কার পরিচ্ছন্নতা বিষয়ক তথ্য) বাদ যাবেন না।

যোগাযোগঃ

আপনার কোনো প্রশ্ন থাকলে আপনি এখনই আমাকে জিজ্ঞাসা করতে পারেন অথবা পরেও জিজ্ঞেস করতে পারেন। এছাড়াও এই গবেষণায় অংশগহণের অধিকার ও সুযোগ-সুবিধা সম্পর্কে যদি আরো জানতে চান, তাহলে নিচের ঠিকানায় সরাসরি যোগাযোগ করতে পারেন:

যোগাযোগের উদ্দেশ্য	নাম ও পরিচয়	যোগাযোগের ঠিকানা
গবেষণা সম্পর্কিত কোন প্রশ্ন, অনুসন্ধান বা অসুবিধা	জিল্লুর রহমান (স্থানীয় সমন্বয় কারী)	...................................<br>...................................<br>মোবাইল<br>মোবাইল (২৪ ঘন্টা) ঃ ০১৭২৬৩৩২৬৯৮
	মাহফুজুর রহমান (প্রধান গবেষক)<br>প্রতিষ্ঠানের নামঃ আর্তজাতিক উদ্রাময় গবেষণা কেন্দ্র, বাংলাদেশ (আইসিডিডিআর, বি)	এনসিএসডি, কলেরা হাসপাতাল, মহাখালি, ঢাকা-১২১২<br>মোবাইল ঃ ০১৮১৬৫৪৩৭১১ (সকাল ৯.০০-বিকাল ৫.০০)

গবেষণা সম্পর্কিত কোন অসন্তোষ বা অভিযোগ থাকলে	এম এ সালাম খান (আই আর বি কো-অরডিনেসক্রেটারিয়েট)	কলেরা হাসপাতাল, মহাখালি, ঢাকা-১২১২ মোবাইল ঃ ০১৭১১৪২৮৯৮৯ (সকাল ৯.০০-বিকাল ৫.০০) ঃ

আপনি যদি এই গবেষণায় অংশগ্রহণ করতে আগ্রহী হন তাহলে নিম্নের নির্ধারিত স্থানে টিক (√) চিহ্ন দিন এবং সবশেষে নির্ধারিত স্থানে স্বাক্ষর অথবা বাম বৃদ্ধাঙ্গুলীর ছাপ দিন। আপনার সহযোগিতার জন্য ধন্যবাদ।

আমি গবেষকের কাছে সাক্ষাৎকার প্রদানে সম্মতি দিচ্ছি	☐ হ্যাঁ	☐ না
আমি সাক্ষাৎকারটি অডিও রেকর্ড করার সম্মতি দিচ্ছি	☐ হ্যাঁ	☐ না
আমি আমার নাম পরিচয় প্রকাশ না করার শর্তে আমার থেকে প্রাপ্ত তথ্য অন্যান্য গবেষকদের দেয়ার সম্মতি দিচ্ছি	☐ হ্যাঁ	☐ না

....	....	....
অংশগ্রহনকারীর স্বাক্ষর/বাম বৃদ্ধাঙ্গুলির ছাপ	অংশগ্রহনকারীর নাম	তারিখ
....	....	....
অভিভাবকের স্বাক্ষর	সাক্ষীর নাম	তারিখ
....	....	....
সাক্ষীর স্বাক্ষর/বাম বৃদ্ধাঙ্গুলির ছাপ	সাক্ষীর নাম	তারিখ
....	....	....
গবেষক/তথ্য সংগ্রহকারীর স্বাক্ষর	গবেষক/তথ্য সংগ্রহকারীর নাম	তারিখ

Assent Form for Adolescent Girl (English)

Protocol No. PR-19089	Version No. 0.02	Date: 05-08-2019

Protocol Title: A Cluster Randomized Controlled Trial to Measure the Efficacy of School-Based Nutrition Education in Improving Dietary Diversity among Bangladeshi Adolescent Girls

Investigator's name: Mr. Mahfuzur Rahman

Organization: International Centre for Diarrhoeal Disease Research, Bangladesh (icddr,b)

Purpose of the research

After first year, adolescence (10-19 years) is the second most critical period for physical growth. Of the entire growth of the body, 80% is achieved during the early adolescence (10-15 years). It was revealed from the previous studies that this sudden growth spurt makes early adolescence a nutritionally vulnerable period and inadequate dietary diversity is one of the major causes of adolescent nutritional deficiency. As an adolescent girl is a future mother and offsprings growth is directly related to mother's proper growth. For this, proper nutrition of a mother is very crucial. Adult weight & height gain are ultimately ceased after early adolescence. So, adolescence is the last opportunity to intervene and break the vicious cycle of inter-generational malnutrition. Keeping all these contexts in mind, we aim to conduct a research in Rangpur district. We will select two urban and two rural schools from Rangpur district and from each school we will select three grades which will be grade six, seven and eight. We will provide eight nutrition education sessions regarding dietary diversity to adolescent girls. Each session will be conducted in one grade once a week and our total duration of the intervention will be three months. Along with the nutrition education session, we will give iron-folic acid supplementation to all the students under the selected grades, once a week for three months. The result of the study will generate knowledge on how to improve dietary diversity among the adolescent girls and thereby improve their nutritional status.

Why invited to participate in the study?

We are trying to provide nutrition education to adolescent girls to measure the improvement of dietary diversity. You are an adolescent girl studing in a school selected for our study and this is why we are inviting you to help us by participating in this study.

Methods and procedures

Our research will be conducted for 14 months and we will provide nutrition education as intervention for three months to the selected schools. Our skilled female research staffs will collect data at three different time points: before rolling out of the intervention, after completion of the intervention and after three months of intervention. Our female staffs will collect data regarding demographic information, your dietary diversity, menstrual history and health as well as nutritional status (height, weight, MUAC and hemoglobin concentration). It will take about an hour to collect data.

Risk and benefits

Apart from spending of valuable time, you will not face any risk due to participation in the study. We will provide our intervention (nutrition education sessions) using projector and laptop by skilled research staffs. We will provide these sessions to all of the students of grade six, seven and eight. In addition, our female staffs will provide iron-folic acid tablets to all students of these mentioned classes once a week (it is the recommended dose of Bangladesh Government). Your information will be collected by our skilled female staffs and they will

maintain confidentiality, so we would expect that it will not be a cause of your embarrassment. During investigation, if we find that you have been suffering from severe anaemia, we will refer you to the nearest health care centre.

Your participation in this study will help the adolescent girls to achieve appropriate growth and we will mention your contribution in our research.

Principle of compensation
You will not be given any financial or other compensation for the time you spend as a result of participating in the study.

Privacy, anonymity and confidentiality
According to the laws of this country, we do hereby affirm that privacy; anonymity and confidentiality of the information provided by you will strictly be maintained. Information provided by you will be kept confidential and will only be used for research purposes in this study with various segments. We will not mention your name or your identity or any kind of information by which you could be recognized.

Storage of data

Even after completion of the research, your shared information will be stored for three years which will be used for research report or publications.

Future use of information
Collected information of this research could be shared by other researchers but we will maintain confidentiality in case of your identity and address. Moreover, if you give us permission, we will use your name and address to make contact with you for participating in another research in future.

Right not to participate and withdraw

Your participation in this study is voluntary. You may not answer any questions if you want. You can withdraw yourself from the study even after giving informed written consent or any time during interview session. It is entirely your right whether you will participate in this research or not and there will be no negative impact of your decision on taking service from icddr,b in future. You will receive nutrition education, message on water, sanitation and hygiene, and IFA supplementation just like other participants.

Communication:
If you have any question, you can ask me right now or at any time later on. If you want to know anything about rights and benefits for participation in this study you can contact in the following addresses:

Purpose of contact	Name and address	Address for communication
For any question related to the study, or any problem	Mr. Zillur Rahman (local coordinator or representative)	Address: ______________________<br>______________________________<br>Mobile No.01726332698<br>(to be open 7/24 hours)
	Mr. Mahfuzur Rahman (PI)	Address:______________________<br>______________________________<br>Mobile: 01816543711<br>(9:00 am to 5:00 pm)
To know the rights or benefits or to log any complain or	M A Salam Khan (IRB Coordinator)	IRB Secretariat, Research Administration, icddr,b, Mohakhali, Dhaka-1212

dissatisfaction		Phone: (+88-02) 9827084 or<br>Mobile: 01711428989

If you agree to our proposal for enrolling you/your patient in our study, please put √ mark on appropriate box(es) of the following and finally sign on the specified place for you.

Thank you for your cooperation.

I am giving my consent for interview to the investigator	☐ Yes	☐ No
I am giving my consent to do audio for this interview	☐ Yes	☐ No
I am giving consent to give my provided information to other researchers on condition that my identifiable information cannot be disclosed or shared with anybody.	☐ Yes	☐ No

______________________________ ______________

Signature or left thumb impression of participant Date

______________________________ ______________

Signature or left thumb impression of Legal guardian Date

______________________________ ______________

Signature or left thumb impression of the witness Date

______________________________ ______________

Signature of the PI or his/her representative Date

গবেষণায় অংশগ্রহনের সম্মতিপত্র

(মায়ের সম্মতি পত্র)

গবেষণার শিরোনাম: **A Cluster Randomized Controlled Trial to Measure the Efficacy of School Based Nutrition Education in Improving Dietary Diversity among Bangladeshi Adolescent Girls**

Protocol No.PR-19089	Version No.02	Version date:05.08.2019

প্রধান গবেষকের নাম: মাহফুজুর রহমান

গবেষণার উদ্দেশ্যঃ

শৈশবের পর কৈশর (১০-১৯ বছর) একজন মানুষের শারীরিক বৃদ্ধির জন্য দ্বিতীয় প্রধান গুরুত্বপূর্ণ সময়। এই সময়ই একজন মানুষের মোট শারীরিক বৃদ্ধির শতকরা ৮০ ভাগই সম্পূর্ণ হয়ে যায়। পূর্ববর্তী গবেষণায় দেখা গিয়েছে যে, কৈশোরে হঠাৎ শারীরিক বৃদ্ধির জন্য তাদের পুষ্টি চাহিদাও অত্যন্ত বেড়ে যায় এবং এই সময়ে অপর্যাপ্ত খাদ্য বৈচিত্র্যের কারণে তাদের মধ্যে পুষ্টিহীণতা দেখা দেয়।যেহেতু একজন কিশোরী অদূর ভবিষ্যতে একজন মা হবেন এবং একজন মায়ের সঠিক দৈহিক বৃদ্ধি সরাসরি তার গর্ভে থাকা বাচ্চার দৈহিক বৃদ্ধির সাথে জড়িত, তাই একজন মায়ের সঠিক পুষ্টির মাধ্যমে সঠিক দৈহিক বৃদ্ধি অত্যন্ত দরকার। যেহেতু কৈশোরের (১০-১৪ বছর) পরে একজন মানুষের উল্লেখযোগ্য তেমন কোনো দৈহিক বৃদ্ধি হয় না, তাই মা থেকে শিশু পর্যন্ত সকলের অপুষ্টি দূর করার উপযুক্ত সময় হলো কৈশোর। এসব বিষয় বিবেচনা করে, আমরা কলেরা হাসপাতাল থেকে রংপুর বিভাগে একটি গবেষণা কার্যক্রম পরিচালনা করতে যাচ্ছি। আমরা রংপুর শহর থেকে দুটি উচ্চ বিদ্যালয় ও গ্রাম থেকে দুটি উচ্চ বিদ্যালয় নিয়ে সেখানকার ষষ্ঠ, সপ্তম ও অষ্টম শ্রেণীর কিশোরীদের উপর আমাদের গবেষণা কার্যক্রম পরিচালনা করবো। গবেষণা কার্যক্রমের অংশ হিসেবে আমরা সপ্তাহে একবার একটি শ্রেণীর সকল শিক্ষার্থীকে খাদ্য বৈচিত্র্যতা সম্পর্কে শিক্ষা দান করব। আমাদের এরকম মোট আটটি সেশন থাকবে এবং শিক্ষাদানের মোট সময় হবে তিন মাস। প্রত্যেক শিক্ষণীয় সেশনের সাথে সকল কিশোরীদের আয়রণ-ফলিক এসিড খাওয়ানো হবে। এই গবেষণার ফলাফল ভবিষ্যতে কিশোরীদের বৈচিত্রময় খাদ্য গ্রহণে সহায়তা করবে এবং সঠিক দৈহিক বৃদ্ধিতে ভূমিকা পালন করবে।

আপনাকে এই গবেষণায় অংশগ্রহনে আমন্ত্রণ জানানোর কারনঃ

আমরা পুষ্টি বিষয়ক শিক্ষার মাধ্যমে কিশোরী মেয়েদের খাদ্য-বৈচিত্র্যতার উন্নতি ঘটাতে চাই। আপনাকে এই গবেষনায় অংশগ্রহনের আমন্ত্রন জানাচ্ছি কারণ আপনি কিশোরী মেয়েটির (নাম উল্লেখ করুন) একজন অভিভাবক যে মেয়েটি আমাদের গবেষণার আওতাধীন বিদ্যালয়ে পড়াশুনা করে।

গবেষনায় আপনার কাছে প্রত্যাশাঃ

এই গবেষণা ১৪ মাস ধরে চলবে। আমরা রংপুর শহরের ২টি এবং গ্রামের ২টি উচ্চ বিদ্যালয়ে কিশোরী মেয়েদের স্কুলভিত্তিক পুষ্টিবিষয়ক শিক্ষা দিব। আমরা এজন্য ষষ্ঠ, সপ্তম ও অষ্টম শ্রেণীর শিক্ষার্থীদের নির্বাচন করেছি। আমরা সপ্তাহে ১দিন করে ১টি ক্লাসে পুষ্টিবিষয়ক শিক্ষা সেশন দিব। তার সাথে আমরা সপ্তাহে একদিন করে মোট ৩মাস আয়রন-ফলিক এসিড খাওয়াবো। আমরা স্কুলের কিশোরী মেয়েদের কাছ থেকে পুষ্টিবিষয়ক সেশনগুলো শুরু হওয়ার আগে একবার, সেশনগুলো শেষ হওয়ার পরে একবার এবং সেশন শেষ হওয়া থেকে তিনমাস পরে একবার (মোট তিনবার) তথ্য সংগ্রহ করব। আমাদের গবেষণা কর্মীরা আপনার নিকট হতেও উক্ত তিন সময়ে (মোট তিনবার) তথ্য সংগ্রহ করবে। আমাদের গবেষণা কর্মীরা আপনার নিকট হতে আপনার পরিবারের আর্থ-সামাজিক অবস্থা, পরিবারের পরিষ্কার-পরিচ্ছন্নতা, পানি পরিশোধন সম্পর্কিত তথ্য এবং আপনার বাড়ী/খানার খাদ্য বৈচিত্র্যতা সম্পর্কে তথ্য সংগ্রহ করবে।

আপনার নিকট থেকে সংগৃহীত তথ্য কখনই তথ্য বিশ্লেষণ ছাড়া অন্য কোনো কাজে ব্যবহার করা হবে না। প্রত্যেকবার তথ্য সংগ্রহে আপনাকে প্রায় এক ঘন্টা সময় দিতে হতে পারে। আমরা আপনাদের নিয়ে (কিশোরী মেয়েদের অভিভাবক) স্কুলে একটি সভার আয়োজন করব। আপনাদের সরব উপস্থিতি আমাদের একান্ত কাম্য।

গবেষনায় অংশগ্রহনের ঝুঁকি এবং সুবিধাঃ

কিছু মুল্যবান সময় ব্যয় করা ছাড়া এই গবেষনায় আপনার তেমন কোন ক্ষতি হওয়ার ঝুঁকি নেই। আমরা খাদ্য বৈচিত্র্যতা নিয়ে যে শিক্ষাদান সেশনগুলো দিব সেগুলো আমাদের দক্ষ গবেষণা প্রতিনিধিগণ প্রজেক্টর এবং ল্যাপটপের মাধ্যমে বর্ণনা করবেন।এই গবেষণায় অংশগ্রহণকারী সকল শিক্ষার্থীদের (ষষ্ঠ, সপ্তম ও অষ্টম) আমরা পুষ্টি বিষয়ক শিক্ষাদানের মাধ্যমে খাদ্য বৈচিত্র্যতা সম্পর্কে জ্ঞান দান করবো এবং তাদের বাংলাদেশ সরকারের নির্ধারিত নীতিমালা অনুযায়ী সপ্তাহে একদিন আয়রন-ফলিক এসিড ট্যাবলেট খাওয়াবো। যদি পরীক্ষা নিরীক্ষায় আপনার কিশোরী মেয়েটির (নাম উল্লেখ করুন) অধিক রক্তশূন্যতা ধরা পড়ে, তবে তাকে অবশ্যই নিকটস্থ স্বাস্থ কেন্দ্রে প্রেরণ করা হবে।

এই গবেষনায় আপনার অংশগ্রহন, আমাদের দেশের কিশোরী মেয়েদের খাদ্য বৈচিত্র্যতা উন্নয়নে বিশেষ ভুমিকা রাখবে বলে আশা করা যাচ্ছে এবং আপনাদের এই অবদান গবেষনা প্রতিবেদনে উল্লেখ করা হবে।

ক্ষতিপূরণ ঃ

গবেষণায় অংশগ্রহণ করার ফলে আপনার যে সময় ব্যয় হবে তার জন্য আপনাকে আর্থিক বা অন্য কোন ক্ষতিপুরন দেয়া হবে না।

গোপনীয়তা ঃ

এই দেশের আইন অনুযায়ী আমরা আপনার দেওয়া সমস্ত তথ্যের গোপনীয়তা বজায় রাখার জন্য সর্বোচ্চ চেষ্টা করব। রেকর্ডকৃত সকল তথ্য গোপনীয়তার সাথে রক্ষণাবেক্ষণ করা হবে। আমরা শুধুমাত্র গবেষণার কাজে এই তথ্য ব্যবহার করব এবং আপনার নাম অথবা পরিচয়ের কোন সূত্র অর্থাৎ আপনাকে চেনা যাবে এমন কোন তথ্য কোথাও ব্যবহার করা হবে না।

তথ্য সংরক্ষনঃ

গবেষনা শেষ হওয়ার পরও আপনার কাছ থেকে প্রাপ্ত তথ্য সমূহ তিন বছর পর্যন্ত সংরক্ষণ করা হবে যা গবেষনার রিপোর্ট বা প্রকাশনার কাজে ব্যবহার করা হবে।

তথ্যের ভবিষ্যৎ ব্যবহারঃ

এই গবেষণায় সংগৃহীত তথ্য প্রয়োজনে অন্যান্য গবেষকের সাথে বিনিময় হতে পারে, কিন্তু আপনার ঠিকানা, পরিচয় ইত্যাদি গোপন রাখা হবে। এছাড়াও ভবিষ্যতে ভিন্ন গবেষনায় আপনার অংশগ্রহনের প্রয়োজনে যোগাযোগ করার জন্য আমরা আপনার নাম এবং বাড়ীর/খানার ঠিকানা ব্যবহার করতে পারি যদি আপনি আমাদের সে অনুমতি দেন।

গবেষনায় অংশগ্রহণ না করা বা অংশগ্রহন প্রত্যাহার করার অধিকারঃ

এই গবেষণায় অংশগ্রহণ সম্পূর্ণভাবে আপনার ইচ্ছার ওপর নির্ভর করে। আপনি চাইলে যে কোন প্রশ্নের উত্তর নাও দিতে পারেন। আপনার লিখিত সম্মতি দেয়ার পরবর্তীতেও আপনি ইচ্ছা করলে যে কোন সময়ে এমনকি সাক্ষাৎকার প্রদানের মাঝখানেও আপনার অনুমতি ফিরিয়ে নিতে পারেন। এই গবেষণায় অংশগ্রহণ করা বা না করার অধিকার সম্পুর্ন আপনার এবং এ জন্য ভবিষ্যতে আপনার পরিবারের সদস্যদের কলেরা হাসপাতালে স্বাস্থ্যসেবা গ্রহণের উপর কোন নেতিবাচক প্রভাব পড়বে না। আপনি যদি আমাদের তথ্য প্রদানে অসম্মতি জানান, তবে আমরা আপনার মেয়ের নিকট হতেও তথ্য গ্রহণ থেকে বিরত থাকব। তবে ....(মেয়েটির নাম উল্লেখ করুন) আমাদের সেবা থেকে (পুষ্টিবিষয়ক শিক্ষাদান, আয়রন-ফলিক এসিড ট্যাবলেট, নিরাপদ পানি ও পরিষ্কার পরিচ্ছন্নতা বিষয়ক তথ্য) বাদ যাবে না।

যোগাযোগঃ

আপনার কোনো প্রশ্ন থাকলে আপনি এখনই আমাকে জিজ্ঞাসা করতে পারেন। এছাড়াও এই গবেষণায় অংশগহণের অধিকার ও সুযোগ-সুবিধা সম্পর্কে যদি আরো জানতে চান, তাহলে নিচের ঠিকানায় সরাসরি যোগাযোগ করতে পারেন:

যোগাযোগের উদ্দেশ্য	নাম ও পরিচয়	যোগাযোগের ঠিকানা
গবেষণা সম্পর্কিত কোন প্রশ্ন, অনুসন্ধান বা অসুবিধা	জিল্লুর রহমান (স্থানীয় সমন্বয়কারী)	.... মোবাইল মোবাইল (২৪ ঘন্টা)ঃ ০১৭২৬৩৩২৬৯৮

	মাহফুজুর রহমান (প্রধান গবেষক)	এনসিএসডি, কলেরা হাসপাতাল, মহাখালি, ঢাকা-১২১২<br>মোবাইল ঃ ০১৮১৬৫৪৩৭১১ (সকাল ৯.০০-বিকাল ৫.০০)
গবেষণা সম্পর্কিত কোন অসন্তোষ বা অভিযোগ থাকলে	এম এ সালাম খান (আই আর বি কো-অরডিনেসক্রেটারিয়েট)	কলেরা হাসপাতাল, মহাখালি, ঢাকা-১২১২<br>মোবাইলঃ ০১৭১১৪২৮৯৮৯ (সকাল ৯.০০-বিকাল ৫.০০)ঃ

আমি গবেষকের কাছে সাক্ষাৎকার প্রদানে সম্মতি দিচ্ছি ☐ হ্যাঁ ☐ না

আমি সাক্ষাৎকারটি অডিও রেকর্ড করার সম্মতি দিচ্ছি ☐ হ্যাঁ ☐ না

আমি আমার নাম পরিচয় প্রকাশ না করার শর্তে আমার থেকে প্রাপ্ত তথ্য অন্যান্য গবেষকদের দেয়ার সম্মতি দিচ্ছি ☐ হ্যাঁ ☐ না

....	....	....
অংশগ্রহনকারীর স্বাক্ষর/বাম বৃদ্ধাঙ্গুলির ছাপ	অংশগ্রহনকারীর নাম	তারিখ
....	....	....
সাক্ষীর স্বাক্ষর/বাম বৃদ্ধাঙ্গুলির ছাপ	সাক্ষীর নাম	তারিখ
....	....	....
গবেষক/তথ্য সংগ্রহকারীর স্বাক্ষর	গবেষক/তথ্য সংগ্রহকারীর নাম	তারিখ

আপনি যদি এই গবেষণায় অংশগ্রহণ করতে আগ্রহী হন তাহলে নিম্নের নির্ধারিত স্থানে টিক (√) চিহ্ন দিন এবং সবশেষে নির্ধারিত স্থানে স্বাক্ষর অথবা বাম বৃদ্ধাঙ্গুলীর ছাপ দিন। আপনার সহযোগিতার জন্য ধন্যবাদ।

Consent Form (English)
(For Mother or Legal Guardian of the Adolescent Girl)

Protocol No. PR-19089	Version No. 0.02	Date: 05-08-2019

Protocol Title: **A Cluster Randomized Controlled Trial to Measure the Efficacy of School-Based Nutrition Education in Improving Dietary Diversity among Bangladeshi Adolescent Girls**

Investigator's name: Mr. Mahfuzur Rahman

Organization: International Centre for Diarrhoeal Disease Research Bangladesh (icddr,b)

Purpose of the research

After first year, adolescence (10-19 years) is the second most critical period for physical growth. Of the entire growth of the body, 80% is achieved during the early adolescence (10-15 years). It was revealed from the previous studies that this sudden growth spurt makes early adolescence a nutritionally vulnerable period and inadequate dietary diversity is one of the major causes of adolescent nutritional deficiency. As an adolescent girl is a future mother and offsprings growth is directly related to mother's proper growth. For this, proper nutrition of a mother is very crucial. Adult weight & height gain are ultimately ceased after early adolescence. So, adolescence is the last opportunity to intervene and break the vicious cycle of inter-generational malnutrition. Keeping all these contexts in mind, we aim to conduct a research in Rangpur district. We will select two urban and two rural schools from Rangpur district and from each school we will select three grades which will be grade six, seven and eight. We will provide eight nutrition education sessions regarding dietary diversity to adolescent girls. Each session will be conducted in one grade once a week and our total duration of the intervention will be three months. Along with the nutrition education session, we will give iron-folic acid supplementation to all the students under the selected grades, once a week for three months. The result of the study will generate knowledge on how to improve dietary diversity among the adolescent girls and thereby improve their nutritional status.

Why invited to participate in the study?

We are trying to provide nutrition education to adolescent girls to measure the improvement of dietary diversity. We are inviting you to our study because you are a mother/legal guardian of the adolescent girl (please mention the name of the girl) who is studing in the selected school under our research.

Methods and procedures

The duration of our research will be fourteen months. We will provide nutrition education in two urban and two rural secondary high schools of Rangpur district. For intervention, we will select the girls from sixth, seventh and eighth grade of each school. We will provide one nutrition session in one grade in a week. Along with this, we will give iron-folic acid (IFA) supplementation once a week for three months. We will collect data at three different time points: at the beginning of the intervention, at the end of the intervention and again after three months of the intervention. Our total intervention period will be three months. Our skilled research staffs will collect data from you regarding socio-economic condition, water, sanitation and hygiene and dietary diversity of your family.

The information collected from you will never be used for any purpose other than analysis. It will take about an hour to complete data collection. Moreover, we will arrange a parents' meeting at the schools before intervention. We will highly appreciate your presence in the parents' meeting.

Risk and benefits

Apart from spending of valuable time, you will not face any risk due to participation in the study.We will provide our intervention (nutrition education sessions) using projector and laptop by skilled research staffs. We will provide these sessions to all the students of grade six, seven and eight. In addition, our female staffs will provide iron-folic acid tablets to all the students once a week (as recommended by the Government of Bangladesh). During investigation, if we find that the adolescent girl (please mention the name of the girl) has been suffering from severe anaemia, we will refer her to the nearest health care centre.

Your participation in this study will help the adolescent girls to achieve appropriate growth and we will mention your contribution in our research.

Principle of compensation

You will not be given any financial or other compensation for the time you spend as a result of participating in the study.

Privacy, anonymity and confidentiality

According to the laws of this country, we do hereby affirm that privacy; anonymity and confidentiality of the information provided by you will strictly be maintained. Information provided by you will be kept confidential and will only be used for research purposes in this study with various segments. We will not mention your name or your identity or any kind of information by which you could be recognized.

Storage of data

Even after completion of the research, your shared information will be stored for three years which will be used for research report or publications.

Future use of information

Collected information of this research could be shared by other researchers but we will maintain confidentiality in case of your identity and address. Moreover, if you give us permission, we will use your name and address to make contact with you for participating in another research in future.

Right not to participate and withdraw

Your participation in this study is voluntary. You may not answer any questions if you want. You can withdraw yourself from the study even after giving informed written consent or any time during interview session. It is entirely your right whether you will participate in this research or not and there will be no negative impact of your decision on taking service from icddr,b in future. If you refuse to participate in this study, we will abstain from including the adolescent girl (please mention the name of the girl) in our study. But she will receive nutrition education, messages on water, sanitation and hygiene, and IFA supplementation like other adolescent girls.

Communication:

If you have any question, you can ask me right now or at any time later on. If you want to know anything about rights and benefits for participation in this study you can contact in the following addresses:

Purpose of contact	Name and address	Address for communication
For any question related to the study, or any problem	Mr. Zilllur Rahman (local coordinator or representative)	Address: ______________________<br>______________________________<br>Mobile No.01726332698<br>(to be open 7/24 hours)
	Mr. Mahfuzur Rahman (PI)	Address:______________________<br>______________________________<br>Mobile: 01816543711<br>(9:00 am to 5:00 pm)
To know the rights or benefits or to log any complain or dissatisfaction	M A Salam Khan (IRB Coordinator)	IRB Secretariat, Research Administration, icddr,b, Mohakhali, Dhaka-1212<br>Phone: (+88-02) 9827084 or Mobile: 01711428989

If you agree to our proposal for enrolling you/your patient in our study, please put √ mark on appropriate box(es) of the following and finally sign on the specified place for you.

Thank you for your cooperation.

I am giving my consent for interview to the investigator ☐ Yes ☐ No

I am giving my consent to do audio for this interview ☐ Yes ☐ No

I am giving consent to give my provided information to other researchers on condition that my identifiable information cannot be disclosed or shared with anybody. ☐ Yes ☐ No

_______________________________________ ____________________

Signature or left thumb impression of participant Date

_______________________________________ ____________________

Signature or left thumb impression of the witness Date

_______________________________________ ____________________

Signature of the PI or his/her representative Date

Appendix 1: Survey questionnaire for adolescent girls

A Cluster Randomized Controlled Trial to Measure the Efficacy of School-Based Nutrition Education in Improving Dietary Diversity among Bangladeshi Adolescent Girls

TO BE FILLED IN BY FIELD INTERVIEWER

**Information**
**Respondent id :** Respondent identifier : |__|__| **School:** School identifier : |__|__| **iid :** Interviewer identifier : |__|__| **iid.name :** Name of interviewer : |__|__|__|__|__|__|__| <br>Name of School :<br><br>Name of Union :<br><br>Name of Upazilla :<br><br>School Setting:<br><br>Name of District :<br><br>Name of Division :<br><br>Name of mother or caregiver of the adolescent girl at household:<br><br>Contact number of head of household :<br>**hh.contact :** |__|__|__|__|__|__|__|__|__|__|__|__|__|__|__| <br>**Date of interview:** Date: |__|__|/|__|__|/|__|__|__|__| D D / M M / Y Y Y Y

Demographic Information of the index adolescent girl

**Question number**	**Questions**	**Responses**	**Code**
**DI01**	How old are you?<br>আপনার বয়স কত?	|__||__| **Years** |__||__| **Months**	
**DI02**	In which grade are you currently studing?<br>আপনি কোন ক্লাসে<br>পড়ছেন ?	|__||__|	
**DI03**	What is the birth order of you?<br>আপনি আপনার পিতামাতার কত নম্বর সন্তান?		
**DI04**	What is your religion?<br>আপনার ধর্ম কি?	Hindu / হিন্দু = 1<br>Islam/ ইসলাম = 2	

		Christian/ খ্রীস্টান = 3<br>Buddhist/ বৌদ্ধ = 4<br>None/ কোন ধর্মের অনুসারী নয় = 5<br>Prefer not to say/বলতে ইচ্ছুক নয় =6<br>Others, specify :_ / অন্যান্য = 7	|__|

Adolescent Dietary Diversity

Since this time yesterday what food did [NAME OF ADOLESCENT GIRL] eat?
Probe: Anything else
গতকাল এই সময় থেকে (সাক্ষাৎকার শুরু করার সময় থেকে বিবেচনা করে) এখন পর্যন্ত কি কি খেয়েছে?
Probe করুনঃ আর কিছু
Tick or cross ALL boxes!
টিক চিহ্ন দিন

Question number	Food Group	Question (with example)	Response	Code
**DD01**	Cereals	In the last 24 hours, did you eat any food made from grain such as millet, wheat, rice, maize, semolina, atta=flour, noodles, porridge, jau?<br>গত ২৪ ঘণ্টায় শস্যদানা থেকে প্রাপ্ত খাদ্য যেমন ভুট্টা, ধান বা গমের আটা, ময়দা, চাল/ভাত, চিড়া, মুড়ি, রুটি, পাউরুটি এবং অন্যান্য শস্যজাত খাবার, জাও, সুজি খেয়েছেন কি?	Yes/হ্যাঁ=1<br>No<br>/না= 2	|___|
**DD02**	White roots and tubers	In the last 24 hours, did you eat any food made from roots or tubers such as white potato, white yam, radish, turnip, wol etc?<br>গত ২৪ ঘণ্টায় গাছের মুল বা কান্ড যেমন আলু, কেশর আলু, সাদা আলু, কচু, মূলা, শালগম, ওল থেকে প্রস্তুতকৃত খাদ্য খেয়েছেন কি?	Yes/হ্যাঁ=1<br>No/না= 2	|___|
**DD03**	Vitamin A rich vegetables and tubers	In the last 24 hours, did you eat any food made from pumpkin, carrot, sweet potato, vegetables that have yellow or orange flesh etc?<br>গত ২৪ ঘণ্টায় লাউ, কুমড়া, মিষ্টি কুমড়া, গাজর, শসা এরকম লাল বা হলুদ শাকসবজি খেয়েছেন কি?	Yes/হ্যাঁ=1<br>No/না= 2	|___|
**DD04**	Dark green leafy vegetables	In the last 24 hours, did you eat any food made from any dark green leafy vegetables such as palang, puishak, mula shak, pat shak, sarso, spring onion and radish leaves, etc?<br>গত ২৪ ঘণ্টায় যেকোনো গাঢ় সবুজ শাকসবজি যেমন পালং শাক, পুঁই শাক, পাট শাক, মুলা শাক, ধনে পাতা, পিয়াজ শাক ইত্যাদি খেয়েছেন কি?	Yes/হ্যাঁ=1<br>No/না= 2	|___|
**DD05**	Other vegetables	In the last 24 hours, did you eat any other vegetables like tomatoes, eggplant, onion, cauliflower, cabbage etc?<br>গত ২৪ ঘণ্টায় অন্যান্য সবজি যেমন টমেটো, বেগুন, পেঁয়াজ ইত্যাদি খেয়েছেন কি?	Yes/হ্যাঁ=1<br>No/না= 2	|___|
**DD06**	Vetamin A	In the last 24 hours, did you eat any ripe	Yes/হ্যাঁ=1	|___|

	rich fruits	mango, ripe papaya, cantaloupe, watermelon etc and juice from these fruits?<br>গত ২৪ ঘণ্টায় পাকা আম, পাকা পেঁপে, তরমুজ, খরমুজ ইত্যাদি এবং এগুলোর জুস খেয়েছেন কি?	No/না= 2	
**DD07**	Other fruits	In the last 24 hours, did you eat any fruits like apple, green mango, banana, pomegranate, lemon, guava, jackfruit, pineapple, pawpaw, orange, lychee, plum, black berries, dates, palms, dalims, kamranga, jambura, latkon, bel etc and juice from these fruits?<br>গত ২৪ ঘণ্টায় আপেল, কাঁচা আম, কলা, লেবু, পেয়ারা, কাঁঠাল, ডালিম, পেঁপে, আনারস, বরই, কালো জাম, কামরাঙ্গা, জাম্বুরা, কমলা, বেল, লটকন, তাল, লিঁচু, ইত্যাদি এবং এগুলোর জুস খেয়েছেন কি?	Yes/হ্যাঁ=1<br>No/না= 2	|___|
**DD08**	Organ meat	In the last 24 hours, did you eat any liver, kidney, heart or other organ meats?<br><br>গত ২৪ ঘণ্টায় কলিজা, গুর্দা, হৃদপিন্ড বা অন্যান্য অঙ্গের মাংস খেয়েছেন কি?	Yes/হ্যাঁ=1<br>No/না= 2	|___|
**DD09**	Flesh meat	In the last 24 hours, did you eat any beef, mutton, lamb, chicken, duck, other birds etc?<br>গত ২৪ ঘণ্টায় যেকোন ধরনের মাংস (যেমন গরু, খাসী, ভেড়া, মহিষ, হাঁস, মুরগী, কবুতর, ইত্যাদি) খেয়েছেন কি?	Yes/হ্যাঁ=1<br>No/না= 2	|___|
**DD10**	Eggs	In the last 24 hours, did you eat any eggs?<br>গত ২৪ ঘণ্টায় যেকোন ধরনের ডিম খেয়েছেন কি?	Yes/হ্যাঁ=1<br>No/না= 2	|___|
**DD11**	Fish and sea foods	In the last 24 hours, did you eat any fresh or dried fish, or seafood?<br>গত ২৪ ঘণ্টায় যেকোন ধরনের মাছ বা শুঁটকি, সামুদ্রিক খাবার খেয়েছেন কি?	Yes/হ্যাঁ=1<br>No/না= 2	|___|
**DD12**	Legumes, nuts and seeds	In the last 24 hours, did you eat any dried beans, dried peas, lentils, nuts, seeds or food made from thses (e.g. peanut butter)?<br><br>গত ২৪ ঘণ্টায় আপনি শুকনো শিমের বীচি, শুকনো মটর বীজ, ডাল, বাদাম, যে কোনো ধরনের বীজ (যেমন- শিমের বীচি, মটর বীজ) অথবা এগুলো থেকে তৈরি খাবার (যেমন-বাদামের মাখন) খেয়েছেন কি?	Yes/হ্যাঁ=1<br>No/না= 2	|___|
**DD13**	Milk and milk products	In the last 24 hours, did you eat milk, cheese, yogurt, lassi or other milk products?<br>গত ২৪ ঘণ্টায় আপনি দুধ, দই, মাখন, লাচ্ছি অথবা দুধের তৈরি খাবার খেয়েছেন কি?	Yes/হ্যাঁ=1<br>No/না= 2	|___|
**DD14**	Oils and fats	In the last 24 hours, did you eat oil, fats or butter added to food or used for cooking?<br>গত ২৪ ঘণ্টায় আপনি তৈল, চর্বি, মাখন, ডালডা, ঘী অথবা এগুলো থেকে তৈরি যেকোন খাবার খেয়েছেন কি?	Yes/হ্যাঁ=1<br>No/না= 2	|___|
**DD15**	Sweets	In the last 24 hours, did you eat sugar, honey, sweetened juice drinks or sugary food such as chocolates, candies, cookies, cakes etc?<br>গত ২৪ ঘণ্টায় আপনি চিনি, মধু, মিষ্টি	Yes/হ্যাঁ=1<br>No/না= 2	|___|

		জুস, অথবা মিষ্টি জাতীয় খাবার যেমন চকলেট, কেক, বিস্কিট খেয়েছেন কি?		
**DD16**	Spices, condiments, beverages	In the last 24 hours, did you have any spices (black pepper, salt), condiments (soy sauce, hot sauce, chutney, pickles), coffee, tea etc?<br>গত ২৪ ঘণ্টায় আপনি মসলা জাতীয় খাবার, দুধ, চা, কফি, পানীয়, সস, সয়া সস, চাটনি, আচার ইত্যাদি খেয়েছেন কি?	Yes/হ্যাঁ=1<br>No/না= 2	|___|
**IDD**	In the last 24 hours, did you eat anything (meal or snack) outside of the home?<br>গত ২৪ ঘণ্টায় আপনি বাড়ির বাইরে কোন খাবার বা নাস্তা খেয়েছেন কি?		Yes/হ্যাঁ=1<br>No/না= 2	|___|

Menstrutal History

Question number	Questions	Response	Code
**MH1**	Have you entered into your menstrual life?<br>আপনার কি মাসিক শুরু হয়েছে?	Yes/হ্যাঁ=1<br>No/না= 2	|___| If MH1=2, then skip to next section
**MH2**	At what age did you have your first period?<br>আপনার প্রথম মাসিক কত বছর বয়সে হয়েছে?	|___| years	|___|
**MH3**	Are your periods regular? (predictable within one week)<br>আপনার মাসিক কি নিয়মিত হয়? (আপনার ধারণার এক সপ্তাহের মধ্যে)	Yes/হ্যাঁ=1<br>No/না= 2	|___|
**MH4**	How many days of bleeding do you usually have each period? (we mean bleeding for which you needed a tampon or sanitary pad, NOT discharge for which you needed a panty liner only)<br>আপনার প্রত্যেক মাসিকে কতদিন রক্তপাত হয়? (যার জন্য আপনাকে ট্যাম্পুন বা স্যানিটারি ন্যাপকিন ব্যবহার করতে হয়, শুধু প্যান্টি ব্যবহার করলেই হয় না)	|___| days<br>Too irregular to say=888 (এতই অনিয়মিত যে বলার মতো না)	|___|
**MH5**	How heavy is your menstrual flow usually?<br>আপনার রক্ত যাওয়ার পরিমাণ কেমন?	• Light (পাতলা) = 1<br>• Moderate (মধ্যম) = 2<br>• Heavy (clots/flooding) (ভারী - রক্তের চাকা যায়/ অথবা ভেসে যাচ্ছে এমন মনে হয়) = 3<br>• Can't remember (মনে নেই) = 4	|___|
**MH6**	How many days are there between the start of one period and the start of the next on average?<br>আপনার এক মাসিক হতে আরেক মাসিকের মধ্যে গড়ে কতদিনের পার্থক্য থাকে?	• Less than 21 days (<২১দিন) = 1<br>• 22-24 days (২২ – ২৪দিন) = 2	|___|

		○ ays (২৫ - ২৮ দিন) = 3<br>• 29-32 days (২৯ - ৩২ দিন) = 4<br>• 33 – 35 days (৩৩ - ৩৫দিন) = 5<br>• More than 36 days (>৩৬ দিন) = 6<br>• Too irregular to say (এতই অনিয়মিত যে উল্লেখ করার মতো নয়) = 7	
**MH7**	Do you have any of the following symptoms when you have a period?<br>মাসিকের সময় নিম্নের কোন সমস্যাটি আপনার হয়?	• Pelvic pain (pain in the lower party of your belly) (তলপেটে ব্যথা) = 1<br>• Pain on opening your bowels (পায়খানা করার সময় ব্যথা) = 2<br>• Bleeding from your back passage when opening your bowels (পায়খানা করার সময় পায়ুপথে রক্তপাত) = 3<br>• Pain on passing urine<br>• (প্রস্রাবের সময় ব্যথা) = 4<br>• Passing blood in your urine (প্রস্রাবের সাথে রক্তপাত) = 5<br>• Lower back pain<br>• (কোমর ব্যথা) = 6<br>• Pain in upper leg or thighs (পায়ের ঊর্ধাংশে ব্যথা) = 7<br>• Nausea (বমি বমি ভাব) = 8<br>• Tiredness (ক্লান্তি) = 9	|___|
**MH8**	In the last 3 months, have you taken pain-killers for the pain that are prescribed for you by a doctor?<br>গত তিন মাসে আপনি কি চিকিৎসকের পরামর্শ আনুযায়ী কোনো ব্যথানাশক খেয়েছেন কিনা?	Yes/হ্যাঁ=1<br>No/না= 2	|___|
**MH9**	In the last 3 months, have you taken pain-killers for the pain, bought over the counter without prescription?<br>গত তিন মাসে আপনি কি চিকিৎসকের পরামর্শ ছাড়া কোনো ব্যথানাশক খেয়েছেন কিনা?	Yes/হ্যাঁ=1<br>No/না= 2	|___|
**MH10**	In the last 3 months, has your period pain prevented you from going to work or carrying out your daily activities (even if taking pain-killers)?<br>গত তিন মাসে আপনার মাসিকের ব্যথা আপনাকে কাজে যেতে বা দৈনন্দিন কাজ করতে বাধাঁ প্রদান করেছে কিনা?	• Never (কখনই না) = 1<br>• Occasionally (with 1 in 3 of my periods) (মাঝে মাঝে) = 2<br>• Often (with 2 in 3 of my periods) প্রায়ই = 3<br>• Always (with every period) সবসময় = 4	|___|
**MH11**	In the last 3 months, have you had to lie down for any part of the day or longer because of your period pain?<br>গত তিন মাসে আপনার মাসিকের ব্যথার জন্য আপনাকে দিনের কোনো নির্দিষ্ট সময় বা তার চেয়ে বেশি সময় শুয়ে থাকতে হয়েছে কিনা?	• Never (কখনই না) = 1<br>• Occasionally (with 1 in 3 of my periods) (মাঝে মাঝে) = 2<br>• Often (with 2 in 3 of my periods) প্রায়ই = 3	|___|

		• Always (with every period) সবসময় = 4	

	**Health and Nutrition Status**		
**HNS1a**	In the last two weeks, did you suffer from any disease?<br>গত এক সপ্তাহে আপনি কি কোন রুগে ভুগেছেন?	Yes/হ্যাঁ=1<br>No/না= 2	|___|
**HNS1b**	If it is “Yes”, what was the disease?<br>যদি কোনো রোগে ভুগে থাকেন, তাহলে নিম্নের কোন রোগটিতে ভুগেছেন?	• Fever (জ্বর) = 1<br>• Cough / Cold (সর্দি ও কাশি) = 2<br>• Diarrhoea / Dysentery (ডায়রিয়া/আমাশয়) = 3<br>• Stomach ache (পেট ব্যথা) = 4<br>• Respiratory problems (শ্বাস-প্রশ্বাস জনিত সমস্যা) = 5<br>• Ear / eye problems (কান/চোখের সমস্যা) = 6	
**HNS2**	When did you take iron-folic acid tablet for the last time? (Please show IFA tablet and ask the question)<br>আপনি শেষ কবে আয়রন ফলিক এসিড ট্যাবলেট খেয়েছেন? (আয়রন-ফলিক এসিড ট্যাবলেট দেখান, তারপর প্রশ্ন করুন)	|________| days before<br><br>Can’t remember=999<br>Never=888	|___|
**HNS3**	Height of the respondent<br>অংশগ্রহনকারীর উচ্চতা	|___||___| cm	
**HNS4**	Weight of the respondent<br>অংশগ্রহনকারীর ওজন	|___||___| kg	
**HNS5**	BMI of the respondent<br>অংশগ্রহনকারীর বিএমআই	|___||___| kg/m^2	
**HNS6**	MUAC on the left arm<br>বামহাতের মধ্য ও উপরি বাহুর পরিধি	|___||___| cm	
**HNS7**	Hemoglobin concentration<br>হিমোগ্লোবিন এর পরিমান<br><br>(*Instruction:* If Hb<8g/dl, please refer the adolescent girl to a nearby Upazila Health Complex)	|__|__|__|g/dl<br><br>Not taken/refused to give blood sample=888	
	Thank you for participating in the study<br>এই গবেষণা কাজে অংশগ্রহনের জন্য আপনাকে ধন্যবাদ		

Appendix 2: Survey questionnaire for mother or caregiver of the index adolescent girl

TO BE FILLED IN BY FIELD INTERVIEWER

Socio-demographic information

**Ques. No.**	**Questions**	**Responses**	**Code**
**1.**	Name of the mother or caregiver of the index adolescent girl (কিশোরী মেয়ের মায়ের বা সেবাপ্রদানকারীর নাম)		
**2.**	Age of the caregiver (কেয়ারগিভারের বয়স)	|__| |__| Years Months	
**3.**	Occupation of the caregiver (কেয়ারগিভারের পেশা)	• Housewife (গৃহিণী) = 1<br>• Unemployed (বেকার) = 2<br>• Service (চাকুরী) = 3<br>• Business (ব্যবসায়ী) = 4<br>• Farming (কৃষিকাজ) = 5<br>• Others specify অন্যান্য, উল্লেখ করুন_____ = 6	
**4.**	How many years of schooling you completed? (আপনি কত বছর পর্যন্ত পড়াশুনা করেছেন?)	|__||__| years	
**5.**	How many people live in your household? আপনার খানায় সদস্য সংখ্যা কত?	|__||__|	
**6.**	What is the total monthly income of your household? (last one month)<br>আপনার খানার মাসিক আয় কত? (শেষ এক মাস)	|__||__|__|__|__|__|__|Taka	
**7.**	What is the total monthly expenditure of your household? (last one month)<br>আপনার খানার মাসিক ব্যয় কত? (শেষ এক মাস)	|__||__|__|__|__|__|__| Taka	
**8.**	Head of the household? (পরিবারের প্রধান কে?)	Male (পুরুষ) = 1<br>Female (মহিলা) = 2	
**9.**	How many years of schooling the household head completed?<br>(পবিবার প্রধানের শিক্ষাগত যোগ্যতা কি?)	|__||__| years	
**10.**	Occupation of the household head (পরিবার প্রধানের পেশা কি?)	• Unemployed (বেকার) = **1**<br>• Service (চাকুরী) = 2<br>• Business (ব্যবসায়ী) = 3<br>• Farming (কৃষিকাজ) = 4<br>• Others specify অন্যান্য, উল্লেখ করুন_____ = 5	
**11.**	Is the respondent biological mother of the index girl (উত্তরদাতা কি আমাদের তালিকাভুক্ত মেয়ের মা?)	Yes/হ্যাঁ=1<br>No/না= 2	
**12.**	Father of the index girl alive (আমাদের তালিকাভুক্ত মেয়ের বাবা কি বেচেঁ আছেন?)	Yes/হ্যাঁ=1<br>No/না= 2	
**13.**	Education level of the father of the index girl (আমাদের তালিকাভুক্ত মেয়ের বাবার শিক্ষাগত যোগ্যতা কি?)	|__||__| years	
**14.**	Age of the father (বাবার বয়স)	|__| |__| Years Months	

15.	Production of vegetables by household<br>(খানা কর্তৃক শাকসবজি উৎপাদন)	Yes/হ্যাঁ=1<br>No/না= 2	

Assets Data

Ques. No.	Questions	Responses	Code
**WI1**	Does your household have electricity?<br>(আপনার খানায় কি ইলেক্ট্রিসিটি আছে?)	Yes/হ্যাঁ = 1<br>No/না = 2	
**WI2**	What fuels does your household use for cooking?<br>(আপনার খানায় রান্নার কাজে কি কি জ্বালানি ব্যবহৃত হয়?)	• Electricity (ইলেক্ট্রিসিটি) = 1<br>• Gas (গ্যাস) = 2<br>• Kerosene (কেরোসিন) = 3<br>• Biogas (Gobar gas) (বায়োগ্যাস (গোবর গ্যাস) = 4<br>• Solar (সোলার) = 5<br>• Coal lignite (কয়লা/লিগনিট) = 6<br>• Charcoal (কাঠকয়লা/ চারকোল) = 7<br>• Wood/Bamboo (কাঠ/বাঁশ) = 8<br>• Straw/shrubs/grass/crop residue (খড়কুটা/ কুড়ানো পাতা/ ঘাস/ফসলের অবশিষ্টাংশ) = 9<br>• Animal dung (প্রানীর গোবর) = 10<br>• Other, If Other, specify: __________অন্যান্য ,যদি অন্যান্য হয়, তাহলে নির্দিষ্ট করুন:___________ = 11	
**WI3**	Main material of the floor of the dwelling?<br>(আপনার বসতঘরের মেঝে প্রধানত কিসের তৈরি?)	• Dirt/earth/sand/dung / mixed (ধুলা/ মাটি/বালু/ গোবর/মিশ্রিত) = 1<br>• Other (tiles, concrete, wood) (অন্যান্য (টাইলস, ইট, কাঠ)) = 2	
**WI4**	Does your household have...?<br>(খানায় উল্লেখিত জিনিসগুলোর মধ্যে কোন কোনটি আছে?)	• Atleast 15 decimal cultivable Land?<br>• (অন্তত পক্ষে ১৫ শতাংশ কৃষি জমি?) -----------------1<br>• A television? (একটি টেলিভিশন?)------------------2<br>• A mobile or non-mobile telephone? (একটি মোবাইল বা টেলিফোন?) ------------------------------3<br>• Cattle/poultry (গবাদি পশু/ পোল্ট্রী)----------------4<br>• A motorcycle, bike, scooter, rickshaw, rickshaw-van, boat with engine?<br>• (মটরসাইকেল, বাই-সাইকেল, স্কুটার, রিক্সা,ভ্যান, ইঞ্জিনচালিত নৌকা?)----------------------------------5<br>• A car, truck, jeep, or tractor? (গাড়ি, ট্রাক, জিপ, বা ট্রাক্টর?) --6<br>• A refrigerator? (রেফ্রিজেরেটর/ফ্রিজ?) -------------7<br>• A computer /laptop /tablet (কম্পিউটার/ল্যাপটপ/ ট্যাবলেট-পিসি) ---------------------------------------8<br>• A horse /cow /donkey /buffalo cart? (ঘোড়া/গরু/মহিষ/গাধার গাড়ি?) ----------------------9<br>• Furniture and related items (আসবাবপত্র বা এ জাতীয় জিনিস?) --- 10<br>• A mattress? (ম্যাট্রেস?) ……………………11<br>• A chair (চেয়ার) ……………………………………13	

		• A table (টেবিল)…………………………………...14<br>• Agro-machineries (কৃষিজ যন্ত্রপাতি?) --------------- 15<br>• Other, If Other, specify: _________অন্যান্য, যদি অন্যান্য হয়, তাহলে নির্দিষ্ট করুন:________----------17	
**WI5**	Do your household has separate kitchen (আপনাদের কি আলাদা রান্নাঘর আছে)?	Yes/হ্যাঁ = 1<br>No/না = 2	
**WI6**	How many living rooms do you have in your household (আপনাদের খানায় কয়টি বসবাসের ঘর আছে)?	------------------	

Water, Sanitation, and Hygiene (WASH)

Ques. No.	Questions	Responses	Code
**W1**	What is the main source of drinking water for you and your girl?<br>(আপনার এবং আপনার শিশুর জন্য খাবার পানির প্রধান উৎস কি?)	• Tap water (ট্যাপের পানি) -------------------------- = 1<br>• Tube-well (টিউবওয়েল/টানা কল) ---------------= 2<br>• Protected dug well/ spring/ Rainwater (সংরক্ষিত কুয়া/ঝর্ণা/বৃষ্টির পানি) ----------------------------= 3<br>• Bottled water / sachet water (বোতলজাত পানি/প্যাকেটজাত পানি) ------------------------- = 4<br>• Unprotected dug well/spring (অসংরক্ষিত কুয়া/ঝর্ণা) -------------------------------------- = 5<br>• River or stream (নদী বা জলস্রোত) ------------ = 6<br>• Dam, lake, or pond, canal or irrigation channel (বাঁধ, হ্রদ বা পুকুর, খাল বা সেচ প্রণালী) --------- = 7<br>• Other unprotected sources of drinking water (অন্যান্য অসংরক্ষিত খাবার পানির উৎস) ------- = 8<br>• Other, If Other, specify: _______ (অন্যান্য,যদি অন্যান্য হয়, তাহলে নির্দিষ্ট করুন:_______) ---------------------------------- = 9	
**W2**	What do you do to the water to make it safer to drink?<br>(খাবার পানিকে নিরাপদ ও পানযোগ্য করার জন্য আপনি কি করেন?)	• Boil (পানি ফুটানোর মাধ্যমে) --------------------= 1<br>• Add bleach / chlorine tablet (ব্লীচিং পাউডার/ক্লোরিন ট্যাবলেট মিশাই) ---------------= 2<br>• Use a water filter / gravel /ceramic / sand (ফিল্টার/নুড়িপাথর/সিরামিক/ বালি ব্যবহার করে পানি পরিশোধিত করা) -------------------------------- = 3<br>• Solar disinfection (সূর্যের আলো ও তাপ দিয়ে সংক্রমণ মুক্ত করি) ----------------------------- = 4<br>• Strain it through a cloth **only** (শুধু কাপড় দিয়ে পরিশ্রাবণ করি/ছাঁকি) -------------------------- = 5<br>• Let it stand and settle **only** (শুধু স্থির ও থিতু হতে দেই) -- = 6<br>• Nothing (কোন কিছুই করি না) ------------------- = 7<br>• Don't know (জানি না) -------------------------- = 8<br>• Other, If Other, specify: ________ (অন্যান্য,যদি অন্যান্য হয়, তাহলে নির্দিষ্ট করুন:______) ------------------- = 9	
**W3**	Please observe, Did the point of water	Yes / হ্যা = 1	

	source (for drinking) look clean?<br>(পানির উৎসের স্থানটি দেখতে পরিষ্কার কি না?)<br>Note: Clean means no water logging, no feces besides, no dirt besides etc<br>নোটঃ পরিষ্কার অর্থ স্থানটিতে পানি জমে থাকবে না, চারপাশে পায়খানা বা অন্য কোন ময়লা পড়ে থাকবে না।)	No/ না = 2<br>Far away / অনেক দূরে = 3	
**S1**	What kind of toilet facility do members of your household usually use?<br>(আপনার খানার লোকজন সচরাচর/সাধারণত কি ধরনের পায়খানা ব্যবহার করে থাকেন?)	• Flush or pour flush to (ফ্লাশ করা বা স্রোতের ন্যায় ফ্লাশ করা) -- 1<br>• Piped sewer system (স্যুয়ারেজ লাইনের সাথে যুক্ত পায়খানা) ------------- 2<br>• Septic tank (স্যাপ্টিক ট্যাংক) -----------------------3<br>• Pit latrine (পিট পায়খানা) -------------------------- 4<br>• KVIP latrine (কেভিআইপি পায়খানা) -------------- 5<br>• Pit latrine with slab (স্ল্যাবসহ পিট পায়খানা) ------ 6<br>• Composting toilet (টয়লেটকে মিশ্রসারে পরিনত করা) --- 7<br>• Elsewhere (not specified above) ------------------- 8 অন্যত্র (উপরে উল্লেখিত নয় এমন)<br>• Pit latrine without slab (স্ল্যাবছাড়া খোলা পিট) ---- 9<br>• Bucket (বালতি লাগানো পায়খানা) ---------------- 10<br>• Hanging latrine (ঝুলন্ত পায়খানা) ---------------- 11<br>• Bush or field No facilities (খোলা মাঠ বা ঝোঁপ-ঝাড় বা ছোট বন-জঙ্গল পায়খানা নেই/সুযোগ সুবিধা নেই) --- 12<br>• Other; If Other, specify: ________<br>• অন্যান্য ; যদি অন্যান্য হয়, তাহলে নির্দিষ্ট করুন:______ ------------------------------------ 13	
**S2**	Do you share this facility with other households?<br>(আপনি কি অন্যের সাথে/অন্য পরিবারের সাথে পায়খানা শেয়ার করেন/ভাগাভাগি করে ব্যবহার করেন?)	Yes / হ্যা = 1<br>No/ না = 2<br>Not Applicable/ প্রযোজ্য নয় = 777	
**H1**	Observe presence of water at the specific place for hand washing.<br>(Verify by checking the tap/pump, or basin, bucket, water container or similar objects for presence of water)<br>(হাত ধৌত করার স্থানে পানি আছে কি না পর্যবেক্ষন করুন। (পানির কল, পাম্প, বেসিন, বালতি, অন্য কোন পানির পাত্রে পানি আছে কিনা যাচাই করুন)	Yes / হ্যা = 1<br>No/ না = 2<br>No permission to see<br>পর্যবেক্ষন করতে অনুমতি দেয় নাই = 3	
**H2**	Record if soap or detergent or anything is present at the specific place for hand washing.<br>(হাত ধৌত করার নির্দিষ্ট স্থানে সাবান অথবা ডিটার্জেন্ট বা অন্যকিছু থাকলে লিপিবদ্ধ করুন)	• Bar soap সাবান -------------------------------------- 1<br>• Detergent (Powder / Liquid / Paste) ডিটার্জেন্ট (গুড়া/ তরল/পেস্ট) ---------------------------------- 2<br>• Liquid soap (তরল সাবান) --------------------------3<br>• Ash/Mud/Sand (ছাই/ মাটি /বালু) -------------------4<br>• None কিছুই না	
**H3**	Request the mother/caregiver to show	Yes / হ্যা = 1	

	soap or detergent she generally uses for handwashing at the critical moments. Was the mother/caregiver able to show the soap/detergent (within one minute)?<br>(মা/প্রধান লালন-পালনকারীর হাত ধোয়ার গুরুত্বপূর্ণ মূহুর্তে যে সাবান বা ডিটার্জেন্ট ব্যবহার করেন সেটা কি তিনি এক মিনিটের মধ্যে দেখাতে পেরেছেন?)	No/ না = 2	
**H4**	Have you ever washed both hands yesterday from this time until today now?<br>(গতকাল এই সময় থেকে এখন পর্যন্ত আপনি কি আপনার দুহাত ধৌত করেছিলেন?)	Yes / হ্যা = 1<br>No/ না = 2	
**H5**	How many times you washed both hands by yesterday from this time until today now?<br>(গতকাল এই সময় থেকে এখন পর্যন্ত আপনি কতবার আপনার দুহাত ধৌত করেছিলেন?)	times/ বার<br>(if none put '00')<br>(যদি কখনো না হয় তাহলে "00" বসান)	
**H6**	Yesterday from this time until today now on what occasions did you wash both hands? (multiple responses allowed)<br>(গতকাল এই সময় থেকে এখন পর্যন্ত কি কি কাজে আপনি দুহাত ধৌত করেছিলেন?)	• Washing child's bottoms<br>(শিশুকে শৌচ করানোর পর)---------------------- = 1<br>• Washing my children's hands<br>(শিশুর হাত ধোয়ানোর পর) ----------------------- = 2<br>• Washing hands after defecating<br>(শৌচ কাজের পর হাত ধোয়ার জন্য) -------------- =3<br>• Washing hands before feeding child<br>(শিশুকে খাওয়ানোর আগে হাত ধোয়ার জন্য)------ = 4<br>• Before preparing food<br>(খাদ্য তৈরির আগে) ---------------------------- = 5<br>• Before cutting fruits/preparing salad<br>(ফল কাটা /সালাদ তৈরির আগে) ---------------- = 6<br>• Before eating<br>(খাওয়ার আগে) --------------------------------- = 7<br>• After disposing off dirt and garbage<br>(ময়লা-আবর্জনা পরিষ্কার করার পর)------------ = 8<br>• After washing utensils<br>(থালা-বাসন ধৌত করার পর) -------------------- = 9<br>• After cooking (রান্নার পর) --------------------= 10<br>• During bath (গোসলের সময়) -----------------= 11<br>• Before prayer<br>(প্রার্থনার (নামাজ/পূজা বা অন্যান্য) আগে) -----= 12<br>• After washing cloth<br>(কাপড় ধৌত করার পর) ---------------------- = 13<br>• After cleaning animal faeces<br>(প্রাণীর গোবর বা ময়লা পরিষ্কার করার পর) ---- = 14<br>• After eating (খাওয়ার পর) ------------------- = 15<br>• During child bath<br>(শিশুকে গোসল করানোর সময়) --------------- = 16<br>• During face and hand wash<br>(হাত-মুখ ধৌত করার সময়) ------------------- = 17<br>• After wake up<br>(ঘুম থেকে উঠে) ---------------------------- = 18<br>• Other; If Other, specify: ______	

		• অন্যান্য; যদি অন্যান্য হয়, তাহলে নির্দিষ্ট করুনঃ ______ -------------------------- = 19	
**H7**	Have you used soap for anywork yesterday from this time until today now? (আপনি কি গতকাল এই সময় থেকে এখন পর্যন্ত কোন কাজে সাবান ব্যবহার করেছিলেন?)	Yes / হ্যা = 1<br>No/ না = 2	
**H8**	Yesterday from this time until today now on what occasions did you use soap? (multiple responses allowed)<br>(গতকাল এই সময় থেকে এখন পর্যন্ত কি কি কাজে সাবান ব্যবহার করেছিলেন?)	• Washing cloths<br>কাপড় ধোয়ার সময় ----------------------------- = 1<br>• Washing my body<br>নিজের গোছলের জন্য --------------------------- = 2<br>• Washing my Children<br>শিশুকে গোছল করানোর সময় ----------------- = 3<br>• Washing child's bottoms<br>শিশুকে শৌচ করানোর পর --------------------- = 4<br>• Washing my children's hands<br>শিশুর হাত ধোয়ানোর জন্য ------------- = 5<br>• Washing hands after defecating<br>শৌচ কাজের পর হাত ধোয়ার জন্য ------------- = 6<br>• Washing hands before feeding child<br>শিশুকে খাওয়ানোর আগে হাত ধোয়ার জন্য ----- = 7<br>• Washing hands before preparing food<br>খাদ্য তৈরির আগে হাত ধোয়ার জন্য ----------------- = 8<br>• Washing hands before eating<br>খাওয়ার আগে হাত ধোয়া ------------------------- = 9<br>• Washing hands after cleaning dust<br>ময়লা –আবর্জনা পরিষ্কারের পর হাত ধোয়ার জন্য = 10<br>• Washing hands after cooking<br>রান্নার পর হাত ধোয়ার জন্য ---------------------- = 11<br>• Washing hands after cleaning utensils<br>থালা-বাসন ধোয়ার পর হাত ধোয়ার জন্য --------- = 12<br>• Washing hands after touching or cleaning animal faeces (প্রাণীর গোবর বা ময়লা পরিষ্কারের পর হাত ধোয়ার জন্য) ----------------------------------- = 13<br>• Washing face and hands<br>হাত মুখ ধোয়ার জন্য --------------------------- = 14<br>• Washing utensils<br>থালা-বাসন ধোয়ার জন্য ------------------------ = 15<br>• Other; If Other, specify: ______<br>অন্যান্য; যদি অন্যান্য হয়, তাহলে নির্দিষ্ট করুন___________ ----------------- = 16	

**H9**	Please tell me all of the occasions when it is important for you to wash hands with soap<br>(কোন কোন ক্ষেত্রে সাবান দিয়ে হাত ধোয়া বিষয়টিকে আপনার কাছে জরুরি বলে মনে হয়, অনুগ্রহ করে সব ধরনের বিষয় / ক্ষেত্রগুলো বলুন)	• Before eating<br>খাওয়ার আগে --------------------------------------- = 1<br>• Before feeding a child<br>শিশুকে খাওয়ানোর পূর্বে -------------------------= 2<br>• Before cooking /preparing/serving food<br>রান্না করা/খাবার তৈরির/পরিবেশনের আগে ----- = 3<br>• After defecation/ urination পায়খানা করার পর = 4<br>• After cleaning a child that has defecated /changing nappies/ washing diaper<br>শিশুকে শৌচ করানোর পর/শিশুর ন্যাপকিন বদলানো পর/ ডায়পার ধৌত করার পর --------------------- 5<br>• After disposing off dirt and garbage<br>ময়লা – আবর্জনা পরিষ্কার করার পর ------------- 6<br>• After any work<br>যে কোন কাজের পর --------------------------------- 7<br>• During bath<br>গোসলের সময় ---------------------- 8<br>• After cleaning animal dung<br>গোবর পরিষ্কার করার পর ----------------------- 9<br>• After cooking রান্নার পর -------------------------- 10<br>• After eating খাবার পর ....11<br>• Other; If Other, specify: ________<br>• অন্যান্য; যদি অন্যান্য হয়, তাহলে নির্দিষ্ট করুন:________ ---------------------12	

**Household Dietary Diversity**				
গতকাল এই সময় থেকে (সাক্ষাৎকার শুরু করার সময় থেকে বিবেচনা করে) এখন পর্যন্ত কি কি খেয়েছে?<br>**Probe** করুনঃ আর কিছু<br>***Tick or cross ALL boxes!***<br>*টিক চিহ্ন দিন*				
**Question number**	**Food Group**	**Question (with example)**	**Response**	**Code**
**DD01**	Cereals	In the last 24 hours, did you eat any food made from grain such as millet, wheat, rice, maize, semolina, atta=flour, noodles, porridge, jau?<br>গত ২৪ ঘণ্টায় শস্যদানা থেকে প্রাপ্ত খাদ্য যেমন ভুট্টা, ধান বা গমের আটা, ময়দা, চাল/ভাত, চিড়া, মুড়ি, রুটি, পাউরুটি এবং অন্যান্য শস্যজাত খাবার, জাও, সুজি খেয়েছেন কি?	Yes/হ্যাঁ=1<br>No<br>/না= 2	|___|
**DD02**	White roots and tubers	In the last 24 hours, did you eat any food made from roots or tubers such as white potato, white yam, radish, turnip, wol etc?<br>গত ২৪ ঘণ্টায় গাছের মুল বা কান্ড যেমন আলু, কেশর আলু, সাদা আলু, কচু, মূলা, শালগম, কেশর আলু ,ওল থেকে প্রস্তুতকৃত খাদ্য খেয়েছেন কি?	Yes/হ্যাঁ=1<br>No/না= 2	|___|
**DD03**	Vitamin A rich vegetables and tubers	In the last 24 hours, did you eat any food made from pumpkin, carrot, sweet potato, vegetables that have yellow or orange flesh etc?<br>গত ২৪ ঘণ্টায় লাউ, কুমড়া, মিষ্টি কুমড়া, গাজর, শসা এরকম লাল বা হলুদ শাকসবজি খেয়েছেন কি?	Yes/হ্যাঁ=1<br>No/না= 2	|___|
**DD04**	Dark green leafy vegetables	In the last 24 hours, did you eat any food made from any dark green leafy vegetables such as palang, puishak, mula shak, pat shak, sarso, spring onion and radish leaves, etc?<br>গত ২৪ ঘণ্টায় যেকোনো গাঢ় সবুজ শাকসবজি যেমন পালং শাক, পুঁই শাক, পাট শাক, মুলা শাক, ধনে পাতা, পিয়াজ শাক ইত্যাদি খেয়েছেন কি?	Yes/হ্যাঁ=1<br>No/না= 2	|___|
**DD05**	Other vegetables	In the last 24 hours, did you eat any other vegetables like tomatoes, eggplant, onion, cauliflower, cabbage etc?<br>গত ২৪ ঘণ্টায় অন্যান্য সবজি যেমন টমেটো, বেগুন, পেঁয়াজ ইত্যাদি খেয়েছেন কি?	Yes/হ্যাঁ=1<br>No/না= 2	|___|
**DD06**	Vetamin A rich fruits	In the last 24 hours, did you eat any ripe mango, ripe papaya, cantaloupe, watermelon etc and juice from these fruits?<br>গত ২৪ ঘণ্টায় পাকা আম, পাকা পেঁপে, তরমুজ, খরমুজ ইত্যাদি এবং এগুলোর জুস খেয়েছেন কি?	Yes/হ্যাঁ=1<br>No/না= 2	|___|
**DD07**	Other fruits	In the last 24 hours, did you eat any fruits like apple, green mango, banana, pomegranate, lemon, guava, jackfruit, pineapple, pawpaw, orange, lychee, plum, black berries, dates, palms, dalims, kamranga, jambura, latkon, bel	Yes/হ্যাঁ=1<br>No/না= 2	|___|

		etc and juice from these fruits?<br>গত ২৪ ঘণ্টায় আপেল, কাঁচা আম, কলা, লেবু, পেয়ারা, কাঁঠাল, ডালিম, পেঁপে, আনারস, বরই, কালো জাম, কামরাঙ্গা, জাম্বুরা, কমলা, বেল, লটকন, তাল, লিঁচু, ইত্যাদি এবং এগুলোর জুস খেয়েছেন কি?		
**DD08**	Organ meat	In the last 24 hours, did you eat any liver, kidney, heart or other organ meats?<br><br>গত ২৪ ঘণ্টায় কলিজা, গুর্দা, হৃদপিন্ড বা অন্যান্য অঙ্গের মাংস খেয়েছেন কি?	Yes/হ্যাঁ=1<br>No/না= 2	|___|
**DD09**	Flesh meat	In the last 24 hours, did you eat any beef, mutton, lamb, chicken, duck, other birds etc?<br>গত ২৪ ঘণ্টায় যেকোন ধরনের মাংস (যেমন গরু, খাসী, ভেড়া, মহিষ, হাঁস, মুরগী, কবুতর, ইত্যাদি) খেয়েছেন কি?	Yes/হ্যাঁ=1<br>No/না= 2	|___|
**DD10**	Eggs	In the last 24 hours, did you eat any eggs?<br>গত ২৪ ঘণ্টায় যেকোন ধরনের ডিম খেয়েছেন কি?	Yes/হ্যাঁ=1<br>No/না= 2	|___|
**DD11**	Fish and sea foods	In the last 24 hours, did you eat any fresh or dried fish, or seafood?<br>গত ২৪ ঘণ্টায় যেকোন ধরনের মাছ বা শুঁটকি, সামুদ্রিক খাবার খেয়েছেন কি?	Yes/হ্যাঁ=1<br>No/না= 2	|___|
**DD12**	Legumes, nuts and seeds	In the last 24 hours, did you eat any dried beans, dried peas, lentils, nuts, seeds or food made from thses (e.g. peanut butter)?<br><br>গত ২৪ ঘণ্টায় আপনি শুকনো শিমের বীচি, শুকনো মটর বীজ, ডাল, বাদাম, যে কোনো ধরনের বীজ (যেমন- শিমের বীচি, মটর বীজ) অথবা এগুলো থেকে তৈরি খাবার (যেমন-বাদামের মাখন) খেয়েছেন কি?	Yes/হ্যাঁ=1<br>No/না= 2	|___|
**DD13**	Milk and milk products	In the last 24 hours, did you eat milk, cheese, yogurt, lassi or other milk products?<br>গত ২৪ ঘণ্টায় আপনি দুধ, দই, মাখন, লাচ্ছি অথবা দুধের তৈরি খাবার খেয়েছেন কি?	Yes/হ্যাঁ=1<br>No/না= 2	|___|
**DD14**	Oils and fats	In the last 24 hours, did you eat oil, fats or butter added to food or used for cooking?<br>গত ২৪ ঘণ্টায় আপনি তৈল, চর্বি, মাখন, ডালডা, ঘী অথবা এগুলো থেকে তৈরি যেকোন খাবার খেয়েছেন কি?	Yes/হ্যাঁ=1<br>No/না= 2	|___|
**DD15**	Sweets	In the last 24 hours, did you eat sugar, honey, sweetened juice drinks or sugary food such as chocolates, candies, cookies, cakes etc?<br>গত ২৪ ঘণ্টায় আপনি চিনি, মধু, মিষ্টি জুস, অথবা মিষ্টি জাতীয় খাবার যেমন চকলেট, কেক, বিস্কিট খেয়েছেন কি?	Yes/হ্যাঁ=1<br>No/না= 2	|___|
**DD16**	Spices, condiments, beverages	In the last 24 hours, did you have any spices (black pepper, salt), condiments (soy sauce, hot sauce, chutney, pickles), coffee, tea etc?<br>গত ২৪ ঘণ্টায় আপনি মসলা জাতীয় খাবার, দুধ, চা, কফি, পানীয়, সস, সয়া সস, চাটনি, আচার ইত্যাদি খেয়েছেন কি?	Yes/হ্যাঁ=1<br>No/না= 2	|___|

**HDD**	In the last 24 hours, did you eat anything (meal or snack) outside of the home?<br>গত ২৪ ঘণ্টায় আপনি বা আপনার বাড়ির কেউ বাড়ির বাইরে কোন খাবার বা নাস্তা খেয়েছেন কি?	Yes/হ্যাঁ=1<br>No/না= 2	|___|

Appendix 3: Guideline for In-depth interview with the adolescent girl (কিশোরী মেয়েদের নিবিড় সাক্ষাৎকার গ্রহণের জন্য গাইডলাইন)

1. **Basic information** (মৌলিক তথ্য) **:** Name, age, religion, residence type, educational status of the parents, occupation of parents (নাম,বয়স , ধর্ম ,বাসস্থানের ধরণ, পিতামাতার শিক্ষাগত যোগ্যতা , পিতামাতার পেশা ইত্যাদি)

2. **Decision making on food selection (খ্যাদ্য নির্বাচনের সিদ্ধান্ত) :**

   a. How decision is made to select/buy food for the household? (Probe: Who made the decision? How frequently food is bought / collected? Why, Who buys/collects etc) (পরিবারে কি কি খাবার কিনতে হবে বা লাগবে সে বিষয়ে কিভাবে সিদ্ধান্ত নেয়া হয়?; প্রোব করুন: কে সিদ্ধান্ত নেয় ? কে কিনে? কতদিন পর পর কেনা হয়? ইত্যাদি)
   b. What types of foods are frequently bought for your household? (Probe: based on food group) (আপনাদের পরিবারের জন্য কোন ধরণের খাদ্য প্রায়ই কেনা হয় ?; খ্যাদ্য গ্রূপ অনুযায়ী প্রোব করুন)
   c. Do you ask him/her (who frequently bought) to bring any food based on your preference? (Probe: what type of food you prefer to tell them to buy frequently? Why do you prefer that/those foods?, how does he/she react when you ask to bring those food? etc) (যিনি আপনাদের পরিবারের জন্য খাবার কিনেন, তাকে কি আপনি আপনার পছন্দের খাবার আনতে বলতে পারেন ? প্রোব করুন : কোন ধরনের খাবার আপনি আনতে ? কেন আপনি ঐ ধরণের খাবার পছন্দ করেন? আপনার পছন্দের খাবার আনতে বললে তিনি কি করেন ?)

   d. How does the preference of buyer (who generally buys food for your household) influence food selection from market? (যিনি আপনাদের পরিবারের জন্য খাবার কিনেন, তিনি কোন কোন ধরণের খাবার কিনবেন সেটা তার পছন্দের উপর কতটা নির্ভর করে ?)

3. **Food preparation (খাবার তৈরী:**
   a. Who usually prepare food for your household (How frequently food is prepared? why? How many food dishes are generally prepared for each meal?) (আপনাদের পরিবারের লোকজনের জন্য সাধারণতঃ কে খাবার তৈরী করেন ? প্রোব করুন: কয় বেলা খাবার তৈরী করা হয় ? কেন ? প্রতি বেলা খাবারে কয় প্রকারের খাবার তৈরী করা হয়?)

   b. Do you have involvement in food preparation? (Probe: how frequently you prepare food? which food you frequently prepare? Why? Can you prepare the food that you prefare to take? Why or why not?) (আপনি কি খাবার তৈরী করেন ? প্রোব করুন: কতটা নিয়মিত খাবার তৈরী করেন ? কোন ধরণের খাবার বেশি তৈরী করেন ? কেন ? যে খাবার আপনি খেতে পছন্দ করেন সেই খাবার কি আপনি তৈরী করতে পারেন? কেন অথবা কেন না ?)

   c. Do you have any practice to add sour fruits like unripe mangoes, olive etc to whole grain cereals, legumes etc? When and how do you do this practice (probe: in any particular session, during menstruation etc)?

আপনার বা আপনার পরিবারের কোনো সদস্যদের কি কোনো শস্য জাতীয় খাবার বা ডাল জাতীয় খাবারের সাথে কাঁচা আম, জলপাই ইত্যাদি টক ফল খাওয়ার অভ্যাস আছে? কখন এবং কিভাবে তারা এগুলো খায়? (প্রোব করুন: তারা কি বছরের কোনো নির্দিষ্ট ঋতুতে এই ধরণের খাবার খায়? মাসিকের সাথে এই ধরণের খাবার খাওয়ার কোনো সম্পর্ক আছে কীনা?)

d. Do you have any practice to add chutney or pickles to your main meals like lunch or dinner? When and how do you do this practice (probe: in any particular session, during menstruation etc)?

   আপনার বা আপনার পরিবারের কোনো সদস্যদের কি প্রধান খাবার যেমন দুপুরের বা রাতের খাবারের সাথে আচার বা চাটনি খাবার অভ্যাস আছে? কখন এবং কিভাবে তারা এগুলো খায়? (প্রোব করুন: তারা কি বছরের কোনো নির্দিষ্ট ঋতুতে এই ধরণের খাবার খায়? মাসিকের সময় এইসব খাবার খাওয়া?)

e. Do you have any practice such as germination (chickpea), fermentation (yogurt, sweet yogurt, bread). How do you prepare those?

   আপনার বা আপনার পরিবারের সদস্যদের কি কোনো খাবার (যেমন ছোলা) পানিতে ভিজিয়ে রেখে , বা কোন খাবার গাঁজন (যেমন টক দই, মিষ্টি দই, পাউরুটি ইত্যাদি) করে খাওয়ার অভ্যাস আছে? আপনারা কিভাবে তৈরী করেন?

4. **Diets of the adolescent:** (কিশোরীদের খাবার)

Breakfast (সকালের খাবার)

a. What types of food you take in your breakfast? (Probe: are these foods generally home made or brought from outside? Do you select these foods by your self or someone else chooses for you? (If she skips breakfast) How frequently you skip breakfast and why?)
   কি ধরণের খাবার আপনি সকালের নাস্তায় খান? (প্রোব করুন: এই খাবারগুলো কি বাসায় বানানো হয় নাকি বাইরে থেকে আনা হয় ? আপনি কি এই খাবারগুলো নিজেই নির্বাচন করেন নাকি অন্য কেউ নির্বাচন করে? (যদি সে সকালের নাস্তা না খায়) আপনি কি প্রায়ই সকালের নাস্তা খাওয়া বাদ দেন? বাদ দিলে সেই বাদ দেয়ার ধরণটা কেমন? এবং কেন বাদ দিয়েছেন?)

b. What types of foods you like to take in breakfast and why?
   আপনি কি ধরণের খাবার খেতে সকালের নাস্তায় পছন্দ করেন? এবং কেন?

c. What types of food you dislike to take in breakfast and why?
   আপনি কি ধরণের খাবার খেতে সকালের নাস্তায় অপছন্দ করেন? এবং কেন?

d. Do you face any difficulties in taking breakfast? (Probe: health related difficulties, foods you are served but you don't like etc)
   আপনি কি সকালের নাস্তা খাবারের ব্যাপারে কোনো ধরণের সমস্যার সম্মুখীন হন? (প্রোব করুন: স্বাস্থ্যগত সমস্যা, যে খাবারগুলো আপনাকে খেতে দেয়া হয়েছিল সেগুলো আপনার পছন্দ হয় নি)

e. Is there any food item that you are told not to take at breakfast? (prob: by your family members or relatives or peers or neighbor)
   এমন কি কোনো খাবার আছে যা আপনাকে সকালের খাবার হিসেবে খেতে নিষেধ করা হয়েছে? (যদি নিষেধ করে থাকে তবে কে করেছে ? পরিবারের কি কোনো সদস্য/ আত্মীয়-স্বজন / কোনো বন্ধু-বান্ধব/ পাড়া-প্রতিবেশী?)

   Lunch (দুপুরের খাবার)

a. What types of foods you like to take in your lunch and why? (Probe: In week days and in weekend? Is the lunch of your home generally home made or brought from outside? Do you select these foods by your self or someone else chooses for you?)
কি ধরণের খাবার আপনি দুপুরের খাবার হিসেবে খেতে পছন্দ করেন এবং কেন?
(প্রোব করুন: ছুটির দিনে এবং কর্মদিবসের দুপুরের খাবারে কোনো পার্থক্য আছে কিনা? এই খাবারগুলো কি বাসায় বানানো হয় নাকি বাইরে থেকে আনা হয়? আপনি কি এই খাবারগুলো নিজেই নির্বাচন করেন নাকি অন্য কেউ নির্বাচন করে?

b. In week days, how do you manage your lunch? (Prob: home made, bought from outside)
কর্মদিবসে (যেদিন আপনার স্কুল থাকে) আপনি আপনার দুপুরের খাবারের ব্যবস্থা কিভাবে করেন? (প্রোব করুন: খাবারগুলো বাসায় বানানো হয় নাকি বাইরে থেকে কিনে আনা হয়?)

c. Do you skip luch? If so, how frequently you skip? Why do you skip lunch?
আপনি কি দুপুরের খাবার বাদ দেন ? (যদি দেন, আপনার বাদ দেয়ার ধরণটা কেমন এবং কেন বাদ দেন?)

d. Is there any food item that you are told not to take at lunch? (prob: by your family members or relatives or peers or neighbor)
এমন কি কোনো খাবার আছে যা আপনাকে দুপুরের খাবার হিসেবে খেতে নিষেধ করা হয়েছে? (যদি নিষেধ করে থাকে তবে কে করেছে ? পরিবারের কি কোনো সদস্য/ আত্মীয় –স্বজন/বন্ধু-বান্ধব/ পাড়া-প্রতিবেশী?)

Dinner (রাতের খাবার)

a. What types of foods you like to take in dinner and why?
কোন ধরণের খাবার আপনি রাতের খাবার হিসেবে খেতে পছন্দ করেন এবং কেন ?

b. Do you skip dinner? If so, how frequently you skip? Why do you skip dinner?
আপনি কি রাতের খাবার বাদ দেন ? (যদি দেন, আপনার বাদ দেয়ার ধরণটা কেমন এবং কেন বাদ দেন?)

c. Is there any food item that you are told not to take at dinner? (prob: by your family members or peers or neighbors)
এমন কি কোনো খাবার আছে যা আপনাকে দুপুরের খাবার হিসেবে খেতে নিষেধ করা হয়েছে? (যদি নিষেধ করে থাকে তবে কে করেছে ? পরিবারের কি কোনো সদস্য/ কোনো বন্ধু-বান্ধব/ পাড়া-প্রতিবেশী?)

Extra meal

a. Do you take any extra meal? If yes, what types of food (Probe: How frequently you take?, what type of food is this? Homemade or brought from outside? influenced by peer or relatives, neighbour or family members to those foods?)
বাড়তি নাস্তা আপনি কি সকাল, দুপুর ও রাতের খাবার ছাড়াও বাড়তি কোনো খাবার খান? যদি খেয়ে থাকেন, কি ধরণের খাবার খান? (প্রোব করুন: বাড়তি নাস্তাগুলো কতটা নিয়মিত আপনি খান? এগুলো কি বাসায় তৈরী খাবার, নাকি বাইরের খাবার? এই খাবারগুলো কি আপনি বন্ধু-বান্ধব, পরিবারের সদস্য, আত্মীয় -স্বজন ,পাড়া -প্রতিবেশীদের দ্বারা অনুপ্রাণিত হয়ে খান?)

Taking meal during menstruation:
(মাসিকের সময় যেসব খাবার গ্রহণ করেন)

a. How do you feel appetite during your menstruation? (probe: why do you feel so? skipping meal or skipping particular type of food, desired to take any special food etc)

মাসিকের সময় খাবারের প্রতি আপনার রুচি কেমন থাকে? (প্রোব করুন: আপনার এরকম লাগে কেন ? এই সময় কি কোনো বিশেষ খাবার খাওয়া থেকে বাদ দেন কিনা, অথবা কোনো বিশেষ খাবার খেতে ইচ্ছা করে কিনা?)

b. Is there any food item that you are told not to take during this time? (prob: by your family members/ relatives/ peers/ neighbors)
এমন কি কোনো খাবার আছে যা আপনাকে দুপুরের খাবার হিসেবে খেতে নিষেধ করা হয়েছে? (যদি নিষেধ করে থাকে তবে কে করেছে? পরিবারের কি কোনো সদস্য/ আত্মীয়-স্বজন/ কোনো বন্ধু-বান্ধব/ পাড়া-প্রতিবেশী?)

Annex: 4: Different Food Items under 16 Food Groups

Serial Number	Food Group	Food Items
**1**	Cereals	corn/maize, rice, wheat, sorghum, millet or any other grains or foods made from these (e.g. bread, noodles, porridge or other grain products) + insert local foods e.g. ugali, nishima, porridge or paste
**2**	White roots and tubers	white potatoes, white yam, white cassava or other foods made from roots
**3**	Vitamin A rich vegetables and tubers	pumpkin, carrot, squash or sweet potato that are orange inside + other locally available vitamin A rich vegetables (e.g. red sweet pepper)
**4**	Dark green leafy vegetables	dark green leafy vegetables including wild forms + locally available vitamin A rich leaves such as amaranth, cassava leaves, kale, spinach
**5**	Other vegetables	other vegetables (e.g. tomato, eggplant, onion) + other locally available vegetables
**6**	Vitamin A rich fruits	ripe mango, cantaloupe, apricot (fresh or drie), ripe papaya, dried peach, and 100% fruit juice made from these + other locally available vitamin A rich fruits
**7**	Other fruits	other fruits, including wild fruits and 100% fruit juice made from these
**8**	Organ meat	liver, kidney, heart or other organ meats or blood-based foods
**9**	Flesh meat	beef, pork, goat, lamb, chicken, duck, rabbit, game, other birds, insects
**10**	Eggs	eggs from chicken, duck, guinea fowl or any other egg
**11**	Fish and sea foods	fresh or dried fish or shell fish
**12**	Legumes, nuts and seeds	dried beans, dried peas, lentils, nuts, seeds or food made from thses (e.g. peanut butter)?
**13**	Milk and milk products	milk, cheese, yogurt or other milk products?
**14**	Oils and fats	Oil, fats or butter added to food or used for cooking
**15**	Sweets	sugar, honey, sweetened soda or sweetened juice drinks, sugary foods such as chocolates, candies, cookies, cakes etc?
**16**	Spices, condiments, beverages	spices (black pepper, salt), condiments (soy sauce, hot sauce), coffee, tea, alcoholic beverages
