# Supplementary material for: Dietary diversity modification through school-based nutrition education among Bangladeshi adolescent girls: A cluster randomized controlled trial
Source: PLoS One. 2023 Mar 8;18(3):e0282407. doi: 10.1371/journal.pone.0282407 (PMC9994752; doi:10.1371/journal.pone.0282407)
Supplement: S1 Appendix — (PDF) [file pone.0282407.s003.pdf]

কিশোরীদের খাদ্যাভ্যাস পরিবর্তনে বিদ্যালয় ভিত্তিক পুষ্টি বিষয়ক শিক্ষা

এক দিনের অর্থাৎ ২৪ ঘন্টায় আমাদের খাদ্য তালিকায় খাদ্য বৈচিত্র্য রক্ষার জন্য নিম্নে বর্ণিত ১৬টি খাদ্য গ্রুপের অন্তর্ভুক্ত বিভিন্ন উপাদানগুলোর উপস্থিতি থাকা অত্যন্ত প্রয়োজন

	খাদ্যের গ্রুপ	উপাদান
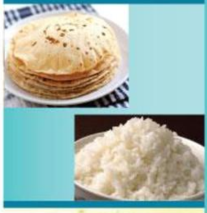	1 শ্বেতসার/ সিরিয়াল	ভাত, রুটি, পরোটা, ভুট্টা, যব, অথবা এগুলো থেকে তৈরী খাবার যেমন, মুড়ি, চিড়া, পাউরুটি, নুডুলস, পাস্তা, জাউ, সুজি, নোনতা বিস্কুট
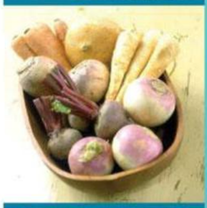	2 সাদা শিকড় এবং কন্দ	গোল আলু, মিষ্টি আলু, মেটে আলু, কেশর আলু, কচু, কচুর মুখী, ওলকচু, শালগম, শালুক
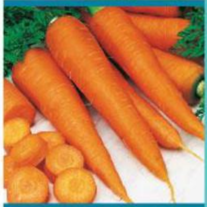	3 ভিটামিন এ সমৃদ্ধ সবজি ও কন্দ	মিষ্টি কুমড়া, গাজর, কমলা রঙের মিষ্টি আলু, লাল ক্যাপসিকাম
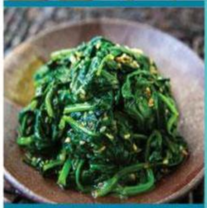	4 গাঢ় রঙের শাক সবজি	লাল শাক, পাতা কপি, ডাটা শাক, কলমি শাক, পালং শাক, কচু শাক, লাউ শাক, কুমড়া শাক, পাট শাক, পুঁই শাক, ধনে পাতা
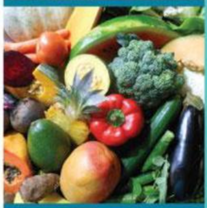	5 অন্যান্য সবজি	টমেটো, পেঁয়াজ, বেগুন, শিম, বীট, করলা, ফুলকপি, কচি ভুট্টা, শশা, সবুজ ক্যাপসিকাম, হলুদ ক্যাপসিকাম, মাশরুম, ঢেঁড়শ, মটরশুঁটি (সবুজ), মুলা, চিচিঙ্গা, লাউ, চালকুমড়া, ধুন্দল, সবুজ পেঁপে, কাকরোল, পটল, ঝিঙ্গা, কচুর লতি, সজিনা ডাটা, শাপলা
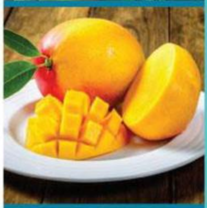	6 ভিটামিন এ সমৃদ্ধ ফল	পাকা আম, বাঙ্গি, পাকা পেঁপে, তরমুজ এবং এগুলো থেকে তৈরী ১০০% ফলের জুস
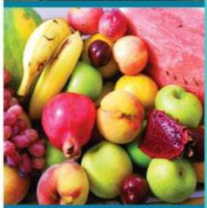	7 অন্যান্য ফল	আপেল, কাঁচা আম, কলা, কমলা, চাইনিজ কমলা, মালটা, নারিকেল, তাল, আমড়া, খেজুর, আঙ্গুর, পেয়ারা, আমলকি, জলপাই, কাঁঠাল, লিচু, জাম, লেবু, নাশপাতি, আতা, আনারস, ডালিম/ বেদানা/ আনার, কামরাঙ্গা, স্ট্রবেরি, তেঁতুল, তরমুজ, লটকন, বেল, কদবেল এবং এগুলো থেকে তৈরী ১০০% ফলের জুস
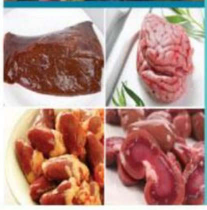	8 প্রাণীর শরীরের ভিতরের অঙ্গের মাংস	কলিজা, ফ্যাপসা (ফুসফুস), গুইজ্জা (কিডনি/বৃক্ক), হৃদপিন্ড (পরান), গিলা, মগজ, ভুঁড়ি ইত্যাদি

	খাদ্যের গ্রুপ	উপাদান
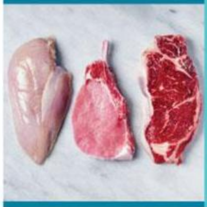	9 মাংস	গরু, মহিষ, ছাগল, ভেড়া, মুরগি, হাঁস, কোয়েল, কবুতর
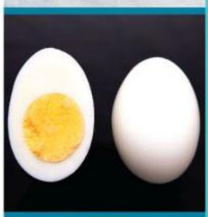	10 ডিম	মুরগির ডিম, হাঁসের ডিম, কোয়েল পাখির ডিম অথবা অন্য যে কোনো পাখির ডিম
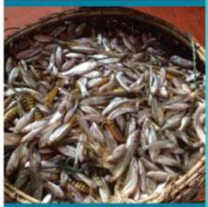	11 মাছ এবং সামুদ্রিক খাদ্য	সব রকমের মাছ (ছোট ও বড়), শুঁটকি মাছ, চিংড়ি, সামুদ্রিক মাছ (কোরাল, লইট্টা, রূপচাঁদা ইত্যাদি), মাছের ডিম (পর্যাপ্ত পরিমাণ)
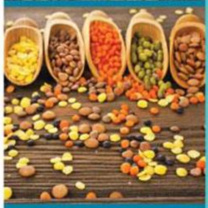	12 ডাল, বীজ ও বাদাম	শুকনো শিমের বীচি, শুকনো মটরশুঁটি, ডাল, চীনা বাদাম, কাজু বাদাম, কাঠ বাদাম, পেস্তা বাদাম, শিমের বীচি, মটরশুঁটি, চটপটি, ঘুঘনি, ছোলা, কাঁঠালের বীচি
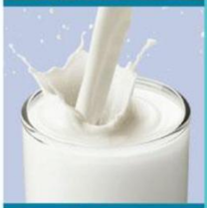	13 দুধ এবং দুগ্ধজাত খাবার	দুধ, পনির, দই, ক্ষীরসা, পায়েস এবং অন্যান্য দুধ জাতীয় খাবার
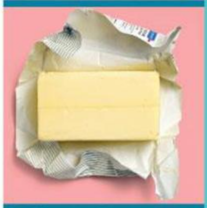	14 তেল ও চর্বি	খাবারে যোগ করা বা রান্নায় ব্যবহৃত ভোজ্য তেল, চর্বি (গরুর চর্বি, মাছের তেল), ঘি, মাখন, মার্জারিন, মেয়নেজ, পাম অয়েল
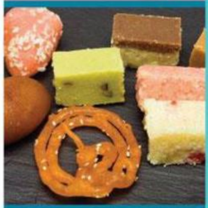	15 মিষ্টি অথবা মিষ্টান্ন	চিনি, মধু, গুঁড়, মিষ্টি সফ্ট ড্রিংক, বোতলজাত জুস যেমন ম্যাংগো জুস, চিনিযুক্ত খাবার যেমন - চকলেট, ক্যান্ডি, মিষ্টিবিস্কুট, কেক, মিষ্টি পিঠা, পেস্ট্রি, সেমাই, হালুয়া, আইসক্রিম, মিষ্টি/ মিঠাই
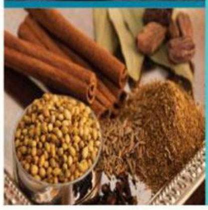	16 মশলা, আচার, সস, পানীয়	মশলা যেমন মরিচ, গোল মরিচ, হলুদ, রসুন, আদা, ধনিয়া, জিরা, সরিষা, কনডিমেন্ট যেমন টেস্টিং সল্ট, আচার, সস, কেচাপ, কাসুন্দি, ধনে পাতার চাটনি, পুদিনা পাতা, পানীয় যেমন চা, কফি, পান, সুপারি ইত্যাদি

icddr,b

Food group no	Food group	Examples
1	CEREALS	rice, ruti, parata, wheat, barley or foods made from these like puffed rice, chira, bread, noodles, pasta, jau, suji, salted biscuits
2	WHITE ROOTS AND TUBERS	white potatoes, sweet potato, saffron potato, colocasia, white yam, white cassava, turnip, shaluk or other foods made from roots
3	VITAMIN A RICH VEGETABLES AND TUBERS	pumpkin, carrot, orange sweet potato, red capsicum
4	DARK GREEN LEAFY VEGETABLES	amaranth, kale, cassava leaves, different types of spinach, coriander leaves
5	OTHER VEGETABLES	tomato, onion, eggplant, bean, bitter melon, cauliflower, green wheat, cucumber, green capsicum, yellow capsicum, mushroom, lady's finger, pea, radish, chichinga, gourds, green papaya, colocasia's young leaves, moringa sticks, water lily
6	VITAMIN A RICH FRUITS	ripe mango, cantaloupe, ripe papaya, watermelon, and 100% fruit juice made from these
7	OTHER FRUITS	apple, green mango, banana, orange, Chinese orange, malta, coconut, palm, dates, grapes, guava, gooseberry, olive, jackfruit, litchi, blackberry, lemon, pear, custard apple, pineapple, pomegranate, strawberry, lotkon, wood apple, other fruits, including wild fruits and 100% fruit juice made from these
8	ORGAN MEAT	liver, kidney, heart or other organ meats or blood-based foods
9	FLESH MEATS	beef, lamb, goat, chicken, duck, pigeon, other birds
10	EGGS	eggs from chicken, duck or any other egg
11	FISH AND SEAFOOD	fresh or dried fish all types of fish (big, small), shellfish, sea fish, dried fish, fish eggs
12	LEGUMES, NUTS AND SEEDS	dried beans, dried peas, lentils, peanuts, cashew nuts, almonds, wood nuts, pea, chickpea, or foods made from these (eg. chatpati, peanut butter)
13	MILK AND MILK PRODUCTS	milk, cheese, yogurt or foods made from dairy products – porridge, payesh, kheer
14	OILS AND FATS	oil, fats, butter, mayonnaise, and margarine added to food or used for cooking
15	SWEETS	sugar, honey, sweetened soda or sweetened juice drinks, sugary foods such as chocolates, candies, cookies and cakes
16	SPICES, CONDIMENTS, BEVERAGES	spices (red pepper, black pepper, salt), ginger, garlic, turmeric, coriander, cumin, wasabi, pickles, condiments (soy sauce, hot sauce), coffee, tea

Our Poster's English Version contains 16 food groups representing dietary diversity
