# Supplementary figures and images for: Dietary diversity modification through school-based nutrition education among Bangladeshi adolescent girls: A cluster randomized controlled trial
Source: PLoS One. 2023 Mar 8;18(3):e0282407. doi: 10.1371/journal.pone.0282407 (PMC9994752; doi:10.1371/journal.pone.0282407)

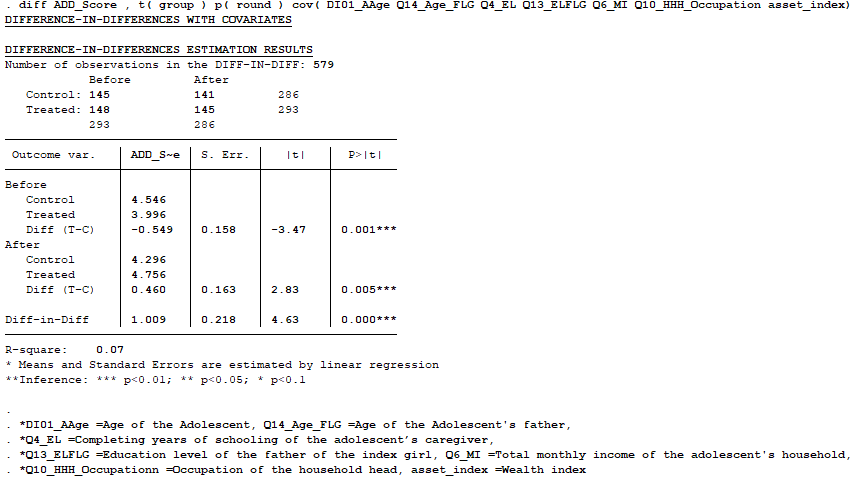

Supplement: S2 Appendix — (TIFF) [file pone.0282407.s004.tiff]
