# Supplementary material for: Dietary diversity modification through school-based nutrition education among Bangladeshi adolescent girls: A cluster randomized controlled trial
Source: PLoS One. 2023 Mar 8;18(3):e0282407. doi: 10.1371/journal.pone.0282407 (PMC9994752; doi:10.1371/journal.pone.0282407)
Supplement: Attachment — Submitted filename: Response to Reviewers.docx [file pone.0282407.s005.docx]

Responses to reviewers’ comments

We appreciate the very thoughtful reviews of the previous version of the manuscript. We have updated the text in response to the reviewers’ queries and feedback. A point-by-point response to each of the reviewers’ comments is included below. We believe these changes have substantially improved the manuscript. We hope you will find this revised manuscript appropriate for publication in PLOS ONE. Many thanks for your consideration.

Reviewer #1: The sample size needs rationale based on intra class correlation in the cluster environment. They state that they calculated sample size based on the effect size of 20 percentage point using the formula of sample size estimation for conventional cluster randomized control trial. They do not state the anticipated power based on this approach.

The main result is seen in Table 3 using the DID which revealed that the mean dietary diversity score among the adolescent girls was likely to be increased by 1 unit due to intervention. The authors give no explanation as to the clinical significance of this difference of one unit. They do, in fact, discuss some limitations of the study seen in the discussion section.

They did state that during performing the DID they adjusted for adolescent’s age, adolescent girls’ father’s age, years of schooling of caregiver, adolescents’ father’s years of schooling, household’s monthly income, household head’s occupation and asset index. This data should be presented as to the significance of these factors, if any.

Response: Thank you for your valuable suggestion. We have stated anticipated power this time in the updated manuscript (mentioned in the “Sample size” part under the “Methods and Materials” section).

Thank you once again for noting the issue of explanation as to the clinical significance of explanation as to the difference of one unit change in mean dietary diversity score. In this revised manuscript we have explained it under the result section.

During performing DID analysis, we adjusted the following variables: adolescent’s age, adolescent girls’ father’s age, years of schooling of caregiver, adolescents’ father’s years of schooling, household’s monthly income, household head’s occupation and asset index in the DID model. As you suggested, we have presented these as supporting information (S2 Appendix) in this revised manuscript.

Reviewer #2: General comments:
The aim of this study was to measure the efficacy of school-based nutrition education on dietary diversity of the adolescent girls in Bangladesh. The paper is interesting, but the measuring or evaluating the effectiveness of nutrition education is something very compromising, especially because the type of nutrition education or the pedagogical basis are not specified. The execution period of the community trial is striking, which was shortly before the COVID-19 pandemic began and the study ended in the total closure of schools (September 2020), which had an effect on the results, and therefore affects the conclusion reached with this research.

Specific comments:

INTRODUCTION
- In the introduction, "nutritional education" is not defined and the theoretical references of the educational intervention are not established.
- The hypothesis of the investigation is not justified with valid antecedents.

Response: Thank you for your valuable suggestions. We have provided definition of nutrition education with theoretical references and hypothesis of the investigation has been justified in “Introduction” section according to your suggestion, in the revised manuscript.

MATERIALS AND METHODS:
Study design and study population
Within its inclusion criteria, indicators of eating behavior disorders are not mentioned, which is more frequent in adolescent women.
Specify the characteristics of urban and rural schools, and the contexts that could have defined a lower or higher access to food, especially in the stage of the COVID-19 pandemic.

Response: Thank you for your comment. We totally agree with you that eating behavior disorders are more frequent in adolescent women. However, this is one of the limitations of our study that we could not collect data regarding this because we had to make our questionnaire brief and short to finish the interview of the participants (adolescent girls) within a limited time (some particular times from school hours). We had to request teachers to give us some separate times when the interview would be conducted (we mentioned this limitation under the “Discussion” section in the updated manuscript). Our questionnaire was divided into 2 parts: questionnaire for adolescent girls at schools and questionnaire for the mother or caregiver of the index adolescent girl at household level. The questionnaire for adolescent girls consisted of demographic information of the index adolescent girls, dietary diversity, menstrual history and health and nutritional status.

As you suggested, we have specified the characteristics of urban and rural schools in the revised manuscript. However, our baseline survey was conducted in January 2020 when there was no COVID-19 pandemic situation. As we used the same questionnaire in baseline and endline, so we did not take any data representing the contexts that could have defined a lower or higher access to food, especially in the stage of the COVID-19 pandemic. It is one of the limitations (mentioned in the limitation part of under “Discussion” section).

INTERVENTION COMPONENTS
- Apparently the meetings with parents were only informative, since the educational strategy used, the duration of the sessions, the materials used and the evaluation or follow-up carried out with the parents are not detailed.

Response: Thank you for your comment. An hour-long nutrition education session (60 minutes to 80 minutes long) was provided among the girls of each grade once in a week by the trained female staff who received training on how to impart nutrition education among adolescent girls, from the investigators of the study (it has been mentioned in the “Nutrition education session” part in the updated manuscript).

As per your suggestions, we have described the materials used and the evaluation carried out in the revised manuscript (it has been mentioned in ‘Nutrition education session and methods and materials section). We have also provided the material- a picture of our poster containing FAO recommended 16 food groups representing dietary diversity as supporting information – S1 Appendix.

- It is necessary to describe the pedagogical or psychoeducational strategy used in the nutritional education sessions, how the contents of the 8 sessions were defined? and what was expected to be achieved with each one of them? As well as the evidence of the learning achieved.

Response: Thank you for your valuable suggestions. We have described how the contents of the 8 sessions were defined in the “Nutrition education session” section under “Materials and methods” of the revised manuscript. We also described pedagogical strategy and the evidence of learning achieved in the revised manuscript.

We conducted quiz session in every week before starting the next session to speculate how much they could understand about the previous session (mentioned in the “Nutrition education session” part in the updated manuscript). Except for the quiz, we did not incorporate any other evidence-generating tool by which we could measure their knowledge about the given intervention, and it is one of our limitations worth to be mentioned (mentioned in the limitation part under the “Discussion” section in the updated manuscript). However, to enhance compliance with the intervention, monitoring of intervention implementation was conducted by supervisory visits to schools and periodic meetings with the school principal, and teachers and by facilitating the peer-education.

- It is necessary to know the messages and materials used to promote dietary diversity, describe how the messages were defined, and how the exposure to information in parents and adolescents was controlled.

Response: As you suggested we have described the messages and materials used to promote dietary diversity in the ‘Nutrition education session’ section and ‘Information, education and communication materials’ section under the heading of ‘Methods and materials. As per your suggestions, we also have described how the exposure to information in parents and adolescents was controlled in the section of study design and study population under the heading of ‘Methods and martials’.

- It is necessary to clearly define the variable "dietary diversity".

Response: Thank you for your valuable suggestion. We have provided definition of dietary diversity in the updated manuscript (mentioned in the “Trial outcome” section of the updated manuscript).

- The 24-hour recall questionnaire only reports information on the diet of the previous day, so the diversity of the diet cannot be evaluated, or it can be used if it is applied repeatedly during 3 days of the week.

Response: Thank you for your comment. We used FAO recommended dietary diversity questionnaire. The recall period of 24 hours was chosen by FAO as it is less subject to recall error, less cumbersome for the respondent, and also conforms to the recall time period used in many dietary diversity studies. Moreover, analysis of dietary diversity data based on a 24-hour recall period is easier than with longer recall period (Reference: Kennedy G, Ballard T, Dop M. Guidelines for Measuring Household and Individual Dietary Diversity. Rome, Italy: Division NaCP; 2010.)

- Considering that there is no cut-off point to indicate adequate or inadequate dietary diversity, it is arbitrary to use the mean score without considering the nutritional quality of the food, the cultural relevance and the accessibility of the food.

Response: We agree with your comment. There is no established cut-off to measure dietary diversity scores for adolescent girls. According to FAO guidelines (Reference: Kennedy G, Ballard T, Dop M. Guidelines for Measuring Household and Individual Dietary Diversity. Rome, Italy: Division NaCP; 2010), some food groups in the dietary diversity questionnaire are combined into a single food group. Following the guidelines, we combined 16 food groups into 9 food groups. Then dietary diversity score was calculated which was normally distributed. For this reason, we calculated the mean score.

Dietary diversity score (DDS) is defined as a number of individual food groups consumed over a given period of time (FHI F. 360. Minimum dietary diversity for women: a guide for measurement Rome. Italy: FAO; 2016). It reflects the quality of diet at the household or individual level. In addition, DDS is a measure of food security, nutrition information, early warning system and target of intervention at Global or national level [Kennedy G, Ballard T, Dop MC. Guidelines for measuring household and individual dietary diversity: food and agriculture Organization of the United Nations; 2011]. By following FAO’s “Guideline for measuring household and individual dietary diversity, we calculated the dietary diversity score in both the control and intervention arm which reflected the quality of diet at the household or individual level. However, it is one of our limitations that we did not consider the cultural relevance and the accessibility of the food.

Results

- At the end of the intervention, results are presented that indicate a higher mean dietary diversity score in the intervened group compared to the control group (Fig 3), but it is not possible to interpret what these differences would constitute in terms of dietary diversity.

Response: We agree with your comment. We only described our result that mean dietary diversity score was higher among the intervention group than the control group at the endline survey. For presenting the effect of intervention, we performed DID model and in the revised manuscript, we have shown the variables adjusted in the model.

- In the difference in difference (DID) analysis (Table 3), there is speculation of an increase in one unit of dietary diversity in the intervened group, but controlled models are required to verify if these differences persist when correcting for income, the educational level of the caregiver, and assess the effect of the COVID-19 pandemic on food availability and access.

Response: Thank you for your valuable comment about the DID analysis. During performing DID analysis, we adjusted the following variables - adolescent’s age, adolescent girls’ father’s age, years of schooling of caregiver, adolescents’ father’s years of schooling, household’s monthly income, household head’s occupation and asset index in the DID model and this has been given as supporting information (S2 Appendix) in the updated manuscript.

Thank you for noting the issue on the effect of the COVID-19 pandemic on food availability and access. Actually, our baseline survey was conducted in January 2020 when there was no COVID-19 pandemic situation. As we used the same questionnaire in baseline and endline, so we did not collect data representing the effect of the COVID-19 pandemic on food availability and access. It is one of our limitations (mentioned in the limitation part of the “Discussion” section).

Discussion
The following statement "Our study demonstrated that school-based nutrition education has potential to increase dietary diversity among the adolescent girls", requires that what is "information on nutrition" and "nutritional education" be clearly differentiated; since in this research no an educational intervention was justified.

Response: Thank you for your valuable suggestions. We have corrected the above-mentioned issues in the second paragraph under “Discussion” section of revised manuscript.

Conclusion
It is not desirable that the fact of reviewing some nutrition content in school lasting one hour for 8 weeks can be considered a nutritional education strategy, and a public policy be suggested based on it.

Response: Thank you for your comment. We wanted to establish a sustainable model of school-based nutrition education for improving dietary diversity among adolescent girls. Scaling up of an innovative intervention and its sustainability is essential for an effective and longer-term impact of the intervention. We found that some previous studies provided food as an intervention among school-going adolescent girls but the withdrawal of direct food under the school feeding program (SFP) is very likely to have negative impact on the sustainability of the program [Moucheraud C, Sarma H, Ha TTT, Ahmed T, Epstein A, Glenn J, et al. Can complex programs be sustained? A mixed methods sustainability evaluation of a national infant and young child feeding program in Bangladesh and Vietnam. BMC Public Health. 2020;20(1):1361. Epub 2020/09/06. doi: 10.1186/s12889-020-09438-2. PubMed PMID: 32887601; PubMed Central PMCID: PMCPMC7487916.]. Our study has focused on behavior change of the adolescent girls through nutrition education at school setting. However, the shorter duration of the intervention in our study could not demonstrate whether it could change behavior of the adolescent girls in increasing dietary diversity through school-based nutrition education in a sustainable manner, rather it showed a pathway on how to increase dietary diversity at school setting.
