# Supplementary material for: Dietary diversity modification through school-based nutrition education among Bangladeshi adolescent girls: A cluster randomized controlled trial
Source: PLoS One. 2023 Mar 8;18(3):e0282407. doi: 10.1371/journal.pone.0282407 (PMC9994752; doi:10.1371/journal.pone.0282407)
Supplement: Attachment — Submitted filename: Response to reviewers.docx [file pone.0282407.s006.docx]

We appreciate the very thoughtful reviews of the previous version of the manuscript. We have updated the text in response to the reviewers’ queries and feedback. A point-by-point response to each of the reviewers’ comments is included below. We believe these changes have substantially improved the manuscript. We hope you will find this revised manuscript appropriate for publication in PLOS ONE. Many thanks for your consideration.

Journal Requirements:…………………………………………………………………

Please review your reference list to ensure that it is complete and correct

If you have cited papers that have been retracted, please include the rationale for doing so in the manuscript text, or remove these references and replace them with relevant current references. Any changes to the reference list should be mentioned in the rebuttal letter that accompanies your revised manuscript. If you need to cite a retracted article, indicate the article’s retracted status in the References list and also include a citation and full reference for the retraction notice.

Response: Thank you for your comment. We have updated the reference list accordingly.

Additional Editor Comments:……………………………………………………….

In general, the authors have given a satisfactory response to all the comments made by the reviewers;

however, there are some texts that must be clarified or eliminated according to the methodology and scope of the research results.

**INTRODUCTION**

The definition of nutrition education provided in the introduction is limited but fits the model developed in the intervention. It is suggested on future occasions to include an expert in health education.

**Response:** Thank you for your valuable suggestion. If the intervention is retested, we will recommend including an expert in health education.

**Comment on TITLE:**

Dietary Diversity Modification through School-Based Nutrition Education among Bangladeshi Adolescent Girls: A Cluster Randomized Controlled Trial
It does not meet the methodological criteria to be a cluster controlled clinical trial, since there are serious limitations in the research, which have been detailed in the comments made by the reviewers, so it is suggested to modify by: Dietary Diversity Modification through School-Based on Nutrition Knowledge among Bangladeshi Adolescent Girls: A Cluster Randomized Non-Controlled Trial.

**Response:** We agree with you that it doesn’t fully meet the criteria of a cluster-controlled clinical trial. It was a school-based trial, so there were some limitations.

In our revised manuscript, we described in the ‘Study Design and Study Population” part under the “Methods and Material” section that we tried our best to maintain sufficient buffer zone between control and intervention schools. In the rural setting, the intervention and control schools were located in two completely different sub-districts. In the case of urban schools, even though intervention and control schools were located in Rangpur Sadar (same sub-district), they were located far from one another – the intervention school was located 4.9 km away from the control school.

We also agree that the number of clusters were also limited. However, in support of our research, we would like to mention ha there was another two‑armed cluster randomized controlled trial (RCT) conducted in 10 control and 10 intervention villages in Mangochi, Southern Malawi. In this study, they used intervention similar to us. They provided supplementary nutrition education, dietary counselling and routine ANC services in the intervention group whereas the controls received only routine ANC services (*Reference- Katenga-Kaunda, L.Z., Kamudoni, P.R., Holmboe-Ottesen, G. et al. Enhancing nutrition knowledge and dietary diversity among rural pregnant women in Malawi: a randomized controlled trial. BMC Pregnancy Childbirth 21, 644 (2021).* [*https://doi.org/10.1186/s12884-021-04117-5*](https://doi.org/10.1186/s12884-021-04117-5)*)*

Based upon all these contexts, we request you to consider our title as it is.

Comment on MATERIALS AND METHODS:

Sample Size: A satisfactory answer has been given; therefore, 150 girls are required for rural schools, and 150 for urban schools, or otherwise control by type of locality. Place this section immediately after randomization.

**Response:** Thank you for your suggestion. We placed this section after randomization in the updated manuscript. We placed the “Figure 1. Trial flow diagram” (describing clear view of randomization and sample) immediately after “Randomization section” as you suggested.

Comment on MATERIALS AND METHODS

Apparently there is no cut-off point to establish the diversity of the diet, so the greatest limitation of the study is that the increase of 1 point in the diversity of the diet in the intervened group does not have an interpretation or clinical significance.

**Response:** Thank you for your valuable comment. We agree with you that the increase of 1 point in the diversity of the diet in the intervened group does not have clinical significance. We have included this as one of the limitations under the limitation part of discussion section in the revised manuscript**.** However, although mean dietary diversity score was increased only 1 point in intervention arm compared to control arm, this increase was statistically significant after all other variables were adjusted. Moreover, our intervention on school-based nutrition education increased the mean score of dietary diversity that could be minimal, but it was significant within a shorter period and with many challenges, and the results indicated increase of some food groups in the intervention group which are – “other vitamin A rich fruits and vegetables”, “organ meat”, “legumes, nuts and seeds” and “milk and milk products”.

Comment on DISCUSSION: The authors must assume a clear position based on the scientific literature on what a point of difference in the diversity of the diet between the intervened and control group means, you must cite other studies that have applied the same scale in other populations.

**Response:** Thank you for your comment. We would like to mention that dietary diversity score was determined by adding the number of food categories consumed by each participant over the previous 24 hours. The questionnaire contained 16 food groups and we analyzed 9 food groups because we combined certain food groups into a single food group for analytical purpose (according to FAO guideline).

Our study population was 10-14 years of adolescent girls. According to FAO’s “Guidelines for measuring household and individual dietary diversity”, there is no established cut-off point to measure dietary diversity for adolescent girls of 10-14 years of age (*Reference number 27 in the revised*). The guideline set cut-off of minimum dietary diversity for 15-49 years of women and some other studies worked on “improving dietary diversity” followed this cut-off as their study participants age followed 15-49 years of age ((*Reference number 26, 30 in the revised manuscript) and also another study was done in Malawi* - (*Reference- Katenga-Kaunda, L.Z., Kamudoni, P.R., Holmboe-Ottesen, G. et al. Enhancing nutrition knowledge and dietary diversity among rural pregnant women in Malawi: a randomized controlled trial. BMC Pregnancy Childbirth 21, 644 (2021).* [*https://doi.org/10.1186/s12884-021-04117-5)*](https://doi.org/10.1186/s12884-021-04117-5))).

Comment on DISCUSSION: It remains to argue the role that nutrition education plays within the school curriculum and how it could be used to increase dietary diversity in the long term.

**Response:** Thank you for your concern. Alike our intervention study, some studies conducted in similar settings indicated that along with the regular curriculum in secondary schools, nutrition education can be added on to improve the dietary diversity of adolescent girls *[Reference number -26,29,30 in manuscript]*. They suggested that school-based nutrition and health education should be a part of comprehensive school health programs to reach both the students and their families *[Reference number - 26 in the revised manuscript]*.

In low- and middle-income countries, the school feeding program (SFP) has been found instrumental to increasing dietary diversity of adolescent girls but such a program has some limitations including irregular supply and storage of materials (i.e., food items) and loss of educational time at school [*Reference number* - 23]. Withdrawn of direct food under the school feeding program (SFP) is very likely to have a negative impact on the sustainability of the program [*Reference number* - 33]. In our study, unlikely to school feeding program, we used existing materials of schools like – laptops, projectors. So, it can be said that, this kind of intervention has high chance to be sustainable in long run. Our trained staffs provided nutrition education sessions in our study. To maintain sustainability, short-term teacher’s training of existing school is enough to deliver the nutrition education session. (We have updated these parts in our “Discussion” section in the updated manuscript).

Comment on DISCUSSION: An explanation should be included as to why the revised knowledge in nutrition education had the effect of increasing the consumption of this type of food, such as visor meat and the consumption of legumes, seeds and nuts.

**Response:** Thanks. We have included the explanation as follows in the revised manuscript:

Due to our intervention, consumption of some food groups was increased among intervention arm than in control arm in endline – “other vitamin A rich fruits and vegetables”, “organ meat”, “legumes, nuts and seeds” and “milk and milk products”. Parents’ meetings and nutrition education sessions may be the main reasons of improving the consumption of some food groups.

During parent’ meeting, we discussed about the necessity of dietary diversity for young adolescent girls and we described parent’s role in improving dietary diversity by buying and cooking diversified foods for their family members. We provided posters containing 16 food groups representing dietary diversity among adolescent girls and suggested them to hang the poster in the kitchen where their caregivers cook or in front of dining table where all family members gather to eat to practice dietary diversity in everyday life (We highlighted these parts in our “Intervention components” part for your kind reading).

During our nutrition education sessions, we provided detailed description of 16 food groups by showing locally available Bangladeshi food items pictures. We also informed them how to choose diversified diets in low cost (We highlighted these parts in our “Intervention components” part for your kind reading). Students shared with us that they did not know about some food groups which should be eaten for the betterment of health like – organ meat. Lack of knowledge was the main problem to not practicing the diversified food among adolescent girls. That’s why EAT – Lancet Commission also supported that individuals should be educated on healthy diet (Reference no 41 in our updated manuscript)

Comment on DISCUSSION: In addition, it must incorporate the current vision of the planetary diet of eat-lancet to reduce the consumption of proteins of animal origin, and what implications it could have on the growth and nutrition of children and adolescents in Bangladesh.

**Response:** Thank you for your valuable suggestion. We have incorporated a discussion part under discussion section in the updated manuscript according to your suggestion.

**Comment on CONCLUSION:** The scope of the nutrition education intervention is limited, since it lacks pedagogical theoretical support and does not have adequate didactics, so it is suggested to eliminate the recommendation to expand the intervention on a national scale. It should be noted that other elements of the food environment should be included in future studies to improve dietary diversity in adolescent school-age women.

**Response:** Thank you for your valuable comments. As you suggested we have revised conclusions (page 23) section of the manuscript as follows:

‘The shorter duration of the intervention in our study could not demonstrate whether it could change the behavior of adolescent girls in increasing dietary diversity through school-based nutrition education, instead it showed a pathway on how to increase dietary diversity at school setting. With a limited resource, our school-based nutrition education intervention in real settings of the schools has demonstrated its feasibility in improving dietary diversity of the adolescent girls. Since this study was conducted in the COVID-19 situation with many other challenges and limitations it recommends to retest this intervention before scaling up in the larger scale. In retesting, we recommend including more clusters and other elements of the food environment to increase its precision and acceptability.’

Comment on APPENDIX: Include appendix 1 in English and the original language.

**Response:** Thank you for your comment. We followed the FAO recommended 16 food group chart which is available in the “Guidelines for Measuring Household and Individual Dietary Diversity” (it is our *reference number 27*) and considered locally available Bangladeshi foods for developing the poster. In the revised manuscript, we have provided the English one under Appendix-1, indicating with the numbers that present different food groups.
