# Supplementary material for: Dietary diversity modification through school-based nutrition education among Bangladeshi adolescent girls: A cluster randomized controlled trial
Source: PLoS One. 2023 Mar 8;18(3):e0282407. doi: 10.1371/journal.pone.0282407 (PMC9994752; doi:10.1371/journal.pone.0282407)
Supplement: Attachment — Submitted filename: Response to reviewers.docx [file pone.0282407.s008.docx]

Response to reviewers’ comment

Thank you for your valuable comment. Response to the reviewer comments is included below:

Reviewer comment: The authors have made the requested corrections. The main document is attached with some very specific comments, and it is suggested to update the conclusion in the abstract.

Response: Thank you for your valuable comments. As you suggested we have updated the inclusion in the abstract in the revised version of the manuscript.
